# AnnapuRNA: A scoring function for predicting RNA-small molecule binding poses

**DOI:** 10.1371/journal.pcbi.1008309

**Published:** 2021-02-01

**Authors:** Filip Stefaniak, Janusz M. Bujnicki

**Affiliations:** 1 Laboratory of Bioinformatics and Protein Engineering, International Institute of Molecular and Cell Biology, Warsaw, Poland; 2 Institute of Molecular Biology and Biotechnology, Faculty of Biology, Adam Mickiewicz University, Poznan, Poland; New York University, UNITED STATES

## Abstract

RNA is considered as an attractive target for new small molecule drugs. Designing active compounds can be facilitated by computational modeling. Most of the available tools developed for these prediction purposes, such as molecular docking or scoring functions, are parametrized for protein targets. The performance of these methods, when applied to RNA-ligand systems, is insufficient. To overcome these problems, we developed AnnapuRNA, a new knowledge-based scoring function designed to evaluate RNA-ligand complex structures, generated by any computational docking method. We also evaluated three main factors that may influence the structure prediction, i.e., the starting conformer of a ligand, the docking program, and the scoring function used. We applied the AnnapuRNA method for a *post-hoc* study of the recently published structures of the FMN riboswitch. Software is available at https://github.com/filipspl/AnnapuRNA.

## Author summary

Drug development is a lengthy and complicated process, which requires costly experiments on a very large number of chemical compounds. The identification of chemical molecules with desired properties can be facilitated by computational methods. Several methods were developed for computer-aided design of drugs that target protein molecules. However, recently the ribonucleic acid (RNA) emerged as an attractive target for the development of new drugs. Unfortunately, the portfolio of the computer methods that can be applied to study RNA and its interactions with small chemical molecules is very limited. This situation motivated us to develop a new computational method, with which to predict RNA-small molecule interactions. To this end, we collected the information on the statistics of interactions in experimentally determined structures of complexes formed by RNA with small molecules. We then used the statistical data to train machine learning methods aiming to distinguish between RNA-ligand interactions observed experimentally and other interactions that can be observed in theoretical analyses, but are not observed in nature. The resulting method called AnnapuRNA is superior to other similar tools and can be used to predict preferred ligands of RNA molecules and how RNA and small molecules interact with each other.

## Introduction

Ribonucleic acids (RNAs) play a pivotal role in many cellular processes such as transmission of genetic information, sensing and communicating responses to cellular signals, and even catalysis of chemical reactions [1]. These processes are often modulated by other molecules, such as ions or small molecules, making RNA an attractive therapeutic target for new drugs [2–4]. A well-studied and clinically validated example is the bacterial ribosome, a molecular target for many antibiotics [5]. Riboswitches, a class of regulatory RNA structural elements embedded in untranslated regions of mRNAs, comprise another interesting group of RNA targets. These structural elements can directly bind a ligand to regulate gene function without the need for protein cofactors [6,7]. Riboswitches are common in bacterial cells and rarely occur in eukaryotic cells, which makes them suitable targets for new antibacterial drugs. One well-studied example is the FMN riboswitch, which controls expression of genes required for biosynthesis and transport of riboflavin (vitamin B2) in bacteria [8]. Several small-molecule inhibitors of the FMN riboswitch with proven antibacterial properties have been identified. Some of these small molecule inhibitors include compounds such as roseoflavin [9,10] or 5FDQD [11], which are structurally similar to the natural ligand, as well as structurally dissimilar ligands having a different chemotype, for example, ribocil and its derivatives [12]. Apart from riboswitches, other medically-relevant RNAs include viral RNAs (dimerization initiation site of HIV-1 RNA [13]), self-splicing group I introns (e.g., an inhibitor for the *td* intron RNA [14]), group II introns (e.g., inhibitors of group IIB intron splicing [15]), and viral ribozymes (e.g, hepatitis D virus ribozyme inhibition by aminoglycosides [16]) (for details, see [17]).

Elucidating the role of RNA-ligand interactions and design of new RNA-binding molecules can be facilitated by analyzing three dimensional (3D) structures of the RNA of interest or its complex with a ligand. Unfortunately, experimental determination of 3D structures is an intensive task and often has to be supported by computational modeling [18] or performed entirely *in silico* (for review on methods of modeling of ribonucleic acid–ligand interactions see: [19]). These limitations, together with an increasing interest in RNA as a target for therapeutic intervention, highlight the need to develop new methods for predicting the 3D structure of RNA-ligand complexes.

One of the most widely used computational methods used to predict the 3D structures of macromolecules with ligands is molecular docking [20,21]. Many of the currently-available docking programs were initially designed for protein-protein or protein-ligand docking (e.g., as AutoDock [22], AutoDock Vina [23], ICM [24], or iDock [25]) while some of them were later adapted or reparameterized to enable RNA-ligand docking, where RNA is specified as the receptor (Dock6, [26], ICM [27], or AutoDock [28]). A few programs were designed and optimized specifically for docking ligands to RNA. MORDOR allows for both ligand and receptor flexibility and uses a scoring function that estimates the total energy of the complexes and consists of several terms (electrostatic, van der Waals, dihedral angle, torsion angle, bond, and Urey−Bradley) [29]. rDock (formerly: RiboDock) consists of a genetic algorithm stochastic search engine as a pose generator, and intermolecular scoring function validated against protein and RNA targets [30,31]. The main component of this scoring function is a van der Waals potential, an empirical term for attractive and repulsive polar interactions, and an optional desolvation potential that combines a weighted solvent accessible surface area approach. rDock program can also be used for rescoring poses generated by an external tool.

In a portfolio of methods facilitating prediction of 3D RNA-ligand structures, there are also standalone scoring functions specific for RNA-ligand complexes, intended to be used for rescoring of models generated by molecular docking. A separate group of such methods consists of statistical potentials (statistical scoring functions, or knowledge-based potentials), which are derived from an analysis of experimentally solved structures. Pfeffer and Gohlke developed a scoring function named DrugScore^RNA^, which employs a distance-dependent potential calculated on the basis of contacts between ligand and receptor atoms [32]. Both interacting partners are in all-atom representation using Tripos atom types. Similarly, a KScore scoring function, described by Zhao *et al*., is also a distance-dependent potential, which in addition to the standard set of Tripos atom types, uses an extended atom type set to characterize the metal-ligand and water-ligand interactions better. It likewise defines a special atom-typing scheme for nucleic acids, and it was parameterized on ligand complexes with proteins (2422 complexes), DNA (300 complexes), and RNA (97 complexes). Yan and Wang described a distance-dependent scoring function termed SPA-LN, where Tripos atom types are used for RNA and ligand representation [33]. During the optimization of parameters, they focused not only on recapitulating the near-native pose of the ligand but also on the affinity estimation. Improving the affinity predictions was a goal of Chen *et al*., authors of iMDLScore1 and iMDLScore2 functions [34]. They optimized AutoDock4.1 scoring terms using multilinear regression methods and binding affinity data for 45 RNA complexes. Our group developed LigandRNA—an anisotropic distance- and angle-dependent statistical potential [35]. It was derived from a diversified set of 251 experimentally solved RNA-ligand complexes and used all-atom representation with Tripos atom types. It was shown to be superior to scoring functions described earlier in terms of accuracy of finding a near-native conformation of ligands. Chhabra *et al*. in the RNAPosers used a distance-dependent fingerprint to describe a binding pose of a ligand in RNA binding pocket [36]. Data derived from 80 experimentally solved RNA-ligand complexes were used to train a machine-learning algorithm, which was then used to rank docking poses. Similarly to the programs described above, RNAPosers uses an all-atom representation with Tripos atom types.

In this study, we describe AnnapuRNA, a new knowledge-based scoring function designed to evaluate RNA-ligand complex structures, generated by any computational docking method. We also present a benchmark in which we compare AnnapuRNA with other available scoring functions. We present a case study in which we used molecular docking in combination with the AnnapuRNA scoring function to predict a recently published structure of an FMN riboswitch co-crystallized with a new small-molecule inhibitor. Our method successfully predicted the structure of this complex based on the previously published structure of FMN in complex with a different ligand, as well as the low-resolution apo structure of this RNA.

AnnapuRNA is freely available to the scientific community at https://github.com/filipspl/AnnapuRNA.

## Results and discussion

AnnapuRNA is a machine-learning statistical scoring function developed by us for predicting the structure of RNA-ligand complexes with high accuracy. Our program internally uses a coarse-grained representation for both interacting partners—RNA and small molecule ligands. Using coarse-grained representation has been proven successful for simulating various biomolecular systems [37], including folding of the RNA molecule by the SimRNA program, also developed by our group. We derived machine learning models expressing the probability of RNA-ligand interactions, based on the interaction data collected from experimentally solved complexes extracted from the PDB database. AnnapuRNA can be used to identify native-like poses of ligands from the pool of poses generated through molecular docking experiments. It supports outputs from multiple docking programs (such as rDock, iDock, AutoDock Vina, Dock6, among others).

**Fig 1:**
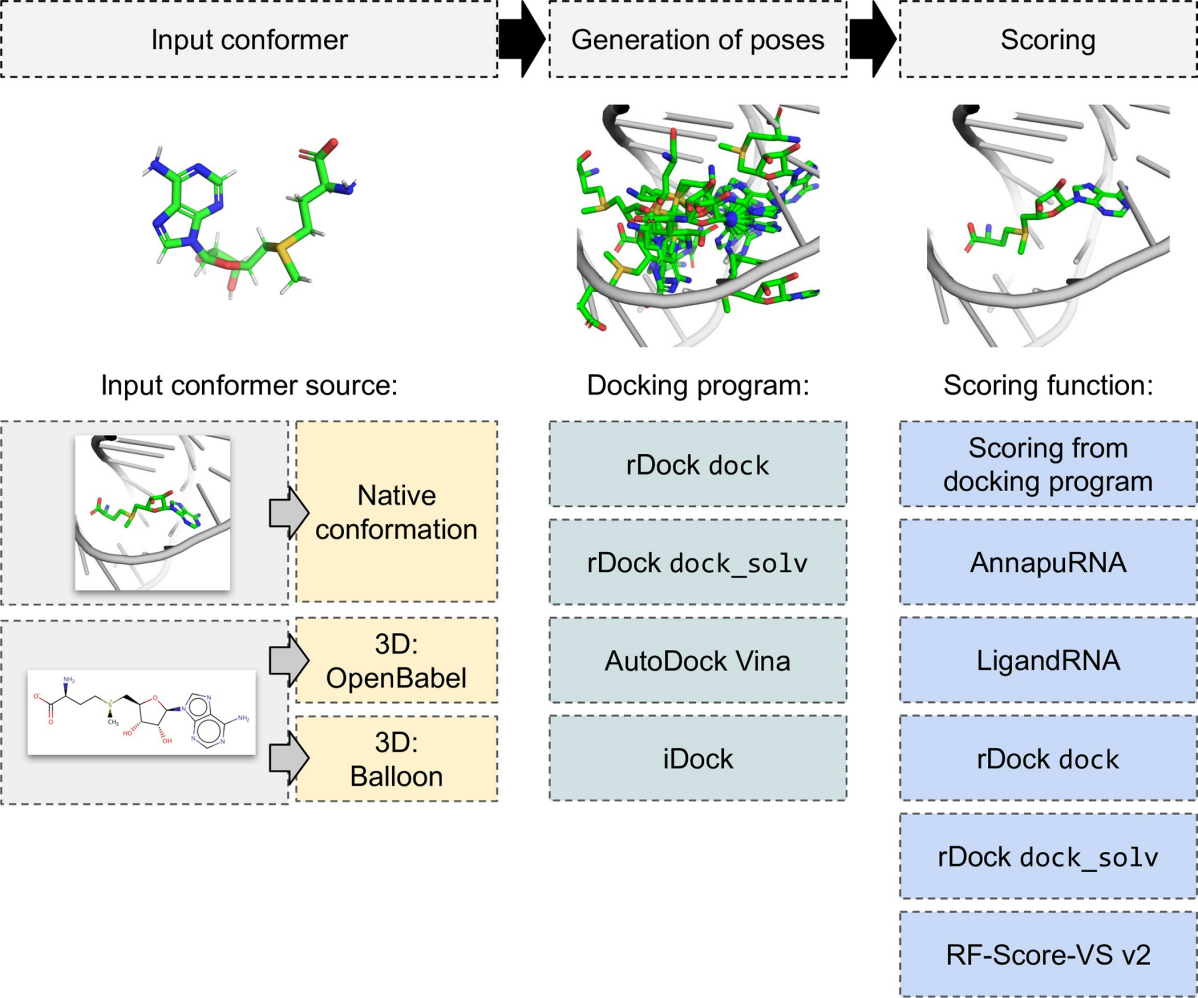
Typical stages of molecular docking applied for interaction predictions for RNA-ligand complexes and methods tested within this publication. The first stage is ligand conformer generation (either using the native conformation extracted from experimentally solved structure or generation of 3D structure), which is followed by molecular docking, and scoring of generated poses.

To evaluate the accuracy of AnnapuRNA in finding near-native structures of RNA-ligand complexes, we carried out extensive benchmarking. We tested three main factors that are likely to influence the outcome of the structure prediction procedure: the starting conformer of a ligand, the docking program, and the scoring function used (see Fig 1). Finally, we used molecular docking for conducting *post-hoc* studies of the recently published structures of the FMN riboswitch.

### Comparison of AnnapuRNA with other scoring methods

**Fig 2:**
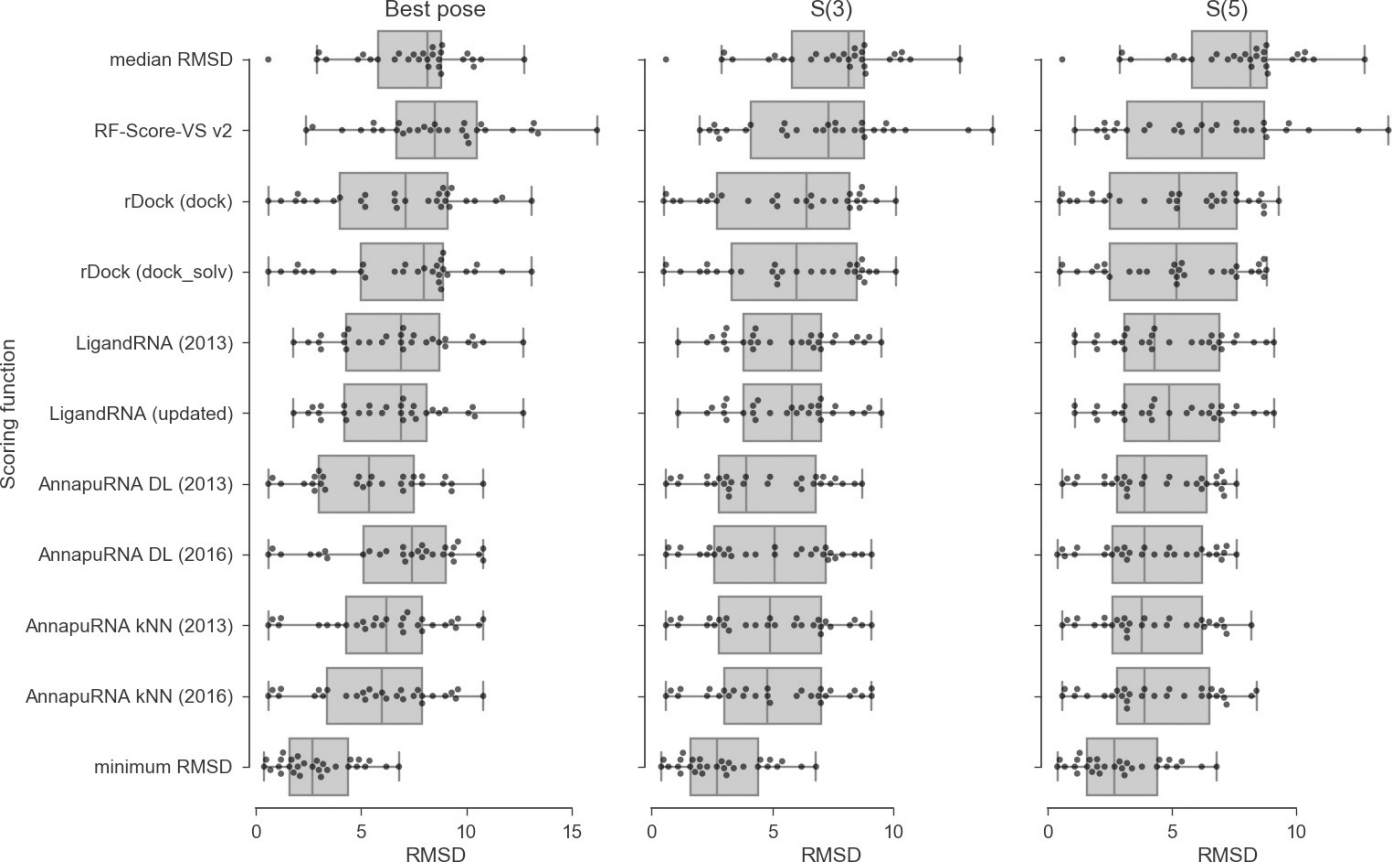
Comparison of the performance of nine scoring functions, expressed as the RMSD between the best-scored pose and the reference pose (left panel), best among the top three scored poses (*S*(3), middle panel), and best among the top five scored poses (*S*(5), right panel). Additional rows represent the median and minimal values of RMSD obtained during docking. Each dot represents one complex from the testing set. Docking was performed using rDock with dock desolvation potential with the native conformation of a ligand as an input.

To compare the performance of AnnapuRNA scoring functions with methods developed earlier, we used a testing set described earlier by Philips *et al*. [35]. AnnapuRNA models were trained on the full “2013” and “2016” training sets. In this analysis, we used a pose of a ligand extracted from the experimentally solved structure as an input conformation and the rDock program with dock desolvation potential for docking. For each complex, we generated a set of 100 poses and applied an external scoring function for rescoring. We tested nine rescoring methods: RF-Score-VS v2 (Random Forest-based scoring function for Virtual Screening, trained for proteins, [38]), rDock scoring functions with two desolvation potentials: dock and dock_solv (these potentials, implemented in rDock docking program, consist of a van der Waals potential, an empirical term for attractive and repulsive polar interactions, and an optional desolvation potential), LigandRNA (a knowledge-based potential derived from ligand-binding sites in the experimentally solved RNA–ligand complexes, obtained using the inverse Boltzmann scheme; this scoring function was used in two versions: the one described in the original publication, called “2013”, and the second, with an updated potential, called “updated” [35], and AnnapuRNA described herein (variants based on two machine learning methods: DL and *k*NN, with two sets of training data: “2013” and “2016”). Regrettably, we were unable to run the DrugScore^RNA^ scoring function, despite contacting the authors and obtaining the source code [32]. Also, the authors of the recently published SPA-LN scoring function [33] were unable to provide us with the working copy of their program nor give access to the dedicated web server. Results expressed as RMSD of the best-selected pose to the reference pose (*S*(1)), *S*(3), and *S*(5) are summarized in the Figs 2 and S10 and S11 Tables. In addition to the results of rescoring, we included data for the median RMSD values obtained during docking (this serves as a negative control) and minimum RMSD values (a positive control).

The average values for all three metrics: *S*(1), *S*(3), and *S*(5) indicate that the AnnapuRNA scoring functions are, in most cases, superior (i.e., having lower values of *S*(n)) to the methods developed previously. For the most strict criteria, which is the average RMSD of the best-scored poses (*S*(1)), the best performing methods were AnnapuRNA version DL trained on the “2013” dataset and AnnapuRNA version *k*NN trained on “2016” dataset (*S*(1) equal to 5.39 and 5.76, respectively). Among the LigandRNA variants, the updated version of the potential (*S*(1) = 6.37) performed better than the older version. Among the rDock variants, the dock desolvation potential (*S*(1) = 6.81) was found to be better.

In this comparison, AnnapuRNA generally outperforms all the other methods, with the only exception of AnnapuRNA DL trained on the “2016” dataset that is outperformed by our previously-built method, LigandRNA in selecting the single best-RMSD pose. The overall best-performing potential is AnnapuRNA DL-2013.

The performance of the ‘protein-focused’ scoring function, RF-Score-VS v2, was found to be worse than all methods tested. Both median and average values for all the three metrics tested are high for this method, with a very high variation in the results. For example, *S*(3) for this method ranges from 2.0 to 14.1, with a standard deviation of 3.2. These findings show that the development of an RNA-specific scoring function is necessary for advancing RNA-ligand docking and modeling of RNA-ligand interactions.

Analysis of the *SR*(X,C) values, *i*.*e*., the rate of the successfully docked poses, reveals that for the most strict success criterion—*SR*(3,2)—the performance of all tested scoring functions isn’t better than the random selection of poses (see: S9 Fig and S13 Table). Values range from 0.00 and 0.03 for RF-Score-VS v2 and LigandRNA, respectively, to 0.14 for AnnapuRNA and rDock (dock), while for randomly selected pose *SR*(3,2) equals 0.12. This result shows that RNA-ligand docking with the currently available software for RNA-ligand pose generation is much more challenging than protein-ligand docking, where success rates are remarkably higher. For example, *SR*(3,2) values reported by Ashtawy and Mahapatra for 22 protein-ligand docking programs and scoring functions are in the range of 0.6–0.9 [39], while for our testing set the maximum possible *SR*(3,2) value is 0.34 (the best possible performance, if the scoring function always selects the pose with the lowest RMSD). Nonetheless, for all *SR* variants tested, the performance of AnnapuRNA is in most cases better than other scoring functions, as well as it surpasses the random selection of poses.

The comparison of LigandRNA and AnnapuRNA scoring functions developed in our group (both variants trained on structures available in 2013, i.e., the “2013” versions), indicates that for most metrics used, AnnapuRNA (the new method) is superior to LigandRNA. This means that based on the same subset of experimentally solved structures available for method training, we obtained a more accurate assessment of interactions using the approach described in this article. Moreover, AnnapuRNA is 5 to 6 times faster than LigandRNA, which is an important factor taken into consideration when processing large datasets (see: S20 Table and S16 Fig). The main differences in the design of these programs include the coarse-grained representation of molecules and the use of a rich set of interaction descriptors combined with machine learning methods in AnnapuRNA, versus full atom representation, two features describing interactions, and the inverse Boltzmann scheme for deriving potential in LigandRNA.

To ensure that the redundancy in the testing set (i.e., the fact that the testing set contains four structures which are similar to ones used for train models, see S2 Table) didn’t favor the AnnapuRNA scoring function, we recalculated the performance for the testing set with four redundant structures excluded. We found that for all functions and metrics but one (namely *SR*(5,2) for AnnapuRNA kNN (2016)), the performance observed for the non-redundant testing set was better than for the initial testing set. Also, the overall rank of scoring methods did not change (see S12 Table).

**Fig 3:**
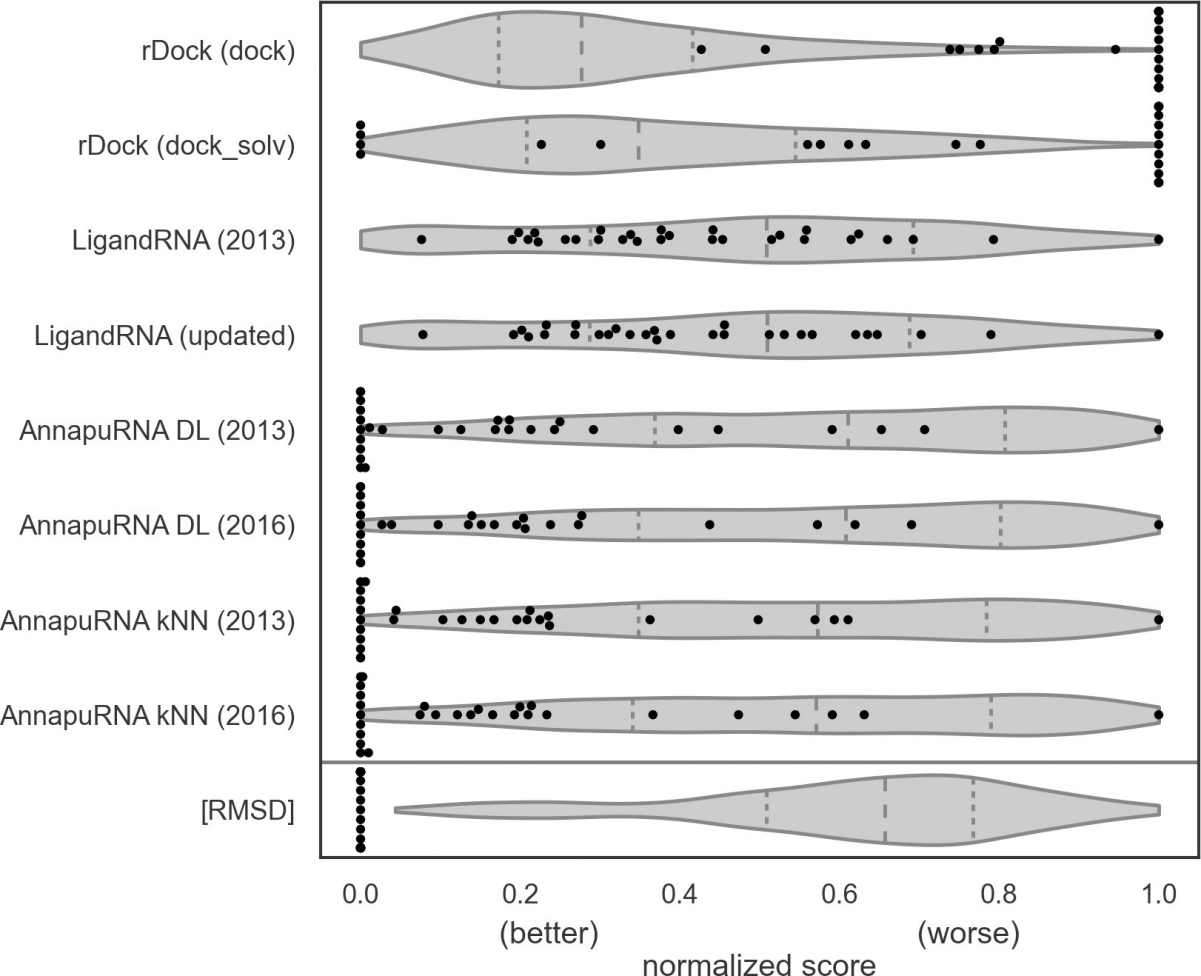
Comparison of scores of the experimentally determined ligand structures (black dots) with the distribution of scores of docked poses (gray violin plots) for the testing set. Additional row [RMSD] represents the distribution of the RMSD values for docked poses and experimentally determined structures. Docking scores and RMSD values were normalized independently for each complex to values [0, 1]. The quartiles of the distribution are marked by the inner vertical dashed lines. Docking was performed using rDock with dock desolvation potential with the native conformation of a ligand as an input.

To analyze the ability of the scoring functions to distinguish between the experimentally determined poses and computationally obtained poses, we rescored the experimentally determined structures from the testing set and compared obtained values with the scores of the docked poses (see Fig 3 and Table 1). Surprisingly, the rDock scoring function values are very high (unfavorable) for the experimentally determined structures in comparison to scores of poses resulting from docking. For the rDock scoring function with dock desolvation potential, only 3.4% of the native structures were in the second quartile of top scored poses resulting from docking. For both variants of rDock, most of the reference structures were ranked with the highest score (i.e., ranked worse than any other poses from docking). For both LigandRNA scoring functions values obtained for the experimentally determined structures are more uniformly distributed among scores of all docked poses, however, the performance was still poor. Only 44.8% of experimentally determined structures were scored by LigandRNA in the first quartile of top scored poses from docking. The performance of AnnapuRNA in ranking the experimentally determined poses was superior to other methods. For all AnnapuRNA variants, 48.3% of experimentally determined complexes were ranked among the top five scored poses resulting from docking. For about one-third of the complexes the experimentally solved structure was ranked as the best among all poses (ranging from 31.0% for AnnapuRNA kNN 2016 to 37.9% for AnnapuRNA DL 2016). Such huge differences in the performance of the scoring functions investigated in this work may result from the nature of the underlying algorithms. While rDock and LigandRNA both utilize all-atom models, AnnapuRNA internally converts RNA and ligand to a coarse-grained representation. In this first case, adding hydrogens to the experimentally determined ligand structures (which is a necessary step prior to scoring, as most of these structures have only coordinates of heavy atoms) may cause unfavorable interactions with RNA, which results in high (unfavorable) scoring values. On the other hand, coarse-grained models of AnnapuRNA don't operate on such a detailed structural level, and thus they are more resistant to small conformational errors, which leads to a better scoring.

**Table 1:** Percentages of the experimentally determined poses determined as the top scoring pose, in the top three, five, 25%, and 50% of scoring poses, among poses resulting from molecular docking. Docking was performed using rDock with the dock desolvation potential with the native conformation of a ligand as an input.

	percentage of complexes, for which the reference ligand is scored:				
	as the top scoring pose	in top three scoring poses	in top five scoring poses	in the first quartile	in the second quartile
rDock (dock)	0.0%	0.0%	0.0%	3.4%	3.4%
rDock (dock_solv)	13.8%	13.8%	17.2%	17.2%	20.7%
LigandRNA (2013)	0.0%	3.4%	6.9%	44.8%	58.6%
LigandRNA (updated)	0.0%	0.0%	3.4%	44.8%	58.6%
AnnapuRNA DL (2013)	34.5%	44.8%	48.3%	72.4%	79.3%
AnnapuRNA DL (2016)	37.9%	41.4%	48.3%	69.0%	79.3%
AnnapuRNA kNN (2013)	34.5%	41.4%	48.3%	72.4%	75.9%
AnnapuRNA kNN (2016)	31.0%	44.8%	48.3%	72.4%	82.8%

### Docking programs with selected scoring functions

**Fig 4:**
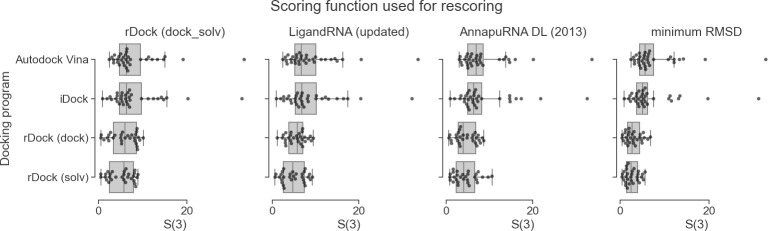
Comparison of the performance of three scoring functions (rDock dock_solv, LigandRNA, and AnnapuRNA) expressed as RMSD to the reference pose of best in top three scored poses (*S*(3)), calculated for four docking programs. The fourth column represents the best (lowest) RMSD obtained during docking for each program. Each dot represents one complex from the testing set. Docking was performed with the native conformation of a ligand as an input.

An important factor to consider in docking is the ability to generate a RNA-ligand pose that resembles the reference structure. To identify the best approach for pose generation, we compared the performance of four molecular docking methods in combination with several scoring functions. For pose generation, we tested rDock (with two desolvation potentials: dock and dock_solv), AutoDock Vina, and iDock. Only rDock is specifically designed for docking ligands to RNA or protein targets, while iDock and Autodock Vina are parameterized only for protein targets. The goal of this analysis was to find the docking program that generates a set of poses with at least one pose having low RMSD to the reference structure, using the ligand conformer taken from the reference structure. Average and median values of the lowest RMSD reported for the above-mentioned four docking programs are reported in Table 2, distribution of RMSD values are depicted in Fig 4, and RMSD values obtained for individual structures are listed in S14 Table.

**Table 2:** Average and median values of the lowest RMSD reported for rDock (dock and dock_solv potentials), AutoDock Vina, and iDock. Values calculated for all structures in the testing set (**A**), only for structures for which all programs completed docking (**B**), and only for structures for which RMSD values of poses found by all programs are below or equal 10 Å (**C**). Docking was performed using a native conformation of a ligand as an input.

	AutodockVina	iDock	rdock-dock	rdock-dock_solv
**A.** All data (33 structures):				
**Average**	**7.1**	**6.5**	**2.6**	**2.3**
**Median**	**5.1**	**4.4**	**2.1**	**1.9**
**B.** Docking completed for all four docking programs (29 structures):				
**Average**	**6.2**	**5.6**	**2.6**	**2.3**
**Median**	**4.8**	**4.2**	**2.1**	**1.9**
**C.** Docking completed for all four docking programs and all RMSD values < 10 Å (25 structures):				
**Average**	**4.4**	**4.0**	**2.4**	**2.2**
**Median**	**4.6**	**4.2**	**1.9**	**1.4**

We found that rDock was unable to generate any solutions for four complexes in our dataset, 2BE0, 2BEE, 2FCZ, and 2PWT. On the other hand, for these structures, AutoDock Vina and iDock successfully generated poses, albeit with high RMSD to the native structure. To avoid bias, apart from calculating the average and median RMSD values for all test cases (Table 2. row A), we computed the performance of the docking programs only for structures for which all programs have successfully completed docking, i.e., generated any pose (Table 2. row B). The best pose generator was found to be rDock with dock_solv desolvation potential. The average minimum RMSD for this method was 2.3 Å, while for the dock potential it was slightly higher—2.6 Å. The RMSD values for Autodock Vina and iDock were remarkably higher—6.2 Å and 5.6 Å, respectively. For two RNA-ligand complexes, Autodock Vina and iDock generated solutions with remarkably poor RMSD (19.1 Å and 32.3 Å, respectively). Such high values can be explained not only by the low performance of the docking algorithm and internal scoring function but also by the multidimensional nature of the RMSD distance and possible flexibility of the defined binding volume. Nevertheless this observation is not surprising—in general, programs designed specifically for RNA-ligand docking generate better RNA-ligand complex models, while the two docking programs, trained for protein-ligand docking, generate conformations more distant to the reference structure. On the other hand, in two cases, AutoDock Vina and iDock were able to generate poses with an RMSD equal to or better than those generated by rDock. These findings imply that in some ‘difficult’ cases, protein-based docking programs may be used to complement the RNA-based methods to generate poses in RNA-ligand docking.

As stated above, in some cases programs generated poses with very high RMSD values. To remove bias caused by these ‘difficult’ complexes, we compared the minimum RMSD values obtained only for structures, for which docking was completed by all four docking programs, and where all RMSD values were lower than or equal to 10 Å (Table 2. row C). Again, the best pose generator was found to be rDock with the dock_solv desolvation potential (average minimum RMSD for this method was 2.2 Å) and rDock with the dock potential (2.4 Å), while RMSD values for Autodock Vina and iDock were higher—4.4 Å and 4.0 Å, respectively.

*S*(3) values for all scoring functions tested are the best for poses generated by rDock. Among all combinations of pose-generating programs with scoring functions, the best performance was achieved for rDock dock_solv in combination with AnnapuRNA scoring functions (see: S15 Table for RMSD values for all complexes in a testing set and S11 Fig for *S*(1) and *S*(5) values distribution).

The analysis of *SR* performance confirms that rDock with dock_solv potential is the best pose-generating program (see: S12 Fig and S16 Table). According to the *SR* scores, the overall best performance is observed for the combination of the rDock dock_solv potential with the AnnapuRNA scoring function, with *SR*(3,2) and *SR*(3,5) equal 0.24 and 0.59 respectively, while the next top-performing method, LigandRNA, reached values 0.10 and 0.55, respectively.

### Conformer generation methods

In the assessment of RNA-ligand pose generators, we use the pose extracted from the experimentally solved structure. However, in real-life cases, the ‘true’ conformation of the ligand is unknown, and it must be predicted for executing the step of pose generation. Hence, we extended our tests to include a comparison of results obtained from docking ligand conformers, which were generated independently from the known bound conformation, using two programs, OpenBabel and Balloon. For each ligand, in each of the programs, we generated a single, lowest-energy conformer and used it for molecular docking.

**Fig 5:**
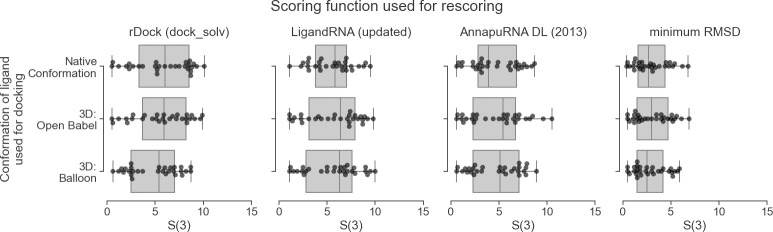
Comparison of the performance of three scoring functions, expressed as a RMSD of the best-scored pose to the reference pose of best in top three scored poses (*S*(3)), calculated for three conformer generation methods. The fourth column represents the best (lowest) RMSD obtained during docking for each program. Each dot represents one complex from the testing set. Docking was performed using rDock with dock desolvation potential.

We found that incorporating the native ligand conformation does not provide a significant advantage in RNA-ligand docking. The differences between the results obtained with the experimentally observed and predicted poses were minimal. The best sets of poses (i.e., the pools of poses containing a pose with the lowest RMSD to the reference structure) were generated when the Balloon program was used as a conformer generator (the average minimal RMSD 2.86 Å), minimally better than the results obtained for the native poses (2.90 Å, see Fig 5). This means that the tested docking programs can sample a conformational space of ligands quite effectively and find the desired RNA-ligand interactions regardless of the starting ligand conformation. This effect is observed regardless of the scoring function used to identify the final solutions (see also S13 Fig *S*(1) and *S*(5) values distribution). This observation is confirmed by the analysis of the *SR* parameter, where the differences between various starting conformers are minimal (see S17 Table and S14 Fig). From this analysis, we conclude that the key elements of the docking pipeline are the choice of the docking program and the scoring function, while the input conformation of a ligand plays a less important role.

Analysis of the statistical significance of our results reveals that although the average and median performance *S*(n) of AnnapuRNA is better than that of other programs, these differences are not always statistically significant. For example, for the median value of the best RMSD for rDock docking program with dock_solv desolvation potential, the AnnapuRNA *k*NN (2013) method is significantly different (better) than all other scoring functions (*p* values ranges from <0.001 for the RF-Score-VS v2, to 0.033 for the updated version of LigandRNA) except the LigandRNA (2013, *p* = 0.053; see Table 3). Tests performed for the same methods, but for the AutoDock docking program show that the AnnapuRNA method is significantly different from rDock (dock_solv) scoring function (*p* = 0.038), while for others functions the difference is not significant (*p* > 0.05).

**Table 3:** *P*-values obtained in a Wilcoxon signed-rank test comparing the distribution of *S*(1), *S*(3), and *S*(5) values of AnnapuRNA and other docking programs. Docking was performed using native conformation of a ligand as an input. Two-tailed *p*-values ≤ 0.05 are in bold. Data referenced in the main text are underlined.

		Wilcoxon signed-rank test *p*-value (two tailed)											
		docking program: rdock (dock_solv)			docking program: rdock (dock)			docking program: AutoDock Vina			docking program: iDock		
AnnapuRNA variant	Compared method	*S*(1)	*S*(3)	*S*(5)	*S*(1)	*S*(3)	*S*(5)	*S*(1)	*S*(3)	*S*(5)	*S*(1)	*S*(3)	*S*(5)
AnnapuRNA DL (2013)	LigandRNA (2013)	0.201	0.601	0.296	0.104	0.095	0.198	0.933	0.939	0.444	0.719	0.964	0.863
	LigandRNA (updated)	0.162	0.455	0.286	0.211	0.088	0.122	1.000	0.977	0.390	0.478	0.964	0.713
	rDock (dock)	**0.009**	**0.042**	**0.036**	**0.032**	**0.024**	**0.044**	0.381	**0.019**	0.206	**0.009**	**< 0.001**	**0.001**
	rDock (dock_solv)	**0.005**	0.057	0.056	**0.016**	**0.014**	**0.027**	0.171	**0.035**	0.087	0.125	0.128	**0.027**
	RF-Score-VS v2	**0.001**	**0.001**	**0.002**	**< 0.001**	**0.001**	**0.002**	0.173	0.117	0.206	0.365	0.094	**0.016**
AnnapuRNA DL (2016)	LigandRNA (2013)	0.465	0.811	0.078	0.819	0.429	0.140	0.540	0.484	0.848	0.339	0.755	0.636
	LigandRNA (updated)	0.313	0.984	0.073	0.475	0.394	0.085	0.597	0.513	0.768	0.194	0.755	0.567
	rDock (dock)	**0.023**	0.136	**0.008**	0.889	0.053	**0.008**	0.799	0.069	**0.033**	0.050	**< 0.001**	**0.002**
	rDock (dock_solv)	**0.014**	0.368	**0.022**	0.647	0.062	**0.009**	0.407	0.079	**0.011**	0.241	0.078	**0.025**
	RF-Score-VS v2	**0.001**	**0.006**	**0.001**	**0.011**	**0.001**	**0.002**	**0.033**	0.264	0.762	0.130	**0.049**	**0.047**
AnnapuRNA kNN (2013)	LigandRNA (2013)	0.053	0.542	0.636	0.200	0.465	0.082	0.459	0.867	0.809	0.468	0.442	0.316
	LigandRNA (updated)	**0.033**	0.469	0.619	0.456	0.404	**0.048**	0.346	0.886	0.809	0.264	0.442	0.183
	rDock (dock)	**0.004**	**0.039**	**0.050**	0.361	0.068	**0.009**	0.107	**0.028**	0.135	0.052	**< 0.001**	**< 0.001**
	rDock (dock_solv)	**0.002**	0.086	0.121	0.264	0.072	**0.008**	**0.038**	**0.039**	0.061	0.144	**0.017**	**0.001**
	RF-Score-VS v2	**<** **0.001**	**0.001**	**0.005**	**0.003**	**0.001**	**0.001**	0.373	0.245	0.334	0.166	0.217	0.472
AnnapuRNA kNN (2016)	LigandRNA (2013)	0.130	0.538	0.363	0.118	0.330	0.217	0.952	0.808	0.438	0.145	0.666	0.690
	LigandRNA (updated)	0.104	0.433	0.349	0.266	0.297	0.131	0.980	0.808	0.386	0.068	0.666	0.556
	rDock (dock)	**0.011**	0.079	**0.044**	0.127	0.182	**0.024**	0.260	**0.021**	0.327	0.108	**0.001**	**0.001**
	rDock (dock_solv)	**0.007**	0.136	0.079	0.097	0.072	**0.028**	0.170	**0.028**	0.199	0.260	0.050	**0.024**
	RF-Score-VS v2	**0.001**	**0.001**	**0.004**	**0.001**	**0.003**	**0.002**	0.098	0.309	0.119	**0.042**	0.153	0.059

For a summary of all combinations of ligand preparation methods, docking programs and scoring functions tested, see S18 Table.

In the current version of AnnapuRNA, we implemented three optional steps for the post-processing of poses from docking or rescoring, namely clustering, centroid calculation, and local optimization. For details, see: S1 Text, chapter “post-processing of docking poses”, S15 Fig, and S19 Table.

### Selected examples from the testing set

**Fig 6:**
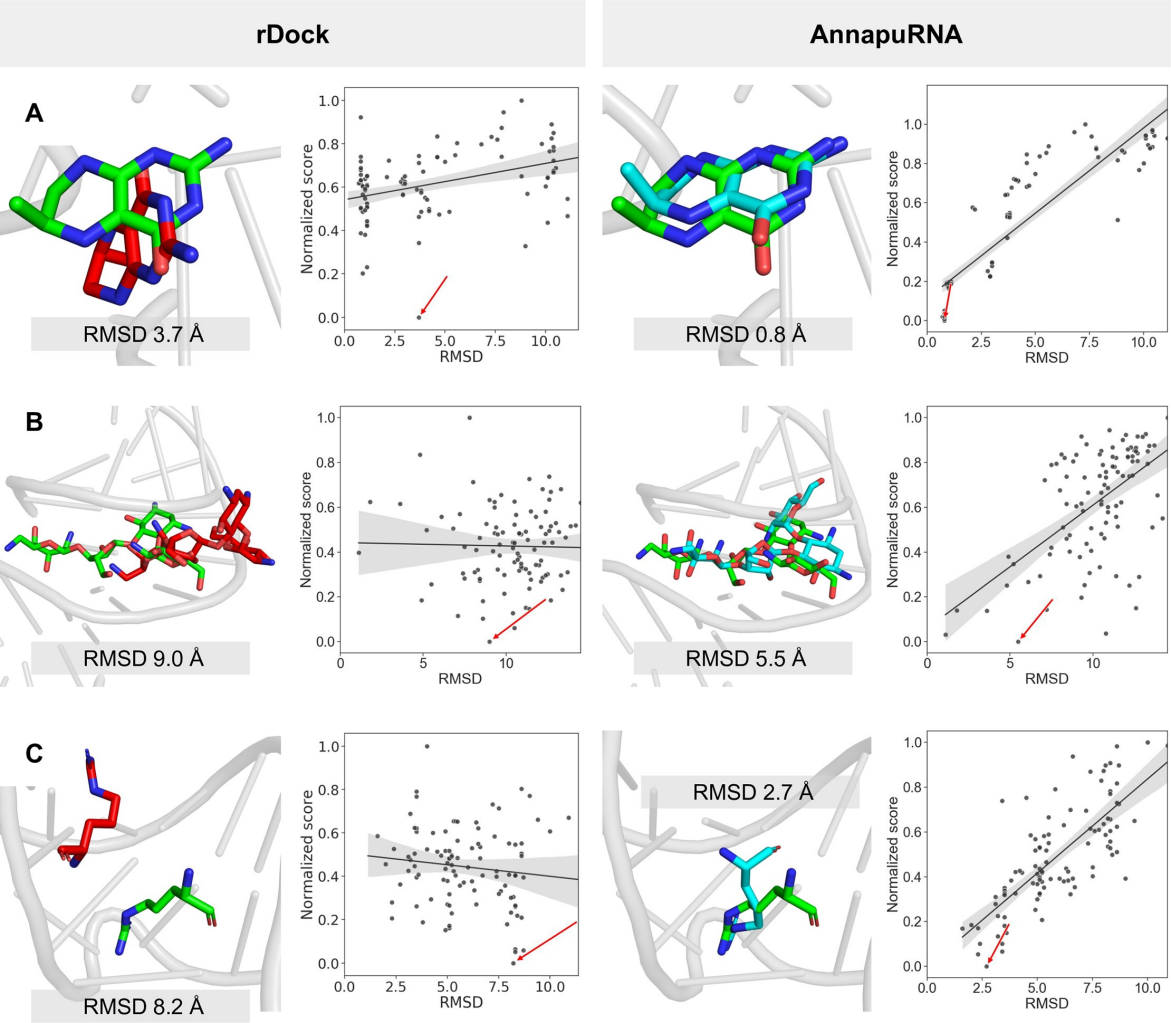
Selected docking solutions for structures from the testing set. **Best pose found by rDock (with dock desolvation potential, left) and AnnapuRNA (DL 2013, right), together with scatter plots of RMSD and normalized score.** Scatter plots show a regression line with a 95% confidence, and the top scoring pose according to each method is indicated by a red arrow. (**A**) 3SUX (Crystal structure of THF riboswitch, bound with THF fragment), (**B**) 1FYP (Decoding region A-site in complex with Paromomycin) and (**C**) 1AJU (HIV-2 TAR-argininamide complex). RNA molecules are presented as a gray cartoon, ligands as sticks; reference structure—green, solution found by rDock—red, solution found by AnnapuRNA—cyan. Heteroatoms are colored: O—red, N—blue.

Fig 6 shows redocking results for three selected cases from the testing set, which compares the AnnapuRNA scoring function (presented in this work and found to outperform other methods) with the runner-up, namely the rDock scoring function. We choose three RNA-ligand complexes with small molecules displaying various degrees of flexibility—rigid, bicyclic THF fragment (Fig 6A), semi-rigid paromomycin, containing four rigid, heterocyclic rings connected by rotatable oxygen linkers (Fig 6B), and a flexible, aliphatic argininamide chain (Fig 6C). We expected that with increased flexibility of the ligand, a more diverse set of conformations (with possibly higher RMSD range) would be generated by the docking program and thus it would be increasingly difficult to determine the near-native pose for the scoring function. In all three cases, the AnnapuRNA scoring function selected the pose closer to the reference one than the rDock scoring function. Also, RMSD vs score plots reveal that energy funnels for AnnapuRNA have a more linear shape, while for rDock the points are more uniformly scattered across the plot. This is an interesting observation, taking into account the fact that RMSD values were not used directly to develop the machine learning models of interactions. This could also mean that the AnnapuRNA score could be correlated with the RMSD and then potentially used for the assessment of RMSD values for a given pose. This hypothesis is supported by the results of analysis of three correlation coefficients (Spearman's rank correlation coefficient, Pearson correlation coefficient, and Kendall rank correlation coefficient) for RMSD-score values. For docking with rDock (in both variants), the mean correlation coefficient is higher (which translates to a better correlation between score and the RMSD) for the AnnapuRNA compared to other scoring functions. For example, the mean Spearman's rank correlation coefficient for rDock (dock), LigandRNA (2013) and AnnapuRNA (*k*NN, 2013) is 0.117, 0.244, and 0.333, respectively. For the updated version of our potential, these values are even higher, reaching values of 0.345 and 0.336 for DL and *k*NN, respectively. This means that the score calculated by AnnapuRNA correlates better with RMSD than the score of other scoring functions tested (for details, see S21 Table and S17 Fig).

### Case study

**Fig 7:**
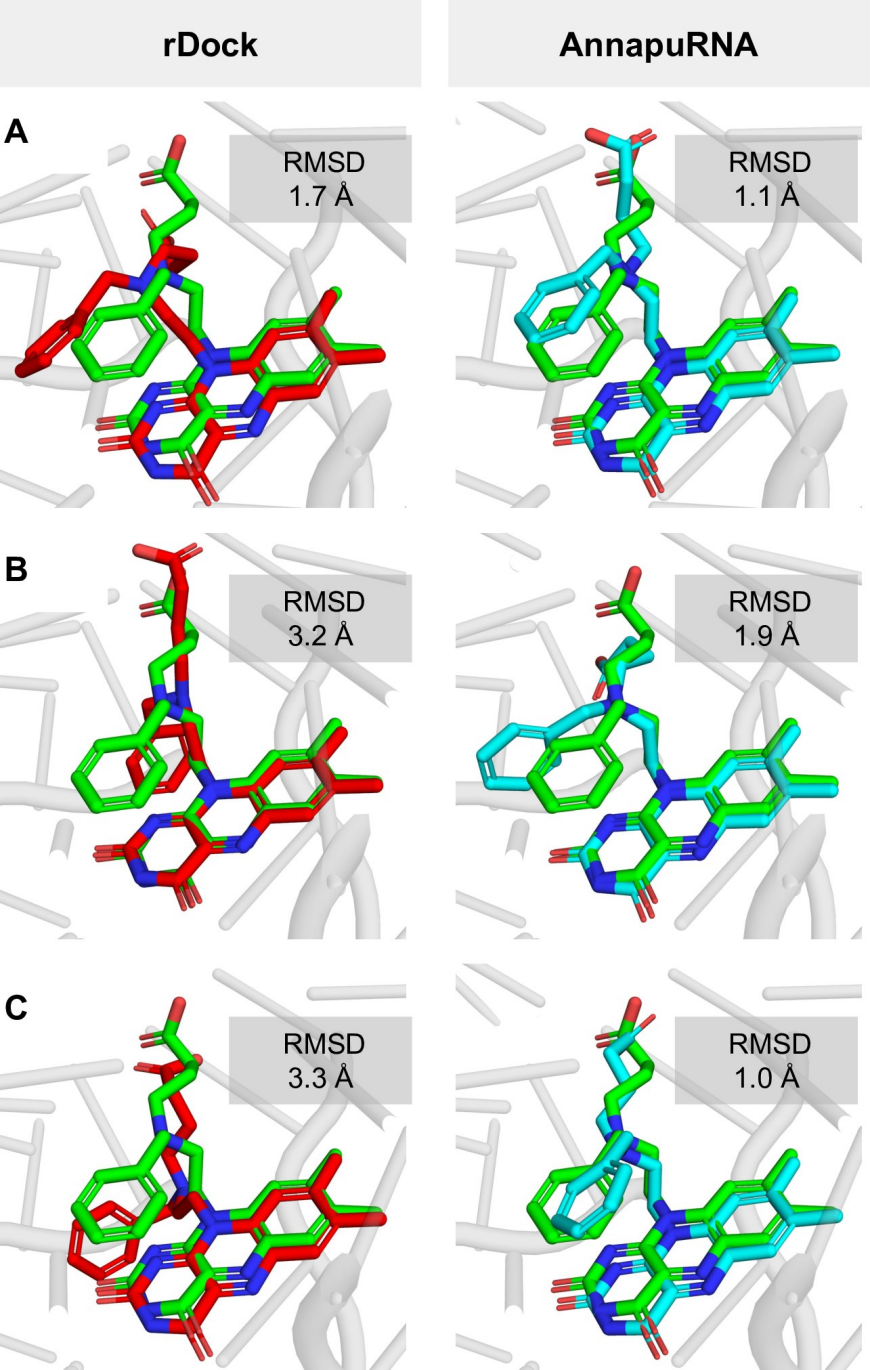
Prediction of the complex of FMN Riboswitch with 4-{benzyl[2-(7,8-dimethyl-2,4-dioxo-3,4-dihydrobenzo[g]pteridin-10(2H)-yl)ethyl]amino}butanoic acid. (**A).** Redocking experiment—ligand extracted from the crystal structure 6DN2 was redocked to this structure; (**B)**. Ligand extracted from the crystal structure 6DN2 docked to FMN structure solved with a different ligand (2YIE); (**C)**. Ligand extracted from the crystal structure 6DN2 docked to the low-resolution APO FMN structure (2YIF). All RNA structures are superimposed. The reference ligands structures are shown in green. Structures predicted by rDock (dock_solv) are shown in magenta (left), predicted by AnnapuRNA (DL 2013) in light blue (right).

One of the most important applications of molecular docking and scoring functions is to predict the binding mode of a small molecule in a binding pocket for a macromolecule of interest—a protein or nucleic acid. In a typical situation, the experimentally solved structure of the macromolecule is used for modeling. In most cases, this structure is available either as a complex with a structurally different ligand or as an apo structure. In these cases, predicting the structure of a new complex is more challenging than in a redocking experiment or when a macromolecule structure solved with a structurally similar ligand is used as a receptor [40]. We performed a set of simulations mimicking this real-life scenario. As a model system, we used a recently published structure of the FMN riboswitch, co-crystallized with a small molecule inhibitor (6DN2, resolution 2.880 Å [41]). Our task was to predict the binding mode of this ligand using two structures of FMN riboswitch described seven years earlier [42]: the apo form (2YIF, resolution 3.298 Å), and a complex with a structurally different ligand (2YIE, resolution 2.941 Å). None of these structures were used for training or testing the AnnapuRNA function. During this test, we compared two scoring functions—rDock and AnnapuRNA (DL 2013). The first experiment involved the redocking of the ligand to the native structure (6DN2). Both scoring functions were able to find a pose that is close to the reference; however, AnnapuRNA performed better than rDock (1.1 Å and 1.7 Å, respectively; Fig 7A).

In the second case study, we used the FMN structure solved with a structurally different ligand. Here, AnnapuRNA outperformed the rDock scoring function (1.9 Å and 3.2 Å, respectively; Fig 7B). As could be expected, the most difficult fragment to predict was an aliphatic chain, which, in the case of a pose selected by AnnapuRNA, is closer to the reference structure than that selected by rDock. Also, in the third experiment, where the apo structure of FMN was used for docking, AnnapuRNA selected a pose that is very close to the reference, in contrast to the rDock (1.0 Å and 3.3 Å, respectively; Fig 7C). To our surprise, in the case of AnnapuRNA, docking to the apo structure (Fig 7C) yielded better results than those obtained from docking to RNA structures originally solved with ligands (Fig 7A and Fig 7B). This can be attributed to a high entropic contribution/flexibility of the aliphatic side chain of the ligand and thus the difficulty of the docking program in searching the full conformational space exhaustively during each docking. Nevertheless, for each of these experiments, AnnapuRNA selected a pose having an RMSD of 1.9 Å to the reference, which can be considered as a successful docking (a most commonly used criterion is 2.0 Å to the reference structure [43]). In this experiment, we showed that a combination of molecular docking and rescoring using the AnnapuRNA function may be a valuable tool for predicting the structures of RNA-ligand complexes, even when no good quality RNA structure is available.

## Conclusion

In summary, we provide a novel method, AnnapuRNA, for predicting interactions of RNA with small-molecule ligands. Our tool can be used as a computational workflow together with a docking program to generate a biologically relevant model of the RNA-ligand complex. Tests reported in this manuscript indicate that our new method is superior to other programs of this kind developed to date. Since AnnapuRNA is a knowledge-based method and depends on the content and quality of databases, its main limitation stems from a relatively low number of experimentally determined RNA-ligand complexes. This number is, however, growing steadily, and we plan to update AnnapuRNA in the future using new RNA-ligand structural data.

Also, conformational changes of RNA are a big challenge for the docking methods, which typically treat the macromolecular receptor as rigid and allow flexibility only for the ligand. If a large structural rearrangement of a ligand-binding site is expected, the typical docking analysis will most likely fail, and if all candidates are completely wrong then AnnapuRNA will not help to select a “good” solution. However, it is worth to emphasize that AnnapuRNA can be used to score and rank models resulting not only from docking methods, but from any method that generates models, such as computational simulations that can be used to address the flexibility of the RNA and structural change upon the ligand binding. Hence, the key issue is to use a method for generating RNA-ligand poses that is fit-for-purpose, i.e., capable of generating poses that are actually close to the real RNA-ligand complex structure. Once this condition is fulfilled, AnnapuRNA is expected to find good solution(s).

## Materials and methods

**Fig 8:**
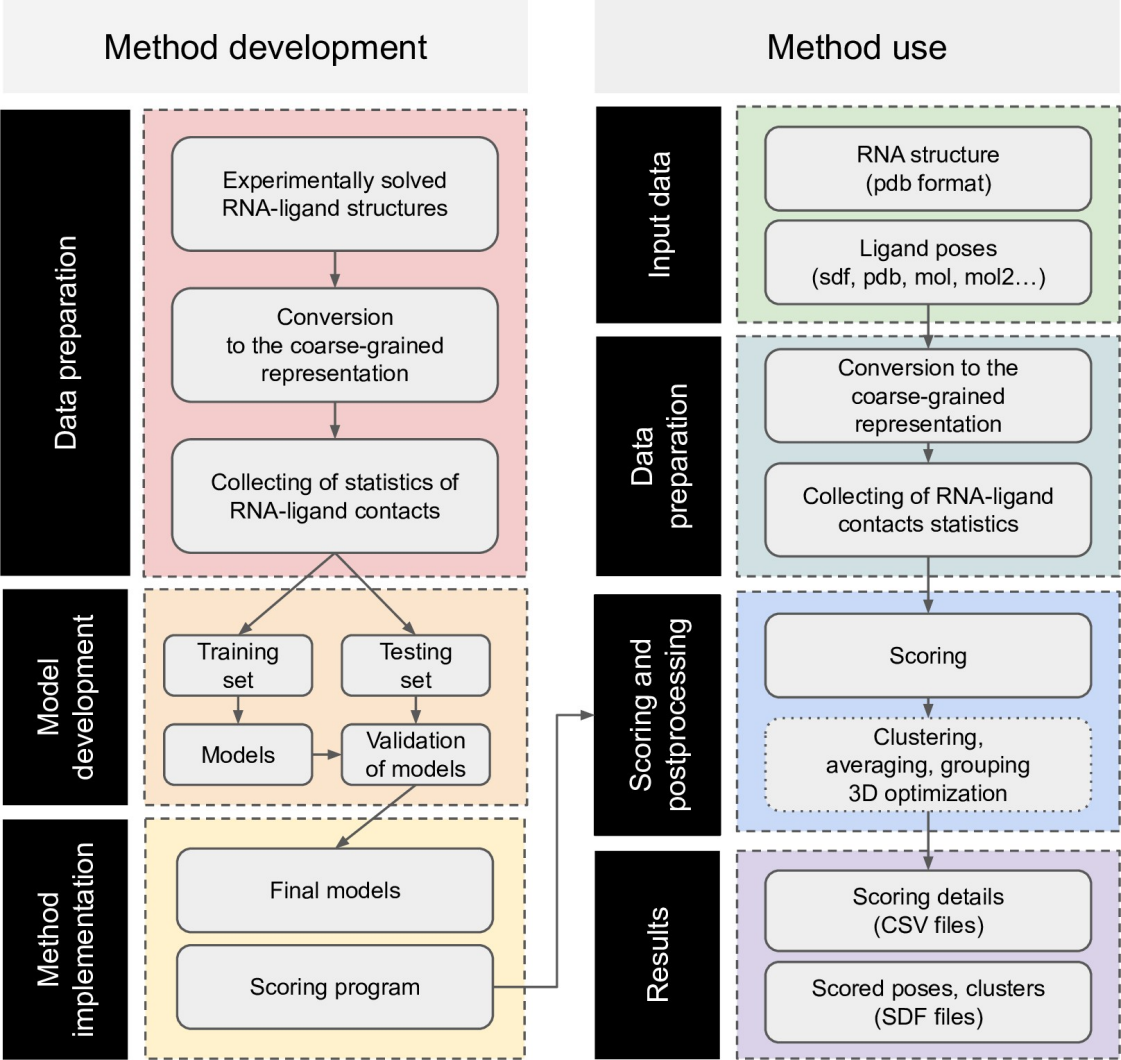
The workflow of AnnapuRNA. **The left column represents the method development pipeline, while the right one displays the method usage.** Optional post-processing steps are placed in boxes with a dotted border.

Fig 8 summarizes the main steps of the AnnapuRNA method development and use.

### Datasets

Two RNA-ligand structures datasets were prepared. One, called “2013” was based on the structures selected by Philips et al. published until 2013 [35]. The second, called “2016” was updated by additional structures solved and published until 2016.08 (extracted from the PDB database with search criteria: contains RNA: yes; has ligand(s): yes; chemical component type: non-polymer) and selected with the criteria described earlier by Philips at al. (i.e., for RNAs with sequence identity >90%, which are in complex with the same ligand, only the structure with the highest resolution was used; for NMR structures the first model in the file was used; for residues with more than one alternative conformation, the first variant was used; structures containing small molecule ligands closer than 6 Å to any atom other than RNA, water or cation were excluded). For the comparison of structures added in the 2016 dataset with those in 2013, see S3 Table and S6 and S7 Figs. Next, for both datasets, RNA structures were preprocessed using the rna-tools software suite (commit b528a29; [44]). Small molecule structures were fetched independently using the RCSB PDB RESTful Web Service interface [45]. Since the AnnapuRNA potential was designed for drug-like compounds, complexes containing ligands with atoms other than C, H, N, O, S, Br, Cl, F, P, Si, B, Se, or lacking C and H atoms were excluded (a molecular formula provided in the xml file fetched from the PDB was used to verify the presence or absence of a given element). To keep only small molecule ligands, we applied the molecular mass criteria with the upper limit of 1000 Da, as defined earlier [46]. This is beyond the most frequently used value of 500 Da [47,48] but allowed us to include larger drug molecules such as oral antibacterial agents, having a substantial number of compounds with the mass in 700–900 Da range [49]. All explicit hydrogens were added to ligand structures with OpenBabel [50], based on the data fetched from the PDB (with no protonation recalculation). For plots showing complexes diversity (pairwise comparison of ligands structural similarity, RNA sequence identity, and RNA RMSD, see S1–S5 Figs and S2 Table).

For each RNA-ligand complex structure, we generated additional near-native poses by redocking the ligand to the RNA receptor. For docking, we used the rDock software, with rigid RNA and flexible ligand, with dock_solv docking protocol and docking radius set to 10. For each complex, 1000 poses were generated and clustered with 2.0 Å RMSD cutoff. We selected a set of poses with RMSD between 0.1 and 1.5 Å to the native ligand pose. The lower value (RMSD < 0.1 Å) was set arbitrarily to exclude poses that are very similar to the native pose. The upper value (RMSD > 1.5 Å) was used earlier to define the threshold of similarity for docking poses [51]. Native and near-native structures were used to derive contact statistics for the “positive” class (native-like solutions). Using the same docking procedure, for each complex, we generated a diversified subset of ligand poses that are different from the native RNA-small molecule structures. For this, we used the RMSD ≥ 4 Å cutoff, which is a doubled value of the commonly used threshold for successful docking [52]. These structures were used to derive contact statistics for the “negative” class (native-unlike solutions).

All ligand structures were converted to a coarse-grained representation with the align-it (version 1.0.3, with noHybrid switch [53]).

### Training and testing set

Both datasets—2013 and 2016—were split into two parts (Table 4): the training set, containing 87 and 131 structures respectively, and testing set, with 33 structures common to both datasets (for the list of structures used, see S1 Table). In both cases, the testing set consisted of the structures earlier used for benchmarking RNA-Ligand scoring functions [35]. The optimization of the scoring function was conducted on the training set, and the testing set was not used until the final benchmark.

**Table 4:** Summary of the 2013 and 2016 datasets.

dataset	Number of complexes—training	Number of complexes—testing
2013	87	33
2016	131	

### Coarse-grained models

To obtain a general model of interactions from the current set of RNA-small molecule complex structures available at the time of the method’s development, we used a coarse-grained representation of both interacting partners. For RNA molecules, we employed a coarse-grained representation used successfully in the SimRNA simulation method, with five ‘beads’ per ribonucleotide residue with positions corresponding to real atoms [54]. In the current version of the scoring function, only the canonical A, G, C, U residues were taken into account. Modified residues (e.g., due to post-transcriptional modification) were not used for developing the potential and were only considered as a steric hindrance. Hence, for docking of ligands to RNA molecules that may have modified residues in the binding sites, we recommend to model the replacement of modified residues with their canonical counterparts, e.g., using the ModeRNA method ([55], also available as a web server, http://iimcb.genesilico.pl/modernaserver/, [56]).

**Fig 9:**
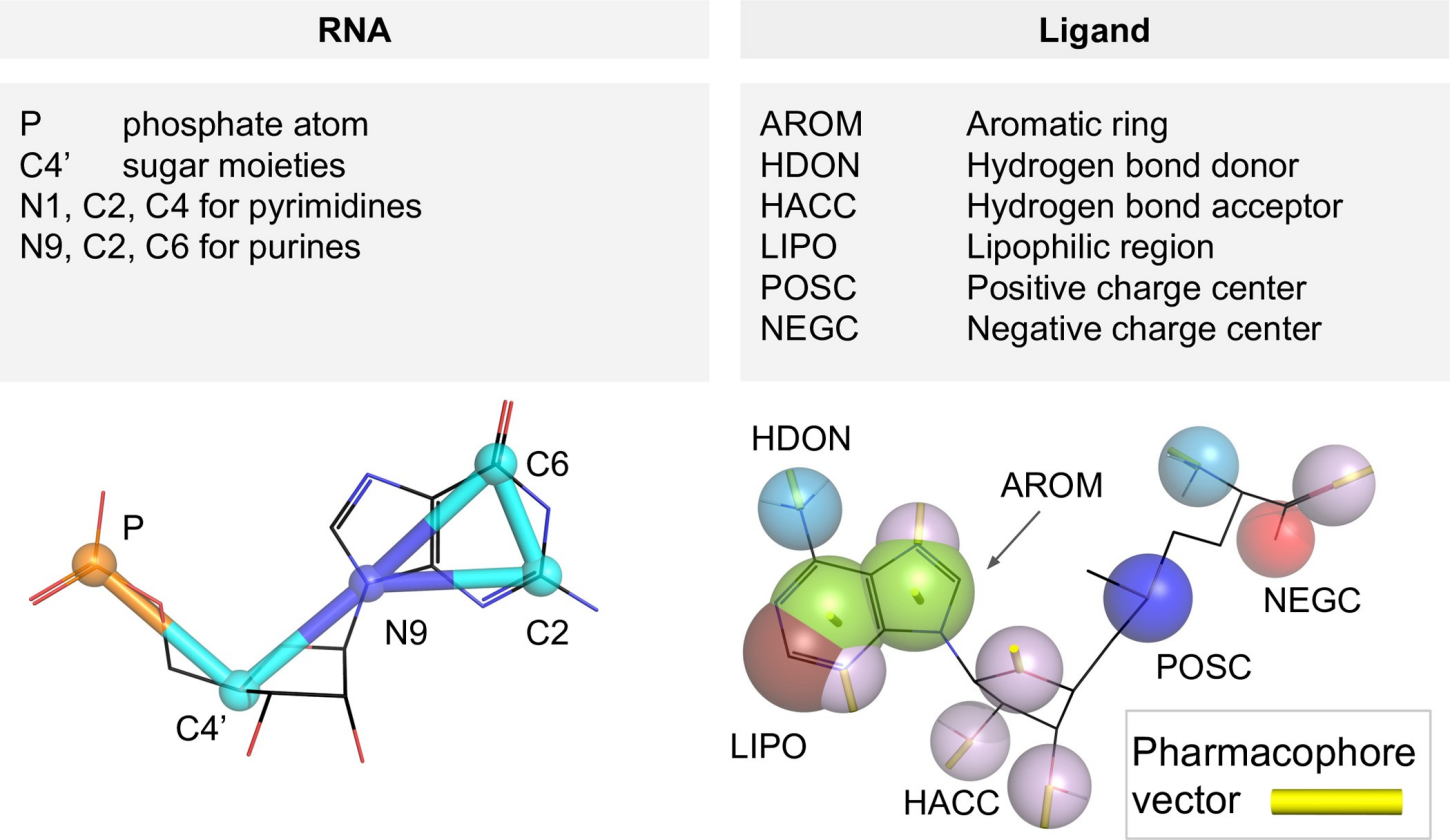
Atoms and pseudoatoms used in the coarse-grained representation of RNA (upper left pane) and ligand molecules (upper right pane), an example of a ribonucleotide (guanosine monophosphate) in SimRNA representation (bottom left) and a small molecule—*S-*adenosylmethionine (bottom right) in the pharmacophore representation.

For small-molecule ligands, we applied the concept of pharmacophores, according to the implementation proposed by Taminau et al. [53]. We used six types of pharmacophores and Euclidean vectors derived for each pseudoatom indicating the direction of the given pharmacophore (See: Fig 9). The centers of the HDON, HACC, POSC, and NEGC points coincide with the position of the heavy atom that is labeled as a hydrogen bond donor, acceptor, carrying a positive or a negative charge. The AROM point is positioned in the center of the aromatic ring it represents. The LIPO point is generated in a multistep procedure, where adjacent lipophilic regions are averaged to a single pharmacophore with respect to their lipophilic contribution. If more than one possible pharmacophore can be assigned to a single chemical group, e.g., hydrogen bond donor and acceptor for an amine or hydroxyl group, both pharmacophore features are assigned independently. Pharmacophore vectors are calculated for pharmacophores for which the direction of the pharmacophore can be defined (namely AROM, HDON, and HACC) and is defined as a normal vector, which originates from the center of the pseudoatom point. The vector indicating the orientation of the ring is located perpendicular to the plane of each ring (for details, see [53]).

### Descriptors of RNA-Ligand interactions

**Fig 10:**
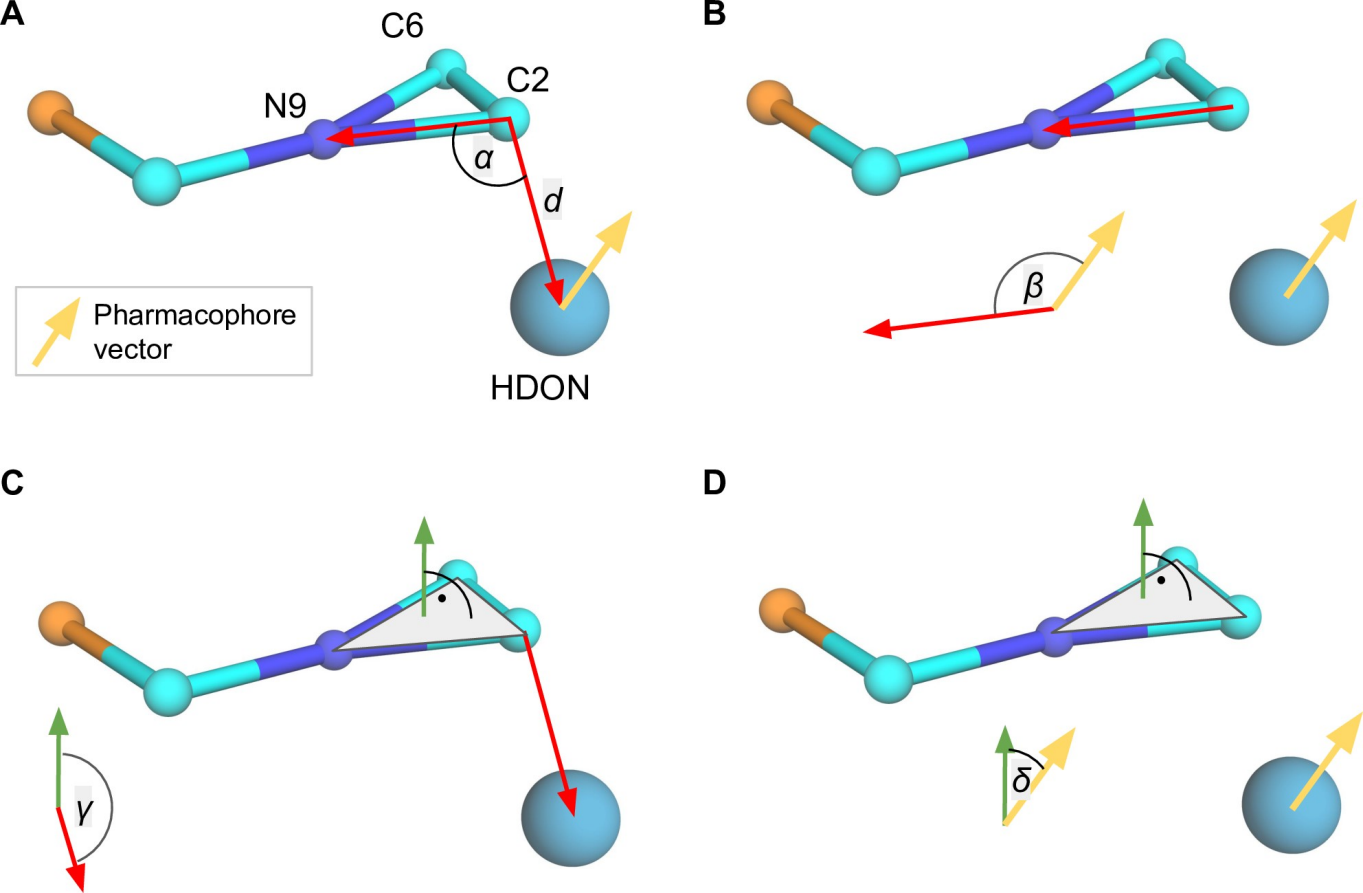
Descriptors collected for RNA-ligands complexes. The distance *d* and the angle *α* between the two selected pseudoatoms of the RNA and the pharmacophore (pane A), the angle *β* between the two selected pseudoatoms of the RNA and the pharmacophore vector (pane B), the angle *γ* between the nucleotide base plane and the pharmacophore (pane C), and the angle *δ* between the base plane and the pharmacophore vector (pane D).

The set of descriptors collected for each RNA-ligand pseudoatom pair consisted of five features that describe geometric relationships between the RNA component and the small molecule component (see: Fig 10): the distance *d*, the angle *α* between the two selected pseudoatoms of the RNA and the pharmacophore (Fig 10A), the angle *β* between the two selected pseudoatoms of the RNA and the pharmacophore vector (Fig 10B), the angle *γ* between the nucleotide base plane and the pharmacophore (Fig 10C), and the angle *δ* between the base plane and the pharmacophore vector (Fig 10D). The nucleotide plane is defined by atoms P, C4', N9 and C2, C6, N9 for purines and P, C4', N1 and C2, C4, N1 for pyrimidines. The interaction threshold was set at 10 Å, a value identified during the optimization of the scoring function (see: chapter “Optimization of parameters of the scoring function” in S1 Text and S6 Table). The total number of data points collected was 65,378,936 for the 2013 dataset and additional 24,198,721 records for the 2016 dataset. The number of data points in the testing set was 26,814,043. Descriptors collected for each contact were assigned a class depending on the origin of the input ligand’s structure—“positive”—for native and native-like solutions or “negative”—for native-unlike solutions.

### Machine learning models of interactions

To derive the scoring models from the collected multidimensional interactions statistics, we used supervised machine learning methods. We tested five widely used algorithms (Deep Learning—multi-layer feedforward artificial neural network—DL, Gaussian Naïve Bayes—GNB, k Nearest Neighbors—*k*NN, Random Forests—RF, and Support Vector Machines with RBF kernel—SVM). After benchmarking these methods, (see: S1 Text, chapter “Optimization of parameters of the scoring function”) we selected two best-performing methods: *k*NN—*k* nearest neighbors method and the Deep Learning (multi-layer feedforward artificial neural network).

*Model implementation details*. Machine learning models for each contact group (base—RNA atom—ligand pseudoatom) were derived independently. To remove contact groups with a statistically insufficient number of contacts, we removed 20 out of 120 possible groups with the number of collected interactions below the arbitrary set threshold of 10 “positive” contacts. For the list of contact groups for which machine learning models were built and used for scoring, see S4 Table).

*Model development and testing*. During the development of machine learning models, we used only the training datasets. We applied a cross-validation scheme with five splits of data according to the ligand structural class. Firstly, ligands were clustered into five groups, using RDKit fingerprint Tanimoto similarity and *k*-Medoids clustering method, representing five chemical classes of compounds: 1) amino acids and carboxylic acids, 2) heterocycles and polycyclic compounds (including nucleobases, nucleotides, and nucleosides), 3) polysugars (polycyclic compounds with relatively high molecular weight), 4) amines (aliphatic compounds not containing carboxylic group), 5) alcohols and polyols (linear compounds with relatively small molecular weight; see S8 Fig for the ligand similarity matrix with marked clusters of ligands). Each variant of AnnapuRNA scoring function was trained using data from four clusters and tested on the data coming from the remaining cluster. This training and testing procedure was repeated for all five combinations of clusters, and the scoring performance was averaged. This cross-validation aimed to minimize a potential bias caused by the overlap of chemical compound types in detected clusters (e.g, alcohols in “polysugars” and “alcohols and polyols “clusters) as well as to ensure that the models trained on data gathered for compounds belonging to four chemical groups can be extended to compounds from a different structural group.

At each cross-validation, split data were class-balanced, i.e., both classes were represented by an equal number of cases.

All descriptors were rescaled to the [0; 1] range. To remove class label noise, for each contact group, we used the Edited Nearest Neighbors algorithm (ENN, [57]) implemented in the imbalanced-learn python package [58]. Parameters of machine learning models were optimized individually for each contact group using Grid Search method (for the hyperparameter search space, see S5 Table). We used two python implementations of machine learning algorithms: scikit-learn (version 0.17, [59]) and H2O (version 3.9.1.3501, https://www.h2o.ai/).

For the optimized set of parameters and developed processing pipeline, we built the final four versions of predictive models—for two selected machine learning algorithms (*k*NN and Deep Learning) using two training datasets (“2013” and “2016”).

### Potential definition

**Fig 11:**
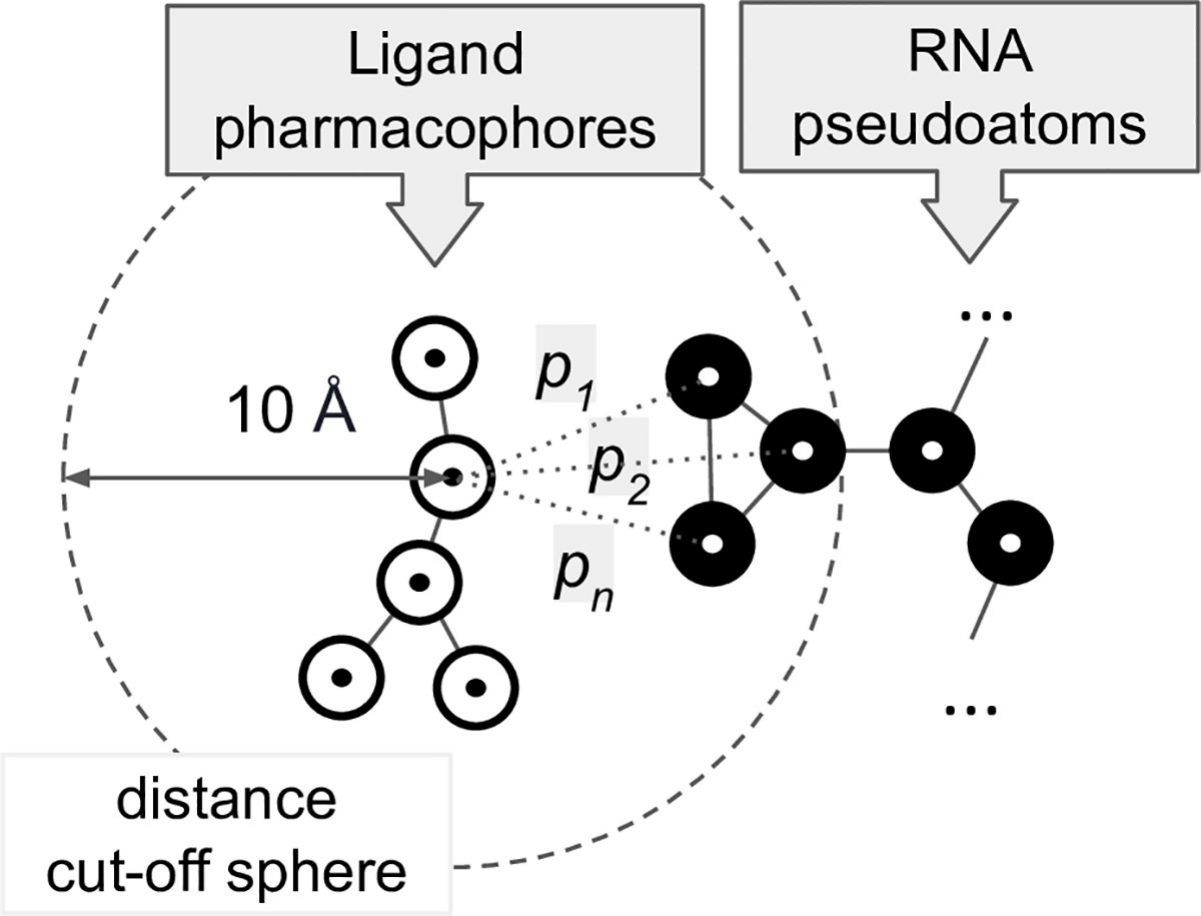
AnnapuRNA scoring function calculates the probabilities *p*_*1*_…*p*_*n*_ of interactions between ligand pharmacophores (white circles) with RNA pseudoatoms (black circles) within the distance of 10 Å. The total score is calculated as a negative sum of all probability values *p* calculated for the given ligand.

The total score for RNA-Ligand complex is a sum of two terms:

$$E=E_{RNA-Ligand}+E_{Ligand}$$ (1)

where *E* is the final score of the complex, *E*_*RNA-Ligand*_ is the score of RNA-Ligand interaction and *E*_*Ligand*_ is a score of the internal ligand’s conformation. The score of RNA-Ligand interaction is expressed as a sum of probabilities *p* of a given interaction for all interactions between RNA atoms and ligand pseudoatoms within the cutoff distance 10 Å (Eq 2 and Fig 11). The probability of RNA-ligand interactions *p* is calculated from a Machine Learning model and it expresses the likelihood that the respective interaction belongs to the “positive” class.

$$E_{RNA-Ligand}=-1\cdot \sum_{interactions}\;p(interaction)$$ (2)

The score of internal energy of ligand, *E*_*Ligand*_, is derived from GAFF internal energy of the ligand [60] and is calculated from:

$$E_{Ligand}=(E_{GAFF}-b)\cdot w$$ (3)

The ligand internal energy contribution is scaled by the factor *b*, which shifts the positive values of the GAFF energy to the negative values. The value of this parameter was set to 473.58, which is equivalent to the third quartile of GAFF energy values calculated for the diversified set of experimentally determined ligand structures deposited in the PDB database. The ligand’s contribution to the final complex score is scaled by the weighting factor *w*. This parameter was set to 0.1 after optimization in a cross-validation experiment but may be changed by the user via a command-line switch.

For the full optimization procedure of parameters of the AnnapuRNA scoring function, see chapter “Optimization of parameters of the scoring function” in S1 Text and S7–S10 Tables.

### Construction of the testing set

To compare the AnnapuRNA scoring function with the previously published methods, we performed a redocking experiment. The testing set consisted of 33 RNA-small molecule complex structures, included in the set used earlier by Philips et al. [35] and for which we were able to obtain docking results using at least two methods.

**Fig 12:**
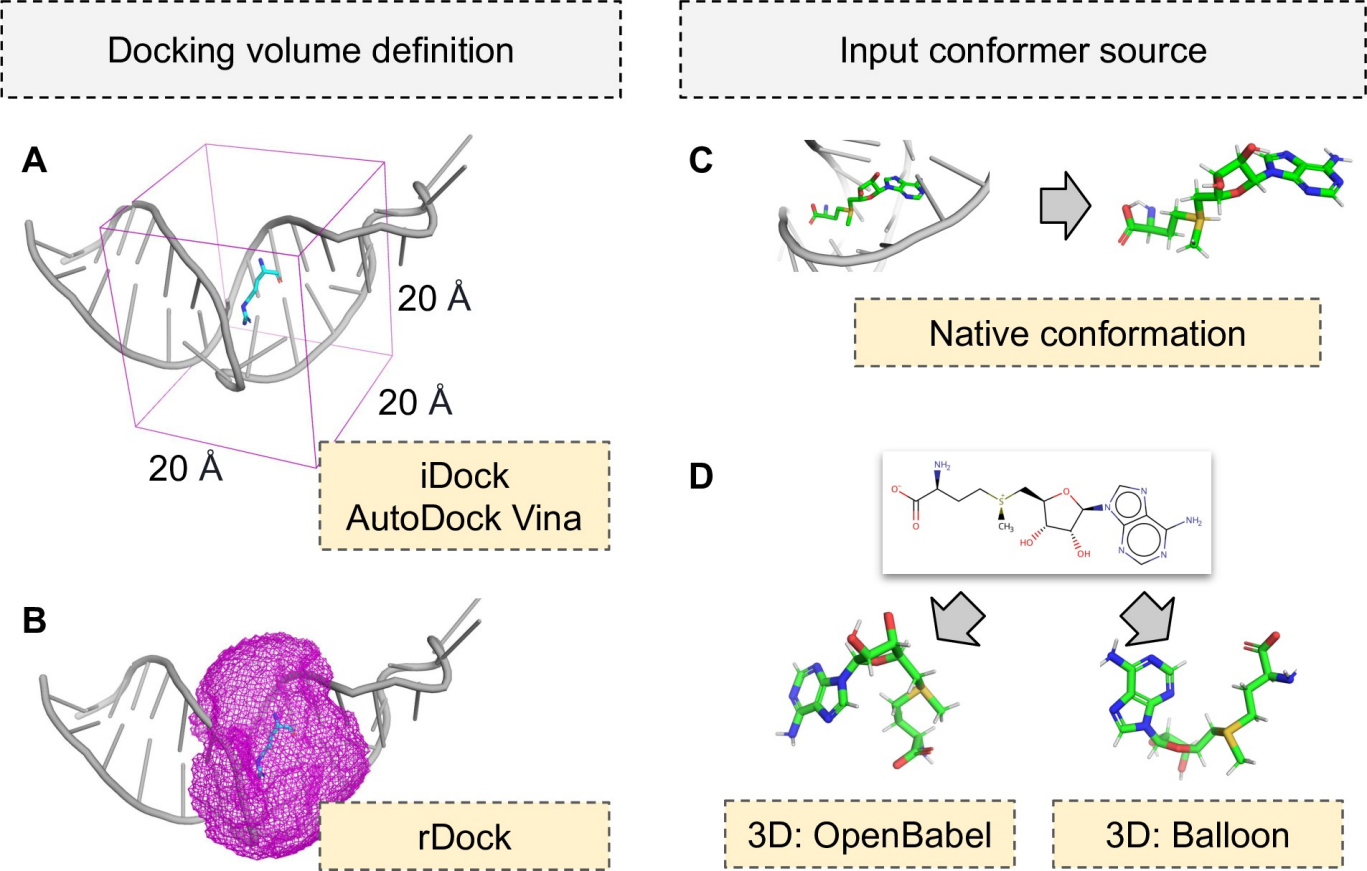
Docking volume definitions for docking programs (left) and input conformer sources used for docking (right). For AutoDock Vina and iDock a box of sizes 20 Å ✕ 20 Å ✕ 20 Å was set, with a center in the center of mass of the ligand (pane A), while for the rDock it is defined as a volume around the known ligand at the maximum distance 10 Å from the ligand atoms (pane B). Three sources of ligand conformers were tested in docking: native pose extracted from the experimentally determined structure (pane C) and conformations generated *de novo* by two programs: OpenBabel and Balloon (pane D).

Many factors may have an influence on docking performance. Among the most obvious ones are the docking program and the scoring function used. Within this work, we tested three docking programs: Autodock Vina [23], iDock (version 2.2.1, [25]) and rDock with two desolvation potentials: dock and dock_solv (version 2013.1, [30]. For each of the docking programs, we specified a docking volume of 10 Å around the ligand (i.e., the grid box size for AutoDock Vina and iDock was set to 20 Å with a center in the center of mass of the ligand—Fig 12A; for rDock, the radius parameter was set to 10 Å—Fig 12B). This procedure determines the volume that is allowed for the docking program to sample in search of the optimal ligand poses. Although the methods used for determining the docking volume differ for those docking programs, the values used in the setup will assure the docking space is similar in all three cases. For each ligand, 100 poses were generated (except AutoDock Vina, for which the maximum number of poses is 20).

Generated poses were rescored using nine scoring functions: LigandRNA (in two versions: the one which is described in the original publication, called “2013”, and the second, with an updated potential, called “updated”), rDock (with two potentials: dock and dock_solv), RF-Score-VS (version v2, [61]) and four variants of the scoring function, AnnapuRNA, presented in this work. The four variants correspond to two versions of the scoring function: *k*NN and DeepLearning, each trained on two datasets: “2013” and “2016”. In the final comparison for iDock and AutoDock Vina, we also included results from the scoring function used by the docking program (termed “Internal SF”).

In the real-life scenario of docking small molecules to a macromolecular receptor, an experimentally solved structure of the macromolecule (Fig 12C) and a *de novo* generated three-dimensional structure of a ligand are used (Fig 12D). In our redocking experiment, we wanted to simulate this process and use not only the native ligand structure extracted from the solved complex but also the single lowest energy ligand conformation generated from one-dimensional SMILES structures. For this purpose we used two widely used tools: OpenBabel with --gen3D option (version 2.3, [50]) and Balloon (version 1.6.2.1236, [62]). OpenBabel uses a multistep procedure, which includes building 3D structures using rules and fragment templates, rough geometry optimization with the MMFF94 force field, weighted rotor conformational search, and the final conjugate gradient geometry optimization. Balloon uses geometry rules to build an initial conformer, which is then subject to geometric modifications encoded by the individuals of the genetic algorithm (modifications include torsion angles, the stereochemistry of double bonds and tetrahedral chiral centers, and ring conformations). The final conformer is relaxed using a modified version of the MMFF94 force field.

### Measuring the docking performance

To test the performance of our method as well as other methods in their ability to identify the most accurate binding poses, we used the *S*(X) metric [35], which reports the lowest RMSD to the reference ligand among the top X scoring poses. Here we used three variants of this metric: *S*(1), *S*(3), and *S*(5), which report the RMSD of the top-scoring pose, the top three poses, and the top five poses, respectively. Additionally, we used the success rate metric *SR*(X,C), which indicates if a given docking analysis was successful or not, according to the criterion that checks if in top X scoring poses there were any poses with RMSD ≤ C Å) and assigns 1 or 0 values, respectively. We analyzed *SR* for the top X = {3, 5} poses with C = {2, 5} Å cut off values, and reported the average values for all complexes in the testing set. As a negative control we used a random selection of docked poses, e.g., for the *SR*(X,C) metric we randomly picked X poses and tested if at least one has RMSD ≤ C Å to the reference structure; this sampling was conducted 100000 times and the *SR* values were averaged.

The RMSD between the docked ligand pose and the reference (experimentally determined) ligand pose was calculated using an in-house python script, and it took into account the symmetry within the ligand structure. We employed the Wilcoxon signed-rank to test if the median scores obtained from the AnnapuRNA algorithm was significantly different (in our case: better) than for other scoring functions and reported two-tailed *p* values. Statistical analysis was performed in the KNIME Analytics Platform 4.1.3 with a Wilcoxon Signed-Rank node [63]. Correlation coefficients were calculated with pandas 1.0.5 [64].

## Supporting information

S1 Fig Matrix of similarity of ligands of all complexes in the 2013 and 2016 training sets, and in the testing set. Complexes included in these datasets are marked with black boxes. Ligand similarity was calculated using the Tanimoto similarity of RDKit fingerprints (the higher the value, the more similar ligands).
(PNG)

S2 Fig Matrix of RNA sequence identity of all complexes in the 2013 and 2016 training sets, and in the testing set. Complexes included in these datasets are marked with orange, green, and blue boxes respectively. Sequence identity was calculated using Clustal 2.1.
(PNG)

S3 Fig Matrix of RNA sequence identity of all complexes in the 2013 and 2016 training sets, and in the testing set. Ligands of the same or very similar structure and RNA sequence identity > = 90% are marked red. Complexes included in these datasets are marked with orange, green, and blue boxes respectively. Sequence identity was calculated using Clustal 2.1.
(PNG)

S4 Fig Matrix of RMSD distance of pairs of RNA of all complexes in the 2013 and 2016 training sets, and in the testing set. Complexes included in these datasets are marked with orange, green, and blue boxes respectively. RMSD was calculated with PyMol 2.5.0 (function align with cycles = 0 parameter).
(PNG)

S5 Fig Matrix of RMSD distance of pairs of RNA of all complexes in the 2013 and 2016 training sets, and in the testing set. Ligands of the same or very similar structure and RNA RMSD < = 5 Å are marked red. Complexes included in these datasets are marked with orange, green, and blue boxes respectively.
(PNG)

S6 Fig Distribution of similarity values of structures from the 2016 dataset to structures in the 2013 dataset: the maximum sequence identity (A), the minimum RMSD distance (B), and the maximum ligand similarity (C).
(PNG)

S7 Fig Bivariate histograms comparing the distribution of similarity values of structures from the 2016 dataset to structures in the 2013 dataset: the maximum sequence identity and the minimum RMSD distance (A), the maximum sequence identity and the maximum ligand similarity (B), and the minimum RMSD distance and the maximum ligand similarity (C).
(PNG)

S8 Fig Tanimoto ligands similarity matrix for the 2013 training set and clusters of ligands used in cross validation. (PNG)

S9 Fig Comparison of the performance of nine scoring functions expressed as the average *SR* values. *SR*(X,C) indicates if a given docking was successful (i.e., in top X scoring poses there was at least one pose with RMSD ≤ C Å). The first row represents the performance obtained when the random poses are selected (the negative control). The last row represents the performance obtained when poses are ranked by the RMSD to the reference structure (positive control). Horizontal lines represent 95% bootstrapped confidence intervals. Docking was performed using rDock with the dock desolvation potential, with the native conformation of a ligand as an input.
(PNG)

S10 Fig Comparison of the performance of nine scoring functions, expressed as a RMSD of the best scored pose to the reference pose (left pane), best in top three scored poses (*S*(3), middle pane), and best in top five scored poses (*S*(5), right pane). Additional rows represent the internal scoring function of the docking program (Internal SF), median, and minimal values of RMSD obtained during the docking. Each dot represents one complex from the testing set. Docking was performed using rDock with the dock_solv desolvation potential (A), iDock (B), and Autodock Vina (C). All docking was performed with the native conformation of a ligand as an input.
(PDF)

S11 Fig Comparison of the performance of three scoring functions—rDock dock_solv, LigandRNA, and AnnapuRNA, expressed as a RMSD to the reference pose of best in scored poses (pane A) and best in top five scored poses (S(5), pane B), calculated for four docking programs. The fourth column represents the best (lowest) RMSD obtained during docking for each program. Each dot represents one complex from the testing set. Docking was performed with the native conformation of a ligand as an input.
(PDF)

S12 Fig Comparison of the performance of three scoring functions (rDock dock_solv, LigandRNA, and AnnapuRNA) expressed as the average *SR* values. *SR*(X,C) indicates if a given docking was successful (*i*.*e*., in top X scoring poses there was at least one pose with RMSD ≤ C Å). The first column represents the performance obtained when the random poses are selected (the negative control). The last column represents the performance obtained when poses are ranked by the RMSD to the reference structure (positive control). Horizontal lines represent 95% bootstrapped confidence intervals. Docking was performed with the native conformation of a ligand as an input.
(PNG)

S13 Fig Comparison of the performance of three scoring functions, expressed as a RMSD to the reference pose of best in scored poses (pane A) and best in top five scored poses (*S*(5), pane B), calculated for three conformer generation methods. The fourth column represents the best (lowest) RMSD obtained during docking for each program. Each dot represents one complex from the testing set. Docking was performed using rDock with the dock desolvation potential.
(PDF)

S14 Fig Comparison of the performance of three scoring functions (rDock dock_solv, LigandRNA, and AnnapuRNA) expressed as the average *SR* values. *SR*(X,C) indicates if a given docking was successful (*i*.*e*., in top X scoring poses there was at least one pose with RMSD ≤ C Å). The first column represents the performance obtained when the random poses are selected (the negative control). The last column represents the performance obtained when poses are ranked by the RMSD to the reference structure (positive control). Horizontal lines represent 95% bootstrapped confidence intervals. Docking was performed using rDock with the dock desolvation potential.
(PNG)

S15 Fig **The best in the three top-scored solutions in redocking experiment (PDB ID: 1KOC) selected by the AnnapuRNA method without post processing (A), with clustering (AutoDock-like’ with 2 Å threshold, best in the three top-scored solutions, pane B), averaging the structure (C), and local optimization of the averaged ligand structure (D).** RNA molecules are presented as a gray cartoon, ligands as sticks, heteroatoms are colored: O—red, N—blue, the reference ligand is green. Docking was performed using rDock with dock desolvation potential with the native conformation of a ligand as an input, and rescored using the AnnapuRNA DL 2016 method.
(TIF)

S16 Fig Docking poses processing time (rDock docking program, 100 poses per complex; time on logarithmic scale) for three scoring functions—LigandRNA developed earlier in our group and the new scoring function in two variants (*k*NN and DL). (PNG)

S17 Fig Correlation coefficient values (RMSD vs score) of nine scoring functions obtained for a testing set, expressed as a Spearman's rank correlation coefficient (left panes), Pearson correlation coefficient (middle panes), and Kendall rank correlation coefficient (right panes). Additional rows represent the internal scoring function of the docking program (Internal SF). Each dot represents one complex from the testing set. Docking was performed using rDock with the dock and dock_solv desolvation potentials (A and B, respectively), iDock (B), and Autodock Vina (C). All docking was performed with native conformation of a ligand as an input.
(PDF)

S1 Table Structures (PDB IDs and ligands IDs) used in the “2013” dataset, the “2016” dataset, and the testing set. For the cases, where PDB database mistakenly separated cocrystallized ligand into several chunks, the ligand was concatenated into a single molecule and ligand ID was indicated as “ligand”. E.g., for complex 1QD3, PDB database indicates four separated ligands (BDG, CYY, IDG, and RIB) instead of a single Neomycin B molecule. ^x^ for complexes from the testing set rDock docking program was not able to perform docking and generate poses.
(PDF)

S2 Table List of pairs of complexes with the same or very similar ligand (fingerprint similarity = 100%), sequence identity > = 90% and RMSD < = 5 Å. (PDF)

S3 Table Comparison of the new structures used in the 2016 dataset with structures in the 2013 dataset. (PDF)

S4 Table List of contact groups (base—RNA atom—ligand pseudoatom) for which machine learning models were built and used for scoring. (PDF)

S5 Table Hyperparameter search space for machine learning methods. (PDF)

S6 Table Performance of scoring functions derived with five machine learning methods, based on four different interaction distance cutoffs. *S*(3) is the averaged value for the cross-validation experiment.
(PDF)

S7 Table Influence on the input data transformation—distance binning and angle binning or cosine transformation and binning, on the performance of scoring functions. S(3) is the averaged value for the cross-validation experiment.
(PDF)

S8 Table Influence on the interaction probability transformations on the performance of scoring functions. *S*(3) is the averaged value for cross-validation experiment.
(PDF)

S9 Table Comparison of two noise removal methods: Edited Nearest Neighbors (ENN) and Tomek’s links (TL) on the performance of two scoring functions, expressed as *S*(3). (PDF)

S10 Table Comparison of the performance of two scoring functions (DL and kNN) for various weights of the ligand term (*w*). (PDF)

S11 Table Summary of the performance, expressed as a RMSD of the best selected pose to the reference pose (*S*(1)), *S*(3), and *S*(5), together with standard deviation values, for nine tested scoring functions. Values represent averages for the testing set. Docking was performed using rDock with the dock desolvation potential, with the native conformation of a ligand as an input.
(PDF)

S12 Table Comparison of the performance of scoring functions for the full testing set and the non-redundant testing set (a full testing set with excluded four structures similar to the training set—the same or very similar ligand, sequence identity > = 90% and RMSD < = 5 Å). Values are highlighted for cases, where the performance is worse for the non-redundant testing set than for the full testing set.
(PDF)

S13 Table SR values for all combinations of conformer generation programs, docking programs, and scoring functions for the training set. (PDF)

S14 Table Lowest RMSD reported for each of the four docking programs. Docking was performed using a native conformation of a ligand as an input. If for a given structure a program was not able to complete the docking successfully, the row was grayed out. If for a given structure a docking program was not able to find a solution with RMSD ≤ 10 Å, the row was marked red. The bottom rows summarizes the performance for all data presented (A), only for a set of structures, for which all programs completed docking (B), and only for a set of structures, for which RMSD values of poses found by all programs were below or equal to 10 Å (C).
(PDF)

S15 Table Comparison of scoring performance for a combination of four docking methods, with ten scoring functions. Additional column ([RMSD]) shows the lowest RMSD poses obtained during docking. Docking was performed with the native conformation of a ligand as an input.
(PDF)

S16 Table Comparison of the performance of three scoring functions (rDock dock_solv, LigandRNA, and AnnapuRNA) expressed as the average *SR* values. *SR*(X,C) indicates if a given docking was successful (*i*.*e*., in top X scoring poses there was at least one pose with RMSD ≤ C Å). The first column represents the performance obtained when the random poses are selected (the negative control). The last column represents the performance obtained when poses are ranked by the RMSD to the reference structure (positive control). Docking was performed with the native conformation of a ligand as an input.
(PDF)

S17 Table Comparison of the performance of three scoring functions (rDock dock_solv, LigandRNA, and AnnapuRNA) expressed as the average *SR* values. *SR*(X,C) indicates if a given docking was successful (*i*.*e*., in top X scoring poses there was at least one pose with RMSD ≤ C Å). The first column represents the performance obtained when the random poses are selected (the negative control). The last column represents the performance obtained when poses are ranked by the RMSD to the reference structure (positive control). Docking was performed using rDock with the dock desolvation potential.
(PDF)

S18 Table Comparison of scoring performance for a combination of three methods of conformer generation, with four docking programs, with ten scoring functions. Additional column ([RMSD]) shows the lowest RMSD poses obtained during the docking.
(PDF)

S19 Table Comparison of the performance of AnnapuRNA scoring functions for the ‘AutoDock-like’ clustering (100% of poses and 2 Å RMSD threshold). Docking was performed using rDock with the dock desolvation potential with the native conformation of a ligand as an input.
(PDF)

S20 Table Docking poses processing time (rDock docking program, 100 poses per complex) for three scoring functions—LigandRNA developed earlier in our group and the new scoring function in two variants (*k*NN and DL). (PDF)

S21 Table Mean and median correlation coefficient values (RMSD vs. score values) obtained for a testing set of nine scoring functions, expressed as a Spearman's rank correlation coefficient, Pearson correlation coefficient, and Kendall rank correlation coefficient. Additional rows represent the internal scoring function of the docking program (Internal SF). All docking was performed with native conformation of a ligand as an input.
(PDF)

S1 Text Additional information. Optimization of parameters of the scoring function; post-processing of docking poses; additional information on docking algorithms.
(PDF)
