# Supplementary figures and images for: AnnapuRNA: A scoring function for predicting RNA-small molecule binding poses
Source: PLoS Comput Biol. 2021 Feb 1;17(2):e1008309. doi: 10.1371/journal.pcbi.1008309 (PMC7877745; doi:10.1371/journal.pcbi.1008309)

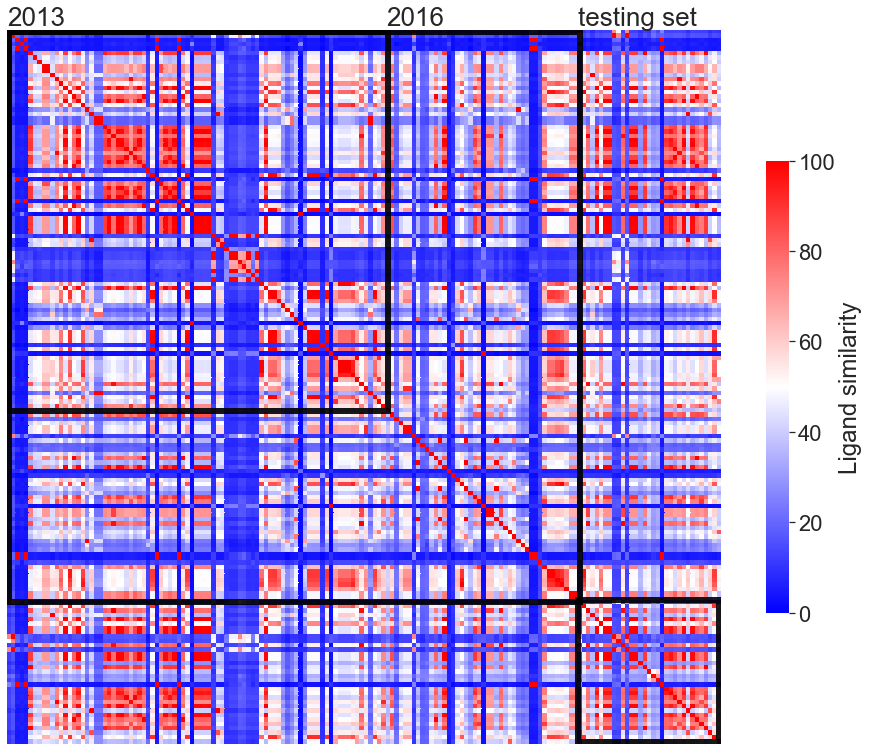

Supplement: S1 Fig — Complexes included in these datasets are marked with black boxes. Ligand similarity was calculated using the Tanimoto similarity of RDKit fingerprints (the higher the value, the more similar ligands). (PNG) [file pcbi.1008309.s001.png]

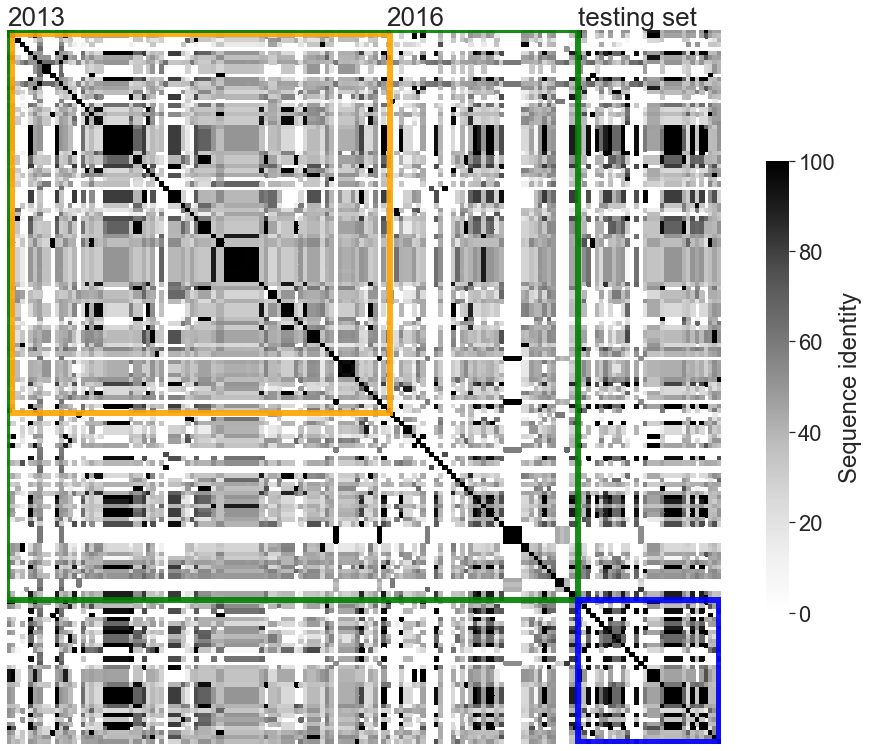

Supplement: S2 Fig — Complexes included in these datasets are marked with orange, green, and blue boxes respectively. Sequence identity was calculated using Clustal 2.1. (PNG) [file pcbi.1008309.s002.png]

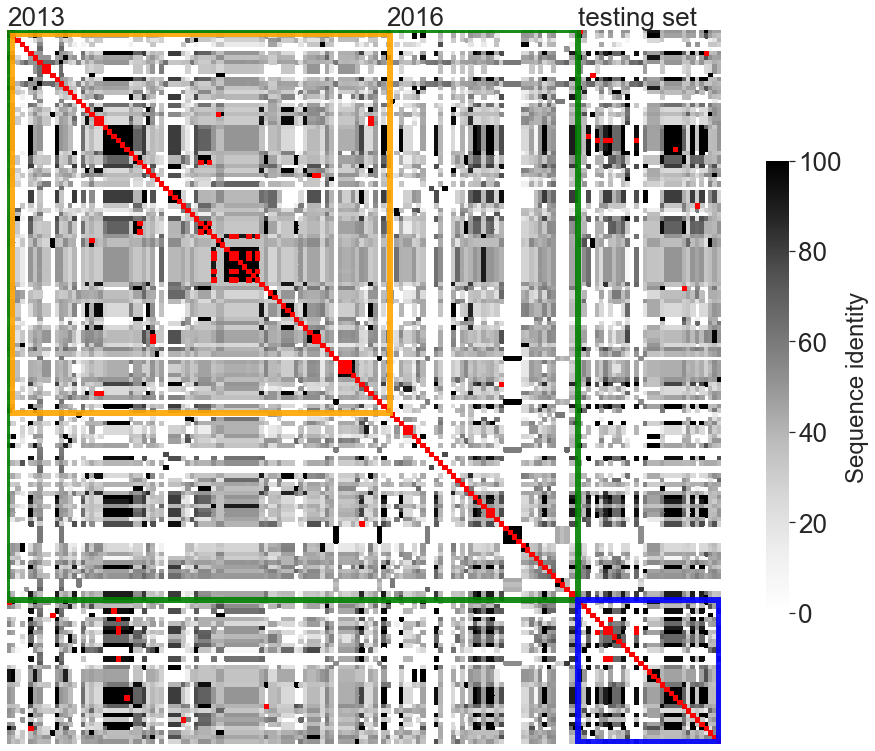

Supplement: S3 Fig — Ligands of the same or very similar structure and RNA sequence identity > = 90% are marked red. Complexes included in these datasets are marked with orange, green, and blue boxes respectively. Sequence identity was calculated using Clustal 2.1. (PNG) [file pcbi.1008309.s003.png]

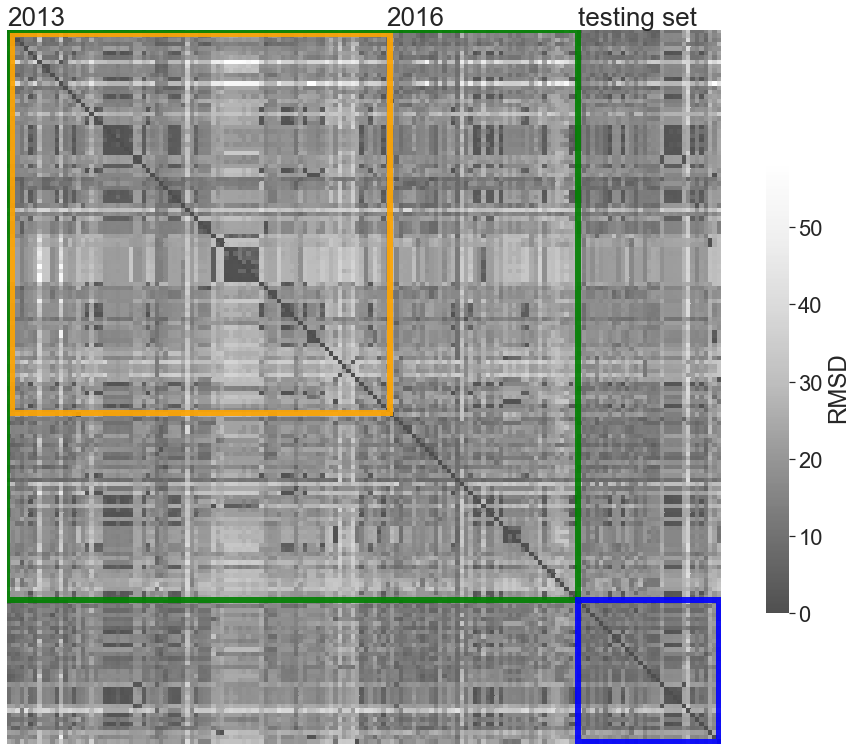

Supplement: S4 Fig — Complexes included in these datasets are marked with orange, green, and blue boxes respectively. RMSD was calculated with PyMol 2.5.0 (function align with cycles = 0 parameter). (PNG) [file pcbi.1008309.s004.png]

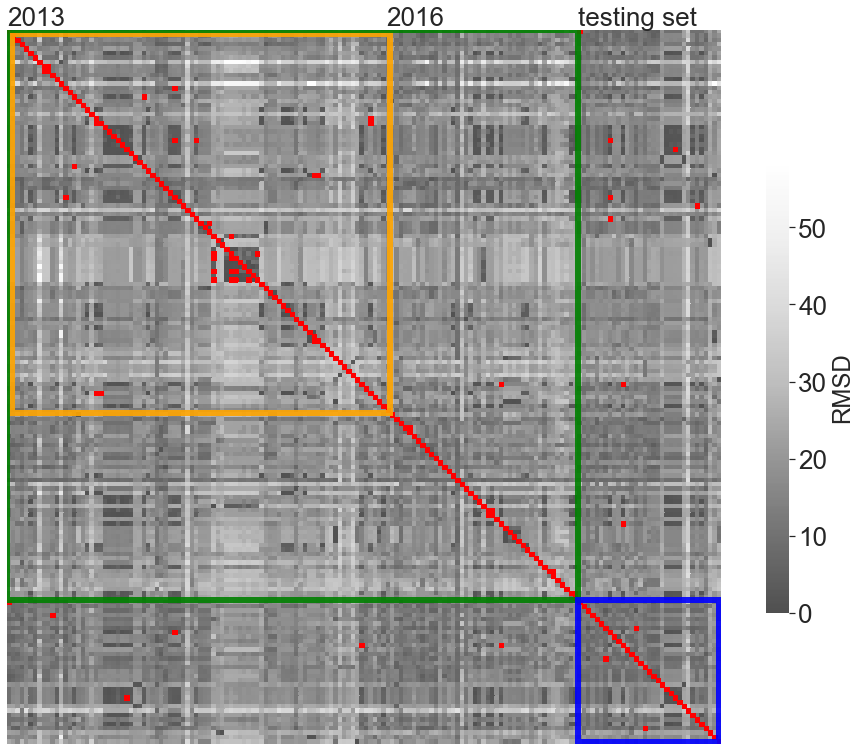

Supplement: S5 Fig — Ligands of the same or very similar structure and RNA RMSD < = 5 Å are marked red. Complexes included in these datasets are marked with orange, green, and blue boxes respectively. (PNG) [file pcbi.1008309.s005.png]

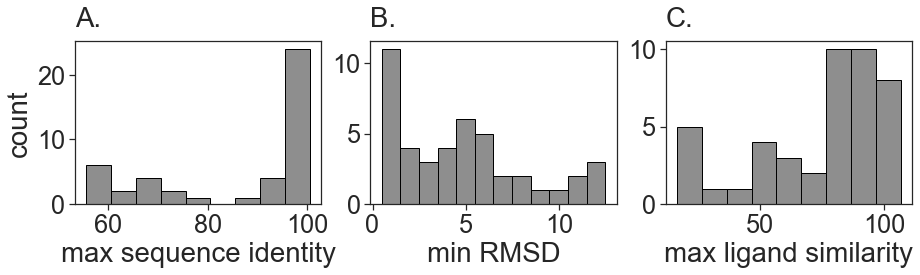

Supplement: S6 Fig — Distribution of similarity values of structures from the 2016 dataset to structures in the 2013 dataset: the maximum sequence identity (A), the minimum RMSD distance (B), and the maximum ligand similarity (C). (PNG) [file pcbi.1008309.s006.png]

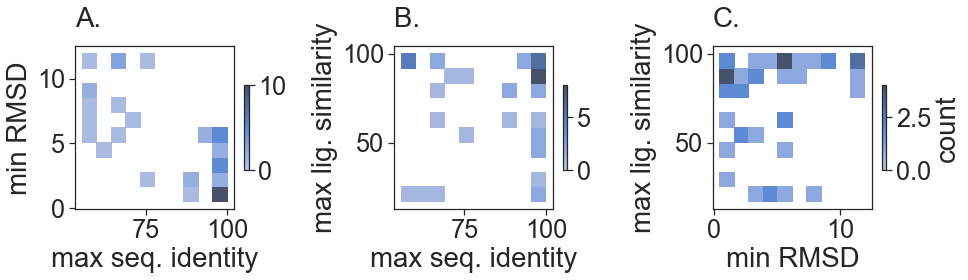

Supplement: S7 Fig — Bivariate histograms comparing the distribution of similarity values of structures from the 2016 dataset to structures in the 2013 dataset: the maximum sequence identity and the minimum RMSD distance (A), the maximum sequence identity and the maximum ligand similarity (B), and the minimum RMSD distance and the maximum ligand similarity (C). (PNG) [file pcbi.1008309.s007.png]

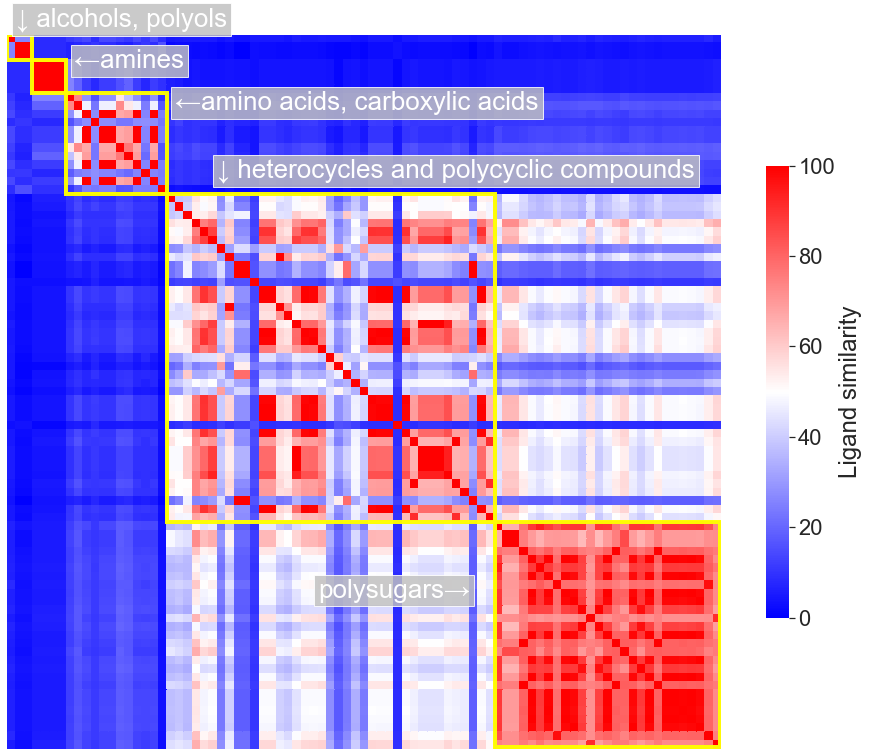

Supplement: S8 Fig — (PNG) [file pcbi.1008309.s008.png]

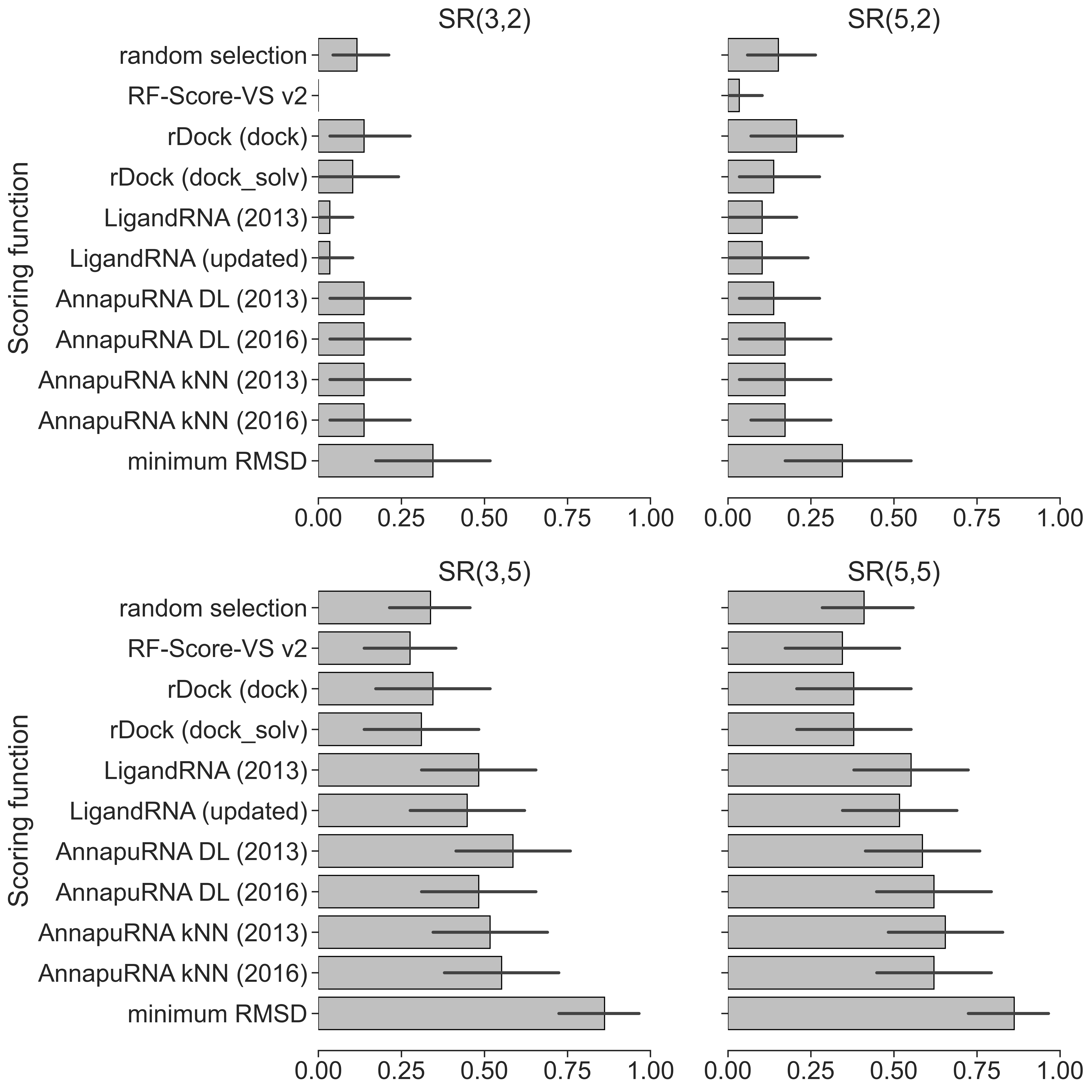

Supplement: S9 Fig — SR(X,C) indicates if a given docking was successful (i.e., in top X scoring poses there was at least one pose with RMSD ≤ C Å). The first row represents the performance obtained when the random poses are selected (the negative control). The last row represents the performance obtained when poses are ranked by the RMSD to the reference structure (positive control). Horizontal lines represent 95% bootstrapped confidence intervals. Docking was performed using rDock with the dock desolvation potential, with the native conformation of a ligand as an input. (PNG) [file pcbi.1008309.s009.png]

A.

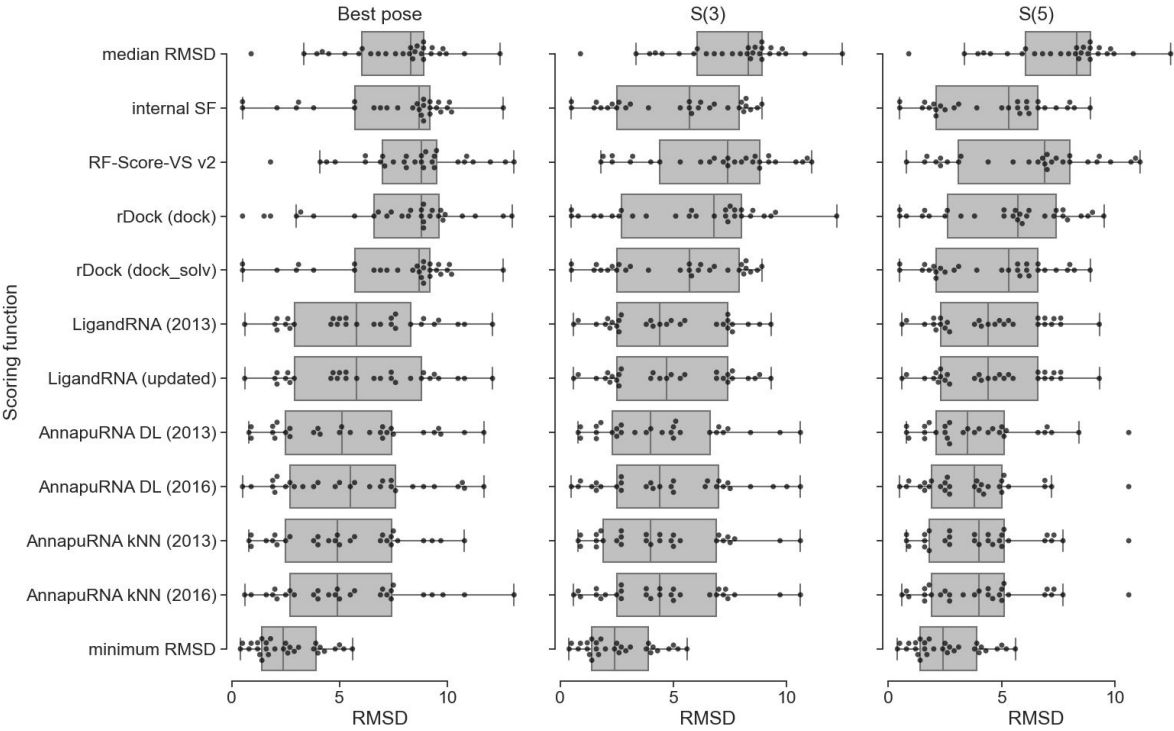

B

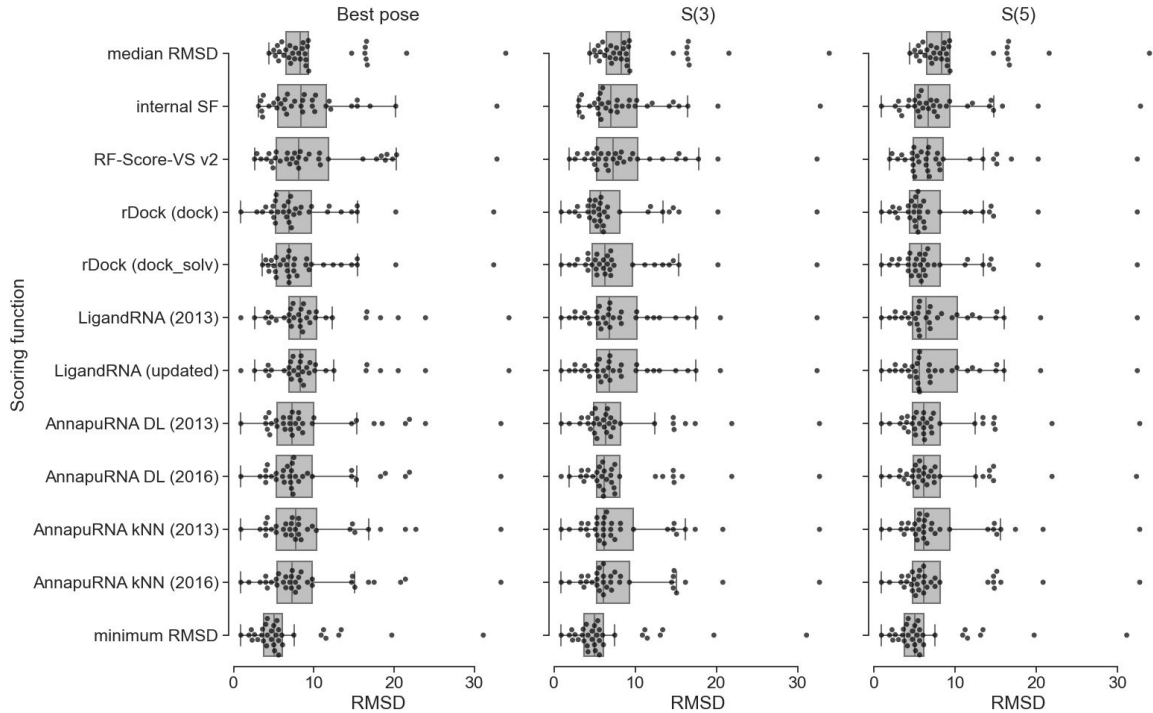

C.

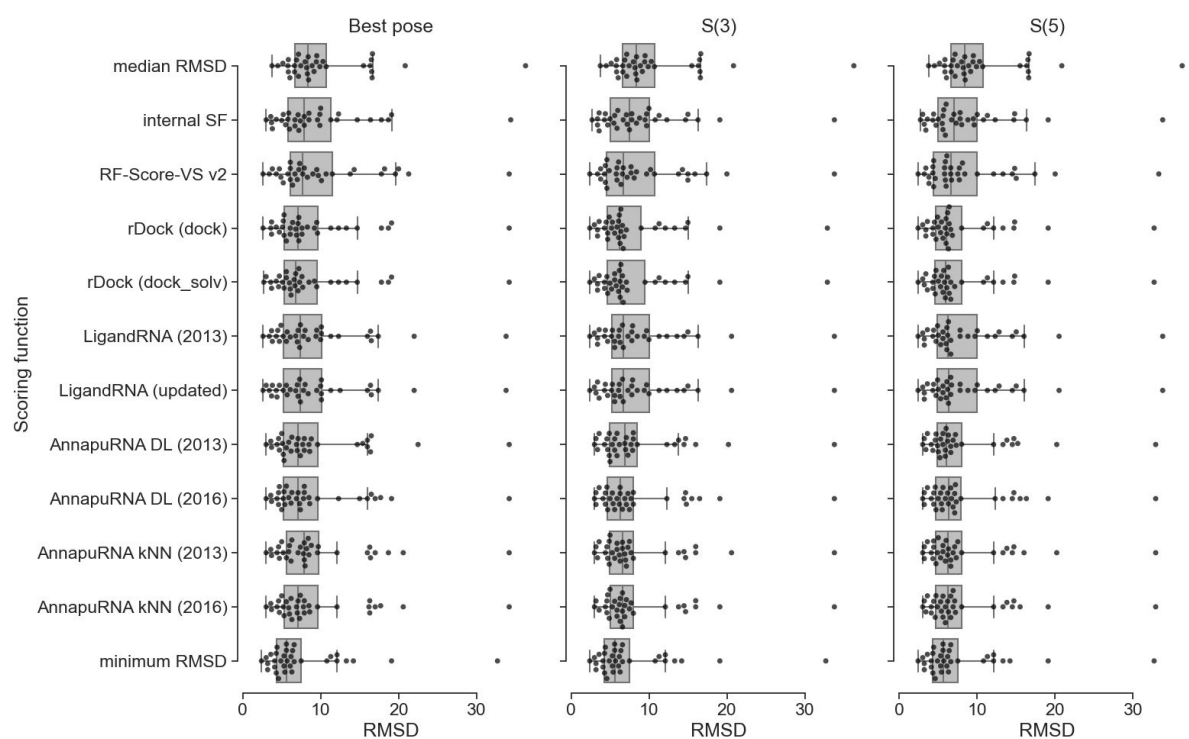

Supplement: S10 Fig — Additional rows represent the internal scoring function of the docking program (Internal SF), median, and minimal values of RMSD obtained during the docking. Each dot represents one complex from the testing set. Docking was performed using rDock with the dock_solv desolvation potential (A), iDock (B), and Autodock Vina (C). All docking was performed with the native conformation of a ligand as an input. (PDF) [file pcbi.1008309.s010.pdf]

A.

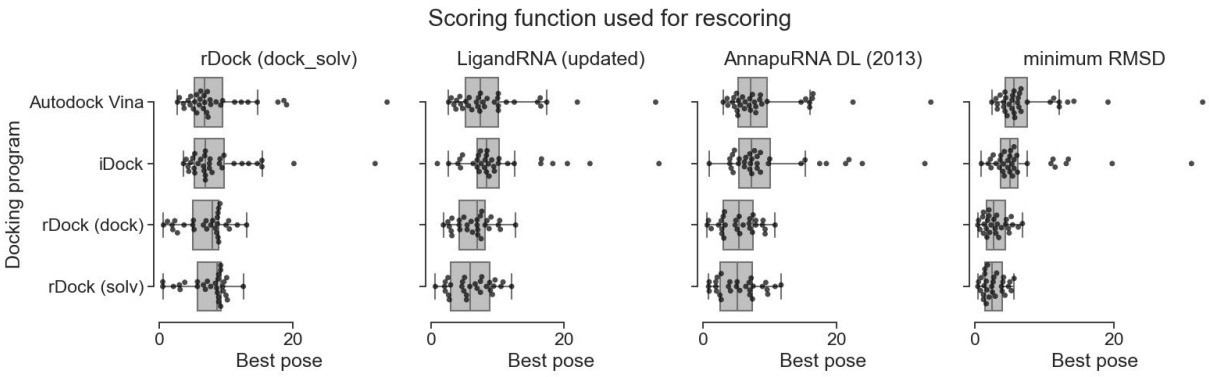

B.

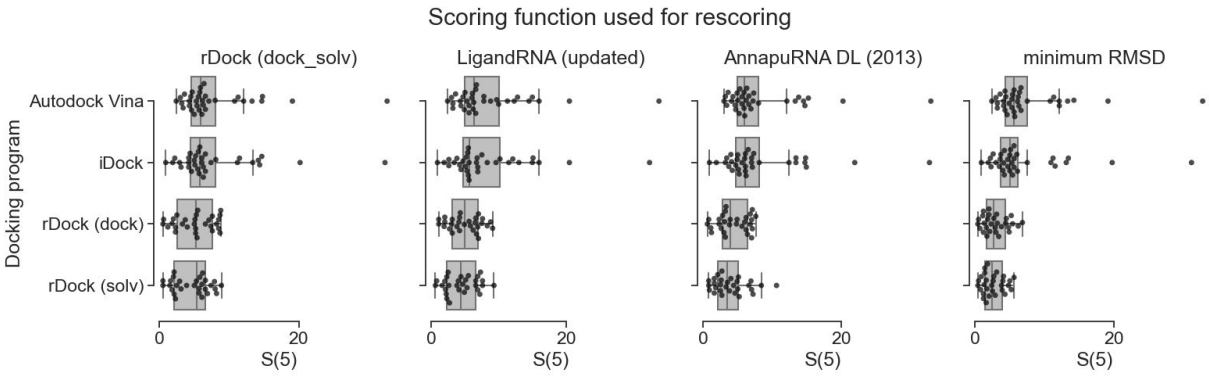

Supplement: S11 Fig — Comparison of the performance of three scoring functions—rDock dock_solv, LigandRNA, and AnnapuRNA, expressed as a RMSD to the reference pose of best in scored poses (pane A) and best in top five scored poses (S(5), pane B), calculated for four docking programs. The fourth column represents the best (lowest) RMSD obtained during docking for each program. Each dot represents one complex from the testing set. Docking was performed with the native conformation of a ligand as an input. (PDF) [file pcbi.1008309.s011.pdf]

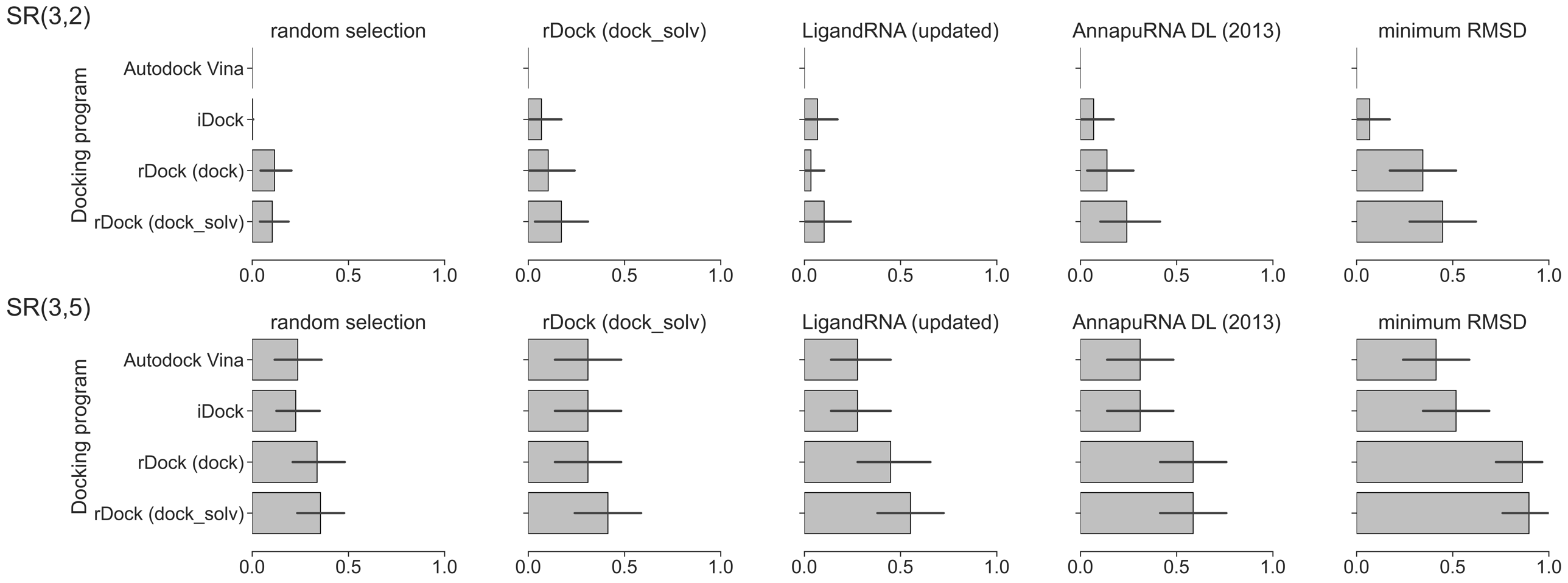

Supplement: S12 Fig — SR(X,C) indicates if a given docking was successful (i.e., in top X scoring poses there was at least one pose with RMSD ≤ C Å). The first column represents the performance obtained when the random poses are selected (the negative control). The last column represents the performance obtained when poses are ranked by the RMSD to the reference structure (positive control). Horizontal lines represent 95% bootstrapped confidence intervals. Docking was performed with the native conformation of a ligand as an input. (PNG) [file pcbi.1008309.s012.png]

A.

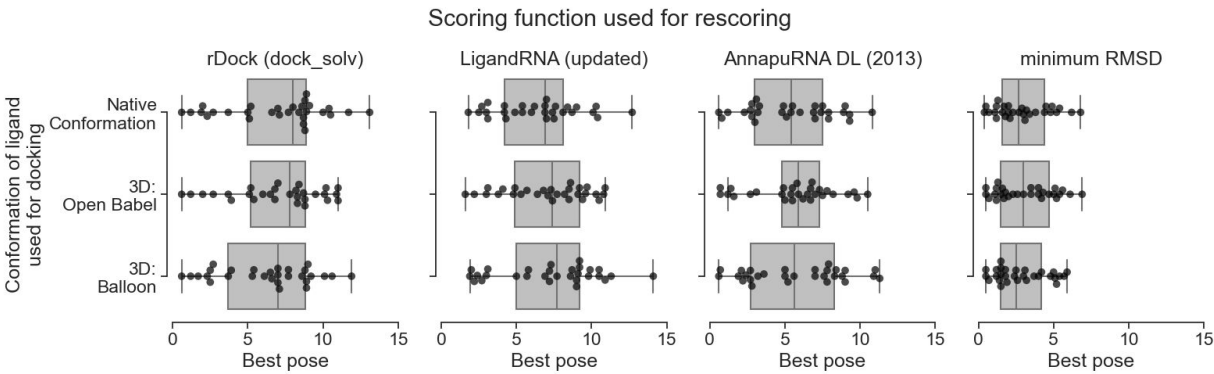

B.

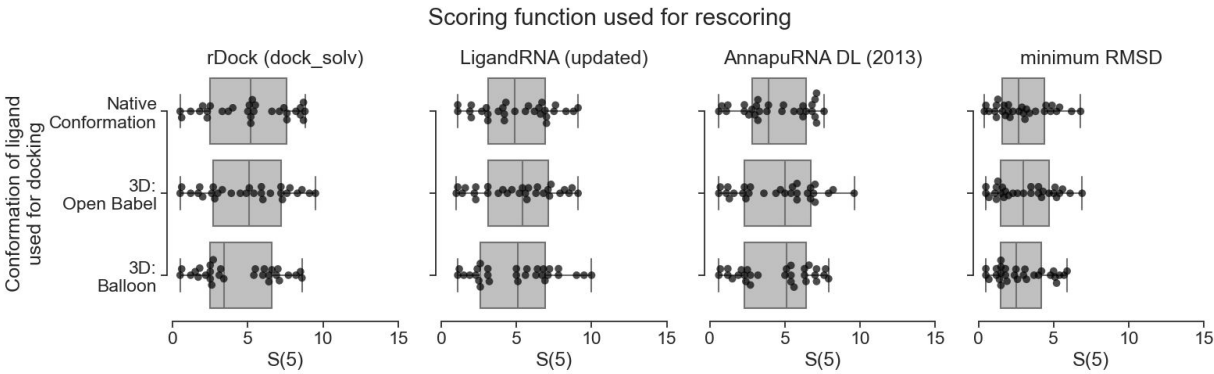

Supplement: S13 Fig — The fourth column represents the best (lowest) RMSD obtained during docking for each program. Each dot represents one complex from the testing set. Docking was performed using rDock with the dock desolvation potential. (PDF) [file pcbi.1008309.s013.pdf]

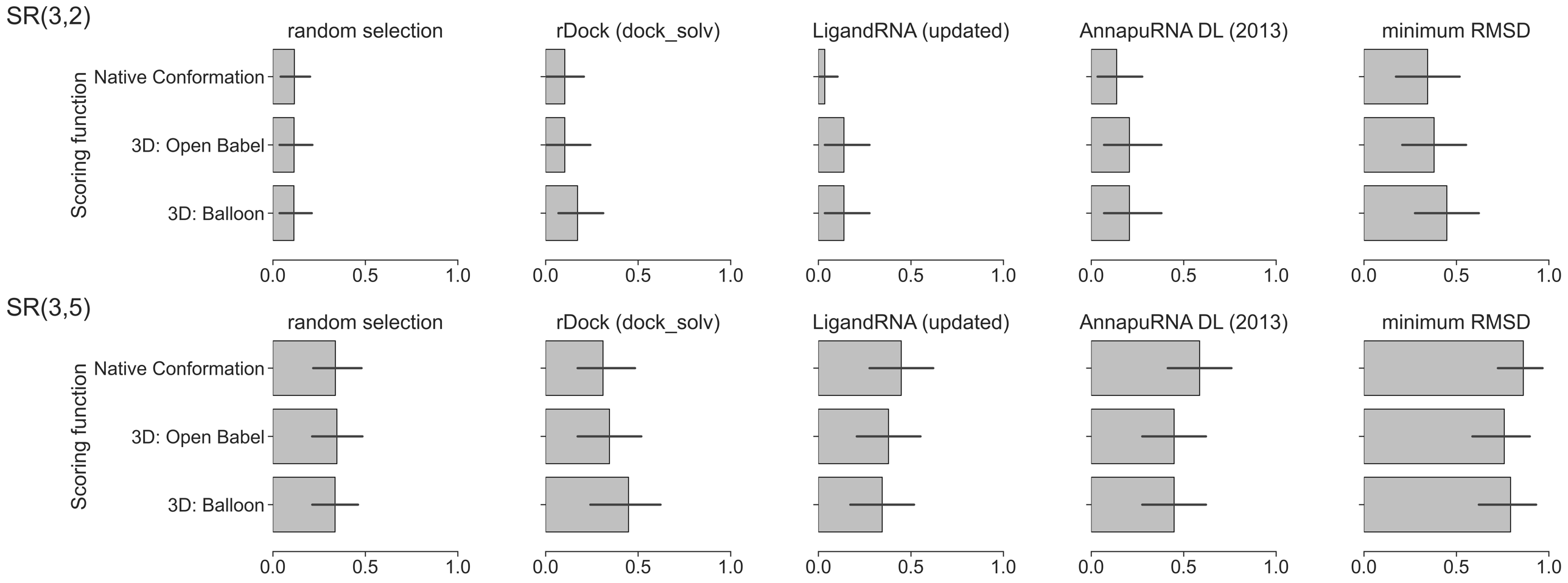

Supplement: S14 Fig — SR(X,C) indicates if a given docking was successful (i.e., in top X scoring poses there was at least one pose with RMSD ≤ C Å). The first column represents the performance obtained when the random poses are selected (the negative control). The last column represents the performance obtained when poses are ranked by the RMSD to the reference structure (positive control). Horizontal lines represent 95% bootstrapped confidence intervals. Docking was performed using rDock with the dock desolvation potential. (PNG) [file pcbi.1008309.s014.png]

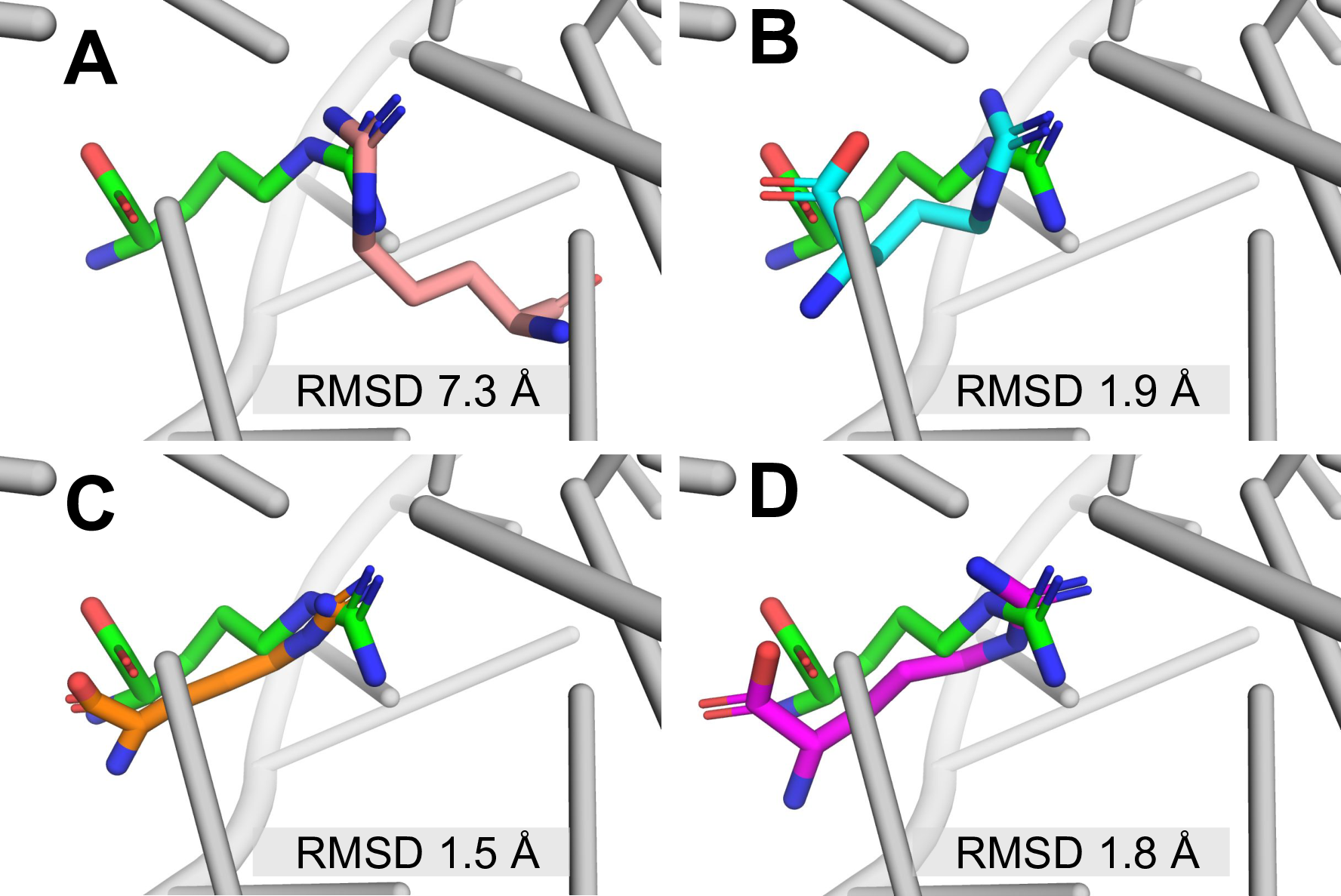

Supplement: S15 Fig — The best in the three top-scored solutions in redocking experiment (PDB ID: 1KOC) selected by the AnnapuRNA method without post processing (A), with clustering (AutoDock-like’ with 2 Å threshold, best in the three top-scored solutions, pane B), averaging the structure (C), and local optimization of the averaged ligand structure (D). RNA molecules are presented as a gray cartoon, ligands as sticks, heteroatoms are colored: O—red, N—blue, the reference ligand is green. Docking was performed using rDock with dock desolvation potential with the native conformation of a ligand as an input, and rescored using the AnnapuRNA DL 2016 method. (TIF) [file pcbi.1008309.s015.tif]

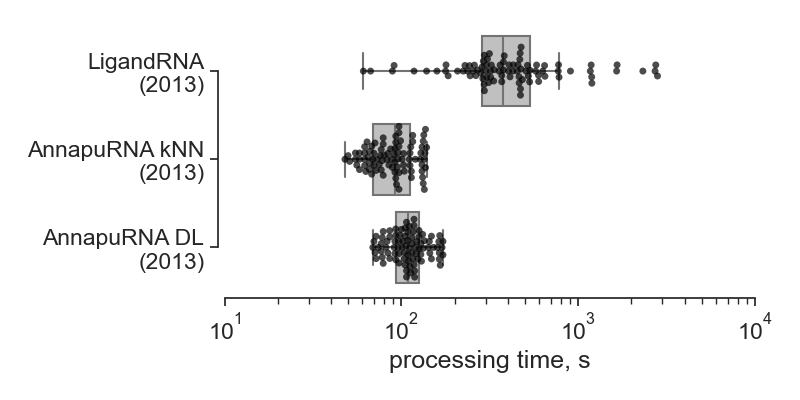

Supplement: S16 Fig — (PNG) [file pcbi.1008309.s016.png]

**A.**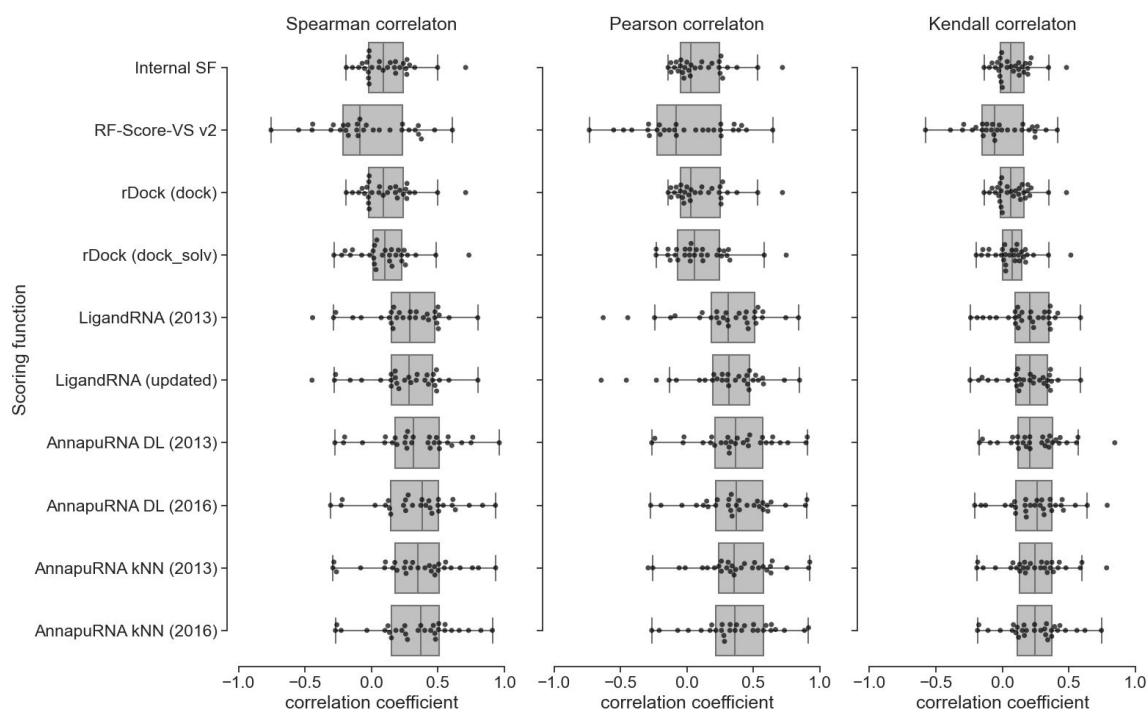**B.**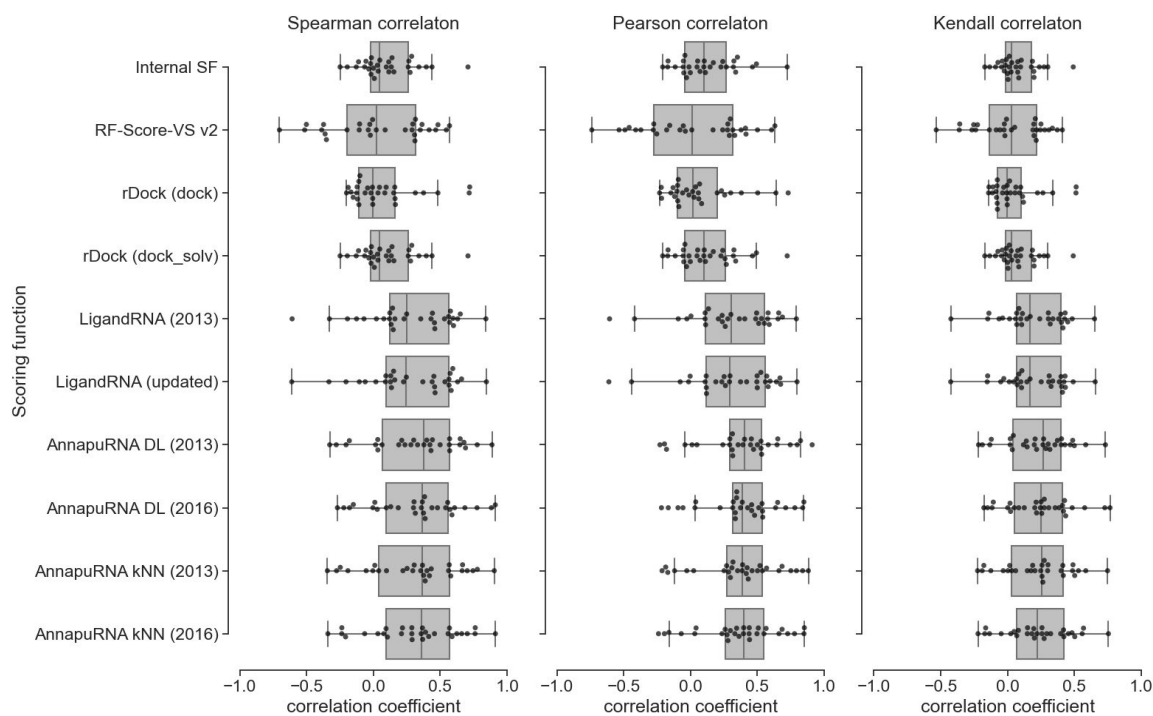

C.

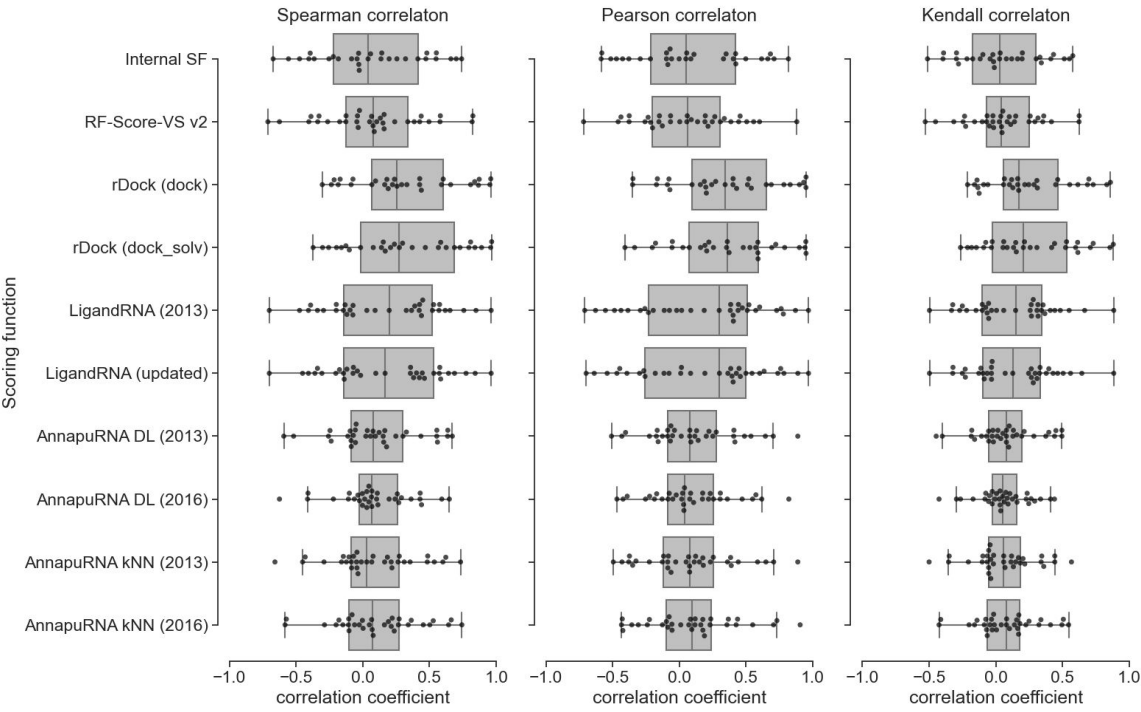

D.

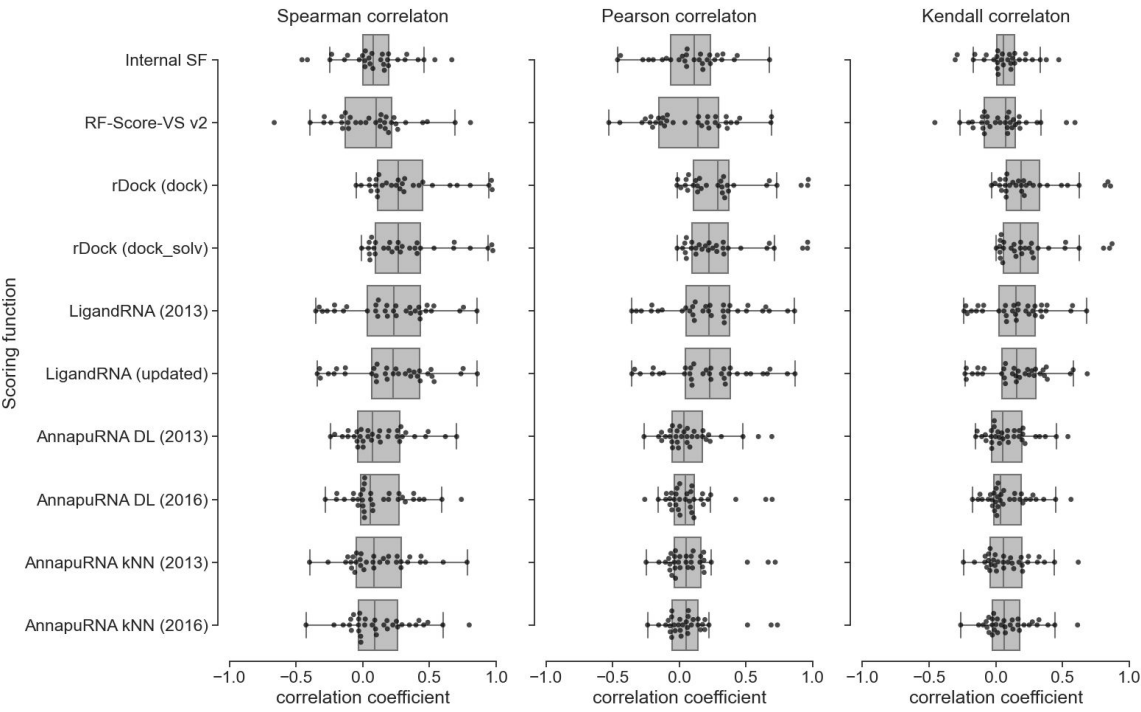

Supplement: S17 Fig — Additional rows represent the internal scoring function of the docking program (Internal SF). Each dot represents one complex from the testing set. Docking was performed using rDock with the dock and dock_solv desolvation potentials (A and B, respectively), iDock (B), and Autodock Vina (C). All docking was performed with native conformation of a ligand as an input. (PDF) [file pcbi.1008309.s017.pdf]
