# Supplementary material for: AnnapuRNA: A scoring function for predicting RNA-small molecule binding poses
Source: PLoS Comput Biol. 2021 Feb 1;17(2):e1008309. doi: 10.1371/journal.pcbi.1008309 (PMC7877745; doi:10.1371/journal.pcbi.1008309)
Supplement: S1 Table — For the cases, where PDB database mistakenly separated cocrystallized ligand into several chunks, the ligand was concatenated into a single molecule and ligand ID was indicated as “ligand”. E.g., for complex 1QD3, PDB database indicates four separated ligands (BDG, CYY, IDG, and RIB) instead of a single Neomycin B molecule. x for complexes from the testing set rDock docking program was not able to perform docking and generate poses. (PDF) [file pcbi.1008309.s018.pdf]

Structures in “2013” dataset				Additional structures in “2016” dataset		Testing set	
1AKX	ARG	3BNQ	PAR	1F27	BTN	1AJU	ARG
1ARJ	ARG	3C44	PAR	1ZZ5	CNY	1AM0	AMP
1FUF	SPM	3C5D	LIV	2KD4	PRL	1BYJ	ligand
1G4Q	MPD	3C7R	NMY	2KGP	MIX	1EHT	TEP
1J8G	SPM	3D0U	LYS	2KTZ	ISH	1EI2	NMY
1LC4	TOY	3D2G	TPP	2KU0	ISI	1FMN	FMN
1LVJ	PMZ	3D2V	PYI	2KX8	ARG	1FYP	PAR
1NJN	SPS	3DIG	SLZ	2L1V	PRF	1J7T	PAR
1NTA	SRY	3DIL	LYS	2L8H	L8H	1KOC	ARG
1NTB	SRY	3DIO	LYS	2L94	L94	1KOD	CIR
1O15	TEP	3DIQ	HRG	2LWK	0EC	1MWL	GET
1O9M	ligand	3DIR	IEL	2M4Q	AM2	1NBK	GND
1P9X	TEL	3DIY	LYS	2MIY	PRF	1NEM	ligand
1QD3	ligand	3DJ0	OLZ	2MXS	PAR	1PBR	ligand
1RAW	AMP	3DJ2	LYS	2NPZ	PDI	1Q8N	MGR
1YRJ	AM2	3DS7	GNG	2XNZ	3AW	1TOB	ligand
2A04	NMY	3DVV	RIO	3MIJ	R14	1UTS	P13
2AU4	GTP	3E5C	SAM	3Q3Z	C2E	1UUD	P14
2B57	6AP	3E5E	SAH	3Q50	PRF	1UUI	P12
2CKY	TPP	3F2Q	FMN	3RKF	DX4	1XPF	SPM
2EES	HPA	3FO6	6GO	3S4P	JS6	2BE0	JS5[x]
2EEU	HPA	3G4M	2BP	3WRU	SJP	2ET8	XXX
2ESI	KAN	3GAO	XAN	4ERL	GLY	2F4U	AB6
2ESJ	LIV	3GCA	PQ0	4F8U	SIS	2GDI	TPP
2ET3	LLL	3GLP	GOL	4F8V	SIS	2O3X	N30
2ET4	NMY	3GOT	A2F	4JF2	PRF	2OE5	AM2
2ET5	RIO	3GX3	SAH	4K32	GET	2PWT	LHA[x]
2F4S	XXX	3GX5	SAM	4LVW	7DG	2TOB	ligand
2F4T	AB9	3IQR	SAM	4LVX	H4B	3D2X	D2X
2FCX	XXX	3LA5	5AZ	4LVY	LYA	3GX2	SFG
2FCY	NMY	3NPQ	SAH	4LVZ	6AP	3SUX	THF
2G5K	AM2	3OWW	GLY	4LX5	29G	2BEE	JS4[x]
2G9C	3AY	3SD3	FOZ	4LX6	29H	2FCZ	RIO[x]
2GIS	SAM	3SKI	GNG	4NFO	SPM		
2GUN	SPM	3SKL	GNG	4P5J	SPM		
2JUK	G0B	3SKR	GNG	4P95	MES		
2KXM	RIO	3SKZ	GMP	4PDQ	NMZ		
2O3V	N33	3SUH	FFO	4QK8	2BA		
2O3W	PAR	3TD1	GET	4QK9	2BA		
2O3Y	SPM	3TZR	SS0	4YAZ	4BW		
2OE8	AM2	4FE5	HPA	4YB0	C2E		
2QWY	SAM	4KQY	SAM	4ZNP	AMZ		
2W89	GOL	4LVV	FFO	5BWS	V71		
		4P20	AKN	5BXK	S81		
