# Supplementary material for: AnnapuRNA: A scoring function for predicting RNA-small molecule binding poses
Source: PLoS Comput Biol. 2021 Feb 1;17(2):e1008309. doi: 10.1371/journal.pcbi.1008309 (PMC7877745; doi:10.1371/journal.pcbi.1008309)
Supplement: S2 Table — (PDF) [file pcbi.1008309.s019.pdf]

pdb1	pdb2	seq identity	RNA RMSD	dataset1	dataset2
1NTA	1NTB	100.0	0.7	2013,2016	2013,2016
2EES	4FE5	97.0	2.3	2013,2016	2013,2016
2EEU	4FE5	97.0	4.8	2013,2016	2013,2016
2GIS	3GX5	97.9	0.9	2013,2016	2013,2016
2GIS	3IQR	98.9	1.2	2013,2016	2013,2016
3C44	3C7R	100.0	1.7	2013,2016	2013,2016
3D0U	3DIL	91.3	3.6	2013,2016	2013,2016
3DIL	3DJ2	100.0	4.1	2013,2016	2013,2016
3TD1	4K32	100.0	0.7	2013,2016	2016
2KTZ	2KU0	100.0	2.0	2016	2016
4F8U	4F8V	100.0	2.2	2016	2016
5BWS	5BXK	100.0	1.6	2016	2016
1AKX	1AJU	100.0	3.0	2013,2016	testing
2ET4	1J7T	100.0	0.6	2013,2016	testing
2F4S	2ET8	100.0	1.6	2013,2016	testing
2OE8	2OE5	96.9	1.9	2013,2016	testing
1FYP	1PBR	96.3	4.8	testing	testing
