# Supplementary material for: AnnapuRNA: A scoring function for predicting RNA-small molecule binding poses
Source: PLoS Comput Biol. 2021 Feb 1;17(2):e1008309. doi: 10.1371/journal.pcbi.1008309 (PMC7877745; doi:10.1371/journal.pcbi.1008309)
Supplement: S3 Table — (PDF) [file pcbi.1008309.s020.pdf]

New structures in 2016	Sequence identity to RNAs in 2013			RMSD to RNAs in 2013			Ligand similarity to ligands in 2013		
	min	max	mean	min	max	mean	min	max	mean
1F27	0.00	100.00	38.88	5.63	31.93	16.60	3.52	43.41	30.43
1ZZ5	0.00	100.00	27.24	3.52	35.55	21.17	2.82	86.00	49.19
2KD4	0.00	100.00	30.56	3.11	32.30	13.98	0.77	24.84	17.25
2KGP	0.00	100.00	46.70	5.36	25.54	15.54	2.03	63.07	37.36
2KTZ	0.00	91.43	31.49	6.19	39.95	17.42	2.02	91.71	43.21
2KU0	0.00	91.43	31.49	5.72	39.20	17.67	2.02	91.71	43.21
2KX8	0.00	100.00	37.01	5.24	30.70	18.85	3.59	100.00	14.91
2L1V	6.25	100.00	41.76	5.37	35.16	17.18	1.48	84.53	32.88
2L8H	0.00	100.00	40.94	4.44	36.37	16.49	2.58	21.87	16.02
2L94	58.33	60.00	59.17	5.81	51.37	18.29	3.29	24.81	17.23
2LWK	61.29	76.92	66.50	2.66	32.64	16.88	2.44	56.44	35.00
2M4Q	0.00	100.00	67.51	4.91	29.33	14.99	3.05	100.00	48.53
2MIY	0.00	75.00	40.11	11.95	36.66	20.74	1.48	84.53	32.88
2MXS	62.96	100.00	75.31	5.87	33.89	16.01	3.30	100.00	49.22
2NPZ	65.00	65.00	65.00	7.98	30.28	19.29	3.19	20.34	6.45
2XNZ	0.00	100.00	43.71	0.48	34.20	18.60	2.03	29.61	9.61
3MIJ	0.00	66.67	34.77	5.34	36.24	11.96	1.97	64.60	38.20
3Q3Z	0.00	58.33	38.44	11.74	46.84	28.10	2.25	95.05	47.97
3Q50	14.29	100.00	47.79	1.12	31.66	17.00	1.48	84.53	32.88
3RKF	0.00	88.06	47.78	1.37	46.97	21.63	1.24	65.81	27.47
3S4P	0.00	100.00	51.89	1.32	31.04	15.71	3.12	89.24	48.59
3WRU	0.00	100.00	48.75	0.87	31.98	15.47	2.93	85.22	48.44
4ERL	0.00	100.00	44.25	3.55	47.02	21.25	0.74	100.00	6.85
4F8U	0.00	100.00	52.59	1.81	37.18	16.05	2.67	87.28	46.75
4F8V	0.00	100.00	51.89	1.34	30.95	15.64	2.67	87.28	46.75
4JF2	9.09	73.33	35.29	6.70	41.77	21.86	1.48	84.53	32.88
4K32	0.00	100.00	55.87	0.73	37.67	16.83	2.80	100.00	48.04
4LVW	60.67	100.00	80.06	0.68	35.75	22.09	2.20	48.30	30.72
4LVX	60.67	100.00	80.06	0.83	35.55	19.51	2.51	75.03	39.76
4LVY	60.67	100.00	80.06	0.66	35.73	21.62	2.38	75.92	36.01
4LVZ	60.67	100.00	80.06	0.86	35.79	19.54	1.03	100.00	27.48
4LX5	0.00	100.00	33.31	3.88	38.11	18.78	0.98	54.27	21.51
4LX6	0.00	100.00	33.68	1.71	38.30	20.08	1.55	52.01	23.44
4NFO	0.00	55.56	39.66	7.99	34.96	19.16	1.35	100.00	10.39
4P5J	7.69	58.82	33.02	7.15	37.08	20.00	1.35	100.00	10.39
4P95	21.05	60.87	46.66	5.06	46.98	25.23	4.76	16.75	12.90
4PDQ	0.00	100.00	50.34	2.93	34.35	17.06	2.76	84.07	49.00
4QK8	29.41	66.67	45.67	10.99	48.64	22.83	2.29	95.85	47.33
4QK9	0.00	66.67	48.66	11.28	38.48	24.18	2.29	95.85	47.33
4YAZ	44.44	60.00	53.11	9.53	48.39	26.18	2.17	92.86	48.58

**4YB0**	37.50	60.00	50.37	9.22	45.27	23.73	2.25	95.05	47.97
**4ZNP**	41.67	67.67	53.74	11.73	54.27	23.41	2.45	78.83	43.51
**5BWS**	0.00	91.11	48.53	2.12	35.66	18.77	3.64	80.99	44.49
**5BXK**	0.00	90.91	48.79	2.21	34.04	20.12	3.64	80.99	44.49

Number of structures in the 2016 dataset with RNA identity to any structure from 2013:

- < 90%: 16 structures
- < 75%: 13 structures
- < 50%: 0 structures

Number of structures in the 2016 dataset with RNA RMSD to any structure from 2013:

- > 2 Å: 31 structures
- > 5 Å: 21 structures
- > 7 Å: 10 structures
- > 10 Å: 5 structures

Number of structures in the 2016 dataset with ligand similarity to any structure from 2013:

- < 99%: 36 structures
- < 90%: 29 structures
- < 75%: 14 structures
- < 50%: 8 structures
- < 25%: 5 structures
