# Supplementary material for: AnnapuRNA: A scoring function for predicting RNA-small molecule binding poses
Source: PLoS Comput Biol. 2021 Feb 1;17(2):e1008309. doi: 10.1371/journal.pcbi.1008309 (PMC7877745; doi:10.1371/journal.pcbi.1008309)
Supplement: S4 Table — (PDF) [file pcbi.1008309.s021.pdf]

A - C2 - AROM	C - C2 - AROM	G - C2 - AROM	U - C2 - AROM
A - C2 - HACC	C - C2 - HACC	G - C2 - HACC	U - C2 - HACC
A - C2 - HDON	C - C2 - HDON	G - C2 - HDON	U - C2 - HDON
A - C2 - LIPO	C - C2 - LIPO	G - C2 - LIPO	U - C2 - LIPO
A - C2 - POSC	C - C2 - POSC	G - C2 - POSC	U - C2 - POSC
A - C4' - AROM	C - C4 - AROM	G - C4' - AROM	U - C4 - AROM
A - C4' - HACC	C - C4' - AROM	G - C4' - HACC	U - C4' - AROM
A - C4' - HDON	C - C4 - HACC	G - C4' - HDON	U - C4 - HACC
A - C4' - LIPO	C - C4' - HACC	G - C4' - LIPO	U - C4' - HACC
A - C4' - POSC	C - C4 - HDON	G - C4' - POSC	U - C4 - HDON
A - C6 - AROM	C - C4' - HDON	G - C6 - AROM	U - C4' - HDON
A - C6 - HACC	C - C4 - LIPO	G - C6 - HACC	U - C4 - LIPO
A - C6 - HDON	C - C4' - LIPO	G - C6 - HDON	U - C4' - LIPO
A - C6 - LIPO	C - C4 - POSC	G - C6 - LIPO	U - C4 - POSC
A - C6 - POSC	C - C4' - POSC	G - C6 - POSC	U - C4' - POSC
A - N9 - AROM	C - N1 - AROM	G - N9 - AROM	U - N1 - AROM
A - N9 - HACC	C - N1 - HACC	G - N9 - HACC	U - N1 - HACC
A - N9 - HDON	C - N1 - HDON	G - N9 - HDON	U - N1 - HDON
A - N9 - LIPO	C - N1 - LIPO	G - N9 - LIPO	U - N1 - LIPO
A - N9 - POSC	C - N1 - POSC	G - N9 - POSC	U - N1 - POSC
A - P - AROM	C - P - AROM	G - P - AROM	U - P - AROM
A - P - HACC	C - P - HACC	G - P - HACC	U - P - HACC
A - P - HDON	C - P - HDON	G - P - HDON	U - P - HDON
A - P - LIPO	C - P - LIPO	G - P - LIPO	U - P - LIPO
A - P - POSC	C - P - POSC	G - P - POSC	U - P - POSC
