# Supplementary material for: AnnapuRNA: A scoring function for predicting RNA-small molecule binding poses
Source: PLoS Comput Biol. 2021 Feb 1;17(2):e1008309. doi: 10.1371/journal.pcbi.1008309 (PMC7877745; doi:10.1371/journal.pcbi.1008309)
Supplement: S5 Table — (PDF) [file pcbi.1008309.s022.pdf]

**Classifier**	**Grid search parameter space and other parameters**
Deep Learning	`{'hidden': [[32, 32, 32, 32], [64, 64, 64], [200, 200], [128,128,128], [300, 300] ],`<br>`'activation': ["Rectifier","Tanh","Maxout","RectifierWithDropout","TanhWithDropout","MaxoutWithDropout"],`<br>`'input_dropout_ratio': [0,0.05],`<br>`‘'l1': [0,1e-4,1e-6], 'l2': [0,1e-4,1e-6] }`<br>`epochs=1000, balance_classes=False, stopping_metric='AUC', variable_importances=True, shuffle_training_data=True`
Gaussian Naïve Bayes	-
k Nearest Neighbors	`"n_neighbors": range(3, 11),`<br>`"leaf_size": [10,20,30,50],`<br>`"weights": ['uniform', 'distance'],`<br>`"algorithm":  ['ball_tree', 'kd_tree', 'brute']`
Random Forests	`"max_depth": [3, None],`<br>`"n_estimators": [5,10,15,20],`<br>`"min_samples_split": [1, 2, 3, 5, 10],`<br>`"min_samples_leaf": [1, 2, 3, 5, 10],`<br>`"bootstrap": [True, False],`<br>`"criterion": ["gini", "entropy"]`
Support Vector Machines	`"C":  sp_randint(1e-2, 1e6),`<br>`"gamma": sp_randint(1e-15, 1e2)`<br>`Kernel: RBF`
