# Supplementary material for: AnnapuRNA: A scoring function for predicting RNA-small molecule binding poses
Source: PLoS Comput Biol. 2021 Feb 1;17(2):e1008309. doi: 10.1371/journal.pcbi.1008309 (PMC7877745; doi:10.1371/journal.pcbi.1008309)
Supplement: S6 Table — S(3) is the averaged value for the cross-validation experiment. (PDF) [file pcbi.1008309.s023.pdf]

Distance cut off	*S*(3)					
	DL	GNB	kNN	RF	SVM	Average
6 Å	5.77	5.72	6.34	6.23	5.50	5.91
8 Å	4.95	5.19	4.77	5.57	5.15	5.12
10 Å	4.95	5.10	4.68	4.70	5.02	4.89
12 Å	5.55	5.35	5.32	5.01	5.07	5.26
Average	5.30	5.34	5.28	5.38	5.19	5.30
