# Supplementary material for: AnnapuRNA: A scoring function for predicting RNA-small molecule binding poses
Source: PLoS Comput Biol. 2021 Feb 1;17(2):e1008309. doi: 10.1371/journal.pcbi.1008309 (PMC7877745; doi:10.1371/journal.pcbi.1008309)
Supplement: S7 Table — S(3) is the averaged value for the cross-validation experiment. (PDF) [file pcbi.1008309.s024.pdf]

Distance binning step	Angle transformation and binning	*S*(3)	
		DL	kNN
-	-	4.95	4.68
0.01	binning 0.005	4.90	4.68
0.02	binning 0.010	4.74	4.71
0.02	cos + binning 0.010	4.87	4.80
0.02	cos + binning 0.020	4.98	4.84
0.04	binning 0.02	4.97	4.78
