# Supplementary material for: AnnapuRNA: A scoring function for predicting RNA-small molecule binding poses
Source: PLoS Comput Biol. 2021 Feb 1;17(2):e1008309. doi: 10.1371/journal.pcbi.1008309 (PMC7877745; doi:10.1371/journal.pcbi.1008309)
Supplement: S8 Table — S(3) is the averaged value for cross-validation experiment. (PDF) [file pcbi.1008309.s025.pdf]

transformation	$S(3)$		
	DL	kNN	average
no transformation	4.95	4.68	4.82
1/x	5.27	4.89	5.08
L-J	6.77	5.59	6.18
linear	5.47	4.77	5.12
