# Supplementary material for: AnnapuRNA: A scoring function for predicting RNA-small molecule binding poses
Source: PLoS Comput Biol. 2021 Feb 1;17(2):e1008309. doi: 10.1371/journal.pcbi.1008309 (PMC7877745; doi:10.1371/journal.pcbi.1008309)
Supplement: S9 Table — (PDF) [file pcbi.1008309.s026.pdf]

noise removal method	*S*(3)		
	DL	kNN	average
none	4.95	4.77	4.86
ENN	4.63	3.87	4.25
TL	4.32	4.54	4.43
