# Supplementary material for: AnnapuRNA: A scoring function for predicting RNA-small molecule binding poses
Source: PLoS Comput Biol. 2021 Feb 1;17(2):e1008309. doi: 10.1371/journal.pcbi.1008309 (PMC7877745; doi:10.1371/journal.pcbi.1008309)
Supplement: S10 Table — (PDF) [file pcbi.1008309.s027.pdf]

Scoring function	*w*	*S*(1)	*S*(3)	Pareto rank
DL	0.00	5.60	4.63	1
DL	0.01	5.29	4.86	1
DL	0.02	5.34	4.86	1
DL	0.05	5.34	4.86	1
DL	0.10	5.27	4.86	1
DL	0.50	5.22	4.74	1
DL	1.00	5.42	4.65	1
kNN	0.00	5.65	3.87	3
kNN	0.01	6.04	3.92	1
kNN	0.02	6.33	3.83	1
kNN	0.05	6.33	3.99	2
kNN	0.10	6.27	3.93	1
kNN	0.50	6.23	3.71	3
kNN	1.00	6.00	3.86	3
