# Supplementary material for: AnnapuRNA: A scoring function for predicting RNA-small molecule binding poses
Source: PLoS Comput Biol. 2021 Feb 1;17(2):e1008309. doi: 10.1371/journal.pcbi.1008309 (PMC7877745; doi:10.1371/journal.pcbi.1008309)
Supplement: S11 Table — Values represent averages for the testing set. Docking was performed using rDock with the dock desolvation potential, with the native conformation of a ligand as an input. (PDF) [file pcbi.1008309.s028.pdf]

| scoring function | *S*(1) | | | *S*(3) | | | *S*(5) | | |
|---|---|---|---|---|---|---|---|---|---|
| RF-Score-VS v2 | 8.60 | ± | 3.27 | 6.94 | ± | 3.18 | 6.27 | ± | 3.29 |
| rDock (dock) | 6.81 | ± | 3.41 | 5.60 | ± | 3.01 | 5.23 | ± | 2.82 |
| rDock (dock_solv) | 6.92 | ± | 3.33 | 5.80 | ± | 2.94 | 5.12 | ± | 2.69 |
| LigandRNA (2013) | 6.67 | ± | 2.82 | 5.51 | ± | 2.31 | 4.92 | ± | 2.38 |
| LigandRNA (updated) | 6.37 | ± | 2.74 | 5.49 | ± | 2.23 | 5.00 | ± | 2.36 |
| AnnapuRNA DL (2013) | 5.39 | ± | 2.85 | 4.58 | ± | 2.47 | 4.37 | ± | 2.21 |
| AnnapuRNA DL (2016) | 6.68 | ± | 3.04 | 4.82 | ± | 2.66 | 4.15 | ± | 2.24 |
| AnnapuRNA kNN (2013) | 6.06 | ± | 2.92 | 4.86 | ± | 2.56 | 4.14 | ± | 2.18 |
| AnnapuRNA kNN (2016) | 5.76 | ± | 2.87 | 5.00 | ± | 2.64 | 4.35 | ± | 2.32 |
| minimum RMSD | 2.90 | ± | 1.76 | 2.90 | ± | 1.76 | 2.90 | ± | 1.76 |
| median RMSD | 7.61 | ± | 2.70 | 7.61 | ± | 2.70 | 7.61 | ± | 2.70 |
