# Supplementary material for: AnnapuRNA: A scoring function for predicting RNA-small molecule binding poses
Source: PLoS Comput Biol. 2021 Feb 1;17(2):e1008309. doi: 10.1371/journal.pcbi.1008309 (PMC7877745; doi:10.1371/journal.pcbi.1008309)
Supplement: S12 Table — Values are highlighted for cases, where the performance is worse for the non-redundant testing set than for the full testing set. (PDF) [file pcbi.1008309.s029.pdf]

scoring function	Full test set							Non-redundant-test set						
	**S1**	**S3**	**S5**	**SR(3,2)**	**SR(3,5)**	**SR(5,2)**	**SR(5,5)**	**S1**	**S3**	**S5**	**SR(3,2)**	**SR(3,5)**	**SR(5,2)**	**SR(5,5)**
**AnnapuRNA DL (2013)**	5.39	4.58	4.37	0.14	0.59	0.14	0.59	5.09	4.26	4.20	0.16	0.64	0.16	0.64
**AnnapuRNA DL (2016)**	6.68	4.82	4.15	0.14	0.48	0.17	0.62	6.59	4.53	3.94	0.16	0.52	0.20	0.68
**AnnapuRNA kNN (2013)**	6.06	4.86	4.14	0.14	0.52	0.17	0.66	5.87	4.57	3.93	0.16	0.56	0.20	0.72
**AnnapuRNA kNN (2016)**	5.76	5.00	4.35	0.14	0.55	0.17	0.62	5.52	4.74	4.09	0.16	0.60	0.16	0.68
**LigandRNA (2013)**	6.67	5.51	4.92	0.03	0.48	0.10	0.55	6.25	5.46	4.78	0.04	0.52	0.12	0.60
**LigandRNA (updated)**	6.37	5.49	5.00	0.03	0.45	0.10	0.52	6.16	5.43	4.88	0.04	0.48	0.12	0.56
**RF-Score-VS v2**	8.60	6.94	6.27	0.00	0.28	0.03	0.34	7.97	6.28	5.69	0.00	0.32	0.04	0.40
**rDock (dock)**	6.81	5.60	5.23	0.14	0.34	0.21	0.38	6.38	5.11	4.87	0.16	0.40	0.24	0.40
**rDock (dock_solv)**	6.92	5.80	5.12	0.10	0.31	0.14	0.38	6.46	5.31	4.74	0.12	0.36	0.16	0.44
