# Supplementary material for: AnnapuRNA: A scoring function for predicting RNA-small molecule binding poses
Source: PLoS Comput Biol. 2021 Feb 1;17(2):e1008309. doi: 10.1371/journal.pcbi.1008309 (PMC7877745; doi:10.1371/journal.pcbi.1008309)
Supplement: S13 Table — (PDF) [file pcbi.1008309.s030.pdf]

conformation	docking program	scoring function	SR(3,2)	SR(5,2)	SR(3,5)	SR(5,5)
**3D: Balloon**	**Autodock Vina**	AnnapuRNA DL (2013)	0.00	0.00	0.18	0.30
		AnnapuRNA DL (2016)	0.00	0.00	0.27	0.30
		AnnapuRNA kNN (2013)	0.00	0.00	0.27	0.27
		AnnapuRNA kNN (2016)	0.00	0.00	0.27	0.30
		LigandRNA (2013)	0.00	0.00	0.24	0.24
		LigandRNA (updated)	0.00	0.00	0.24	0.24
		minimum RMSD	0.00	0.00	0.39	0.39
		random selection	0.00	0.00	0.23	0.29
		rDock (dock_solv)	0.00	0.00	0.21	0.27
		rDock (dock)	0.00	0.00	0.21	0.27
		RF-Score-VS v2	0.00	0.00	0.21	0.21
	**iDock**	AnnapuRNA DL (2013)	0.00	0.00	0.27	0.30
		AnnapuRNA DL (2016)	0.00	0.00	0.24	0.24
		AnnapuRNA kNN (2013)	0.00	0.00	0.27	0.27
		AnnapuRNA kNN (2016)	0.00	0.00	0.27	0.27
		LigandRNA (2013)	0.00	0.00	0.21	0.24
		LigandRNA (updated)	0.00	0.00	0.24	0.24
		minimum RMSD	0.00	0.00	0.48	0.48
		random selection	0.00	0.00	0.19	0.24
		rDock (dock_solv)	0.00	0.00	0.21	0.30
		rDock (dock)	0.00	0.00	0.27	0.30
		RF-Score-VS v2	0.00	0.00	0.18	0.21
	**rDock (dock_solv)**	AnnapuRNA DL (2013)	0.21	0.21	0.52	0.69
		AnnapuRNA DL (2016)	0.24	0.24	0.48	0.55
		AnnapuRNA kNN (2013)	0.24	0.28	0.59	0.69
		AnnapuRNA kNN (2016)	0.21	0.24	0.55	0.66
		LigandRNA (2013)	0.24	0.24	0.45	0.55
		LigandRNA (updated)	0.21	0.24	0.48	0.55
		minimum RMSD	0.48	0.48	0.86	0.86
		random selection	0.12	0.16	0.35	0.43
		rDock (dock_solv)	0.21	0.24	0.38	0.45
		rDock (dock)	0.24	0.28	0.34	0.45
		RF-Score-VS v2	0.07	0.14	0.28	0.38
	**rDock (dock)**	AnnapuRNA DL (2013)	0.21	0.21	0.45	0.45
		AnnapuRNA DL (2016)	0.21	0.24	0.45	0.48
		AnnapuRNA kNN (2013)	0.21	0.24	0.45	0.45
		AnnapuRNA kNN (2016)	0.21	0.24	0.41	0.48
		LigandRNA (2013)	0.14	0.14	0.34	0.48
		LigandRNA (updated)	0.14	0.14	0.34	0.41
		minimum RMSD	0.45	0.45	0.79	0.79
		random selection	0.11	0.15	0.34	0.41

		rDock (dock_solv)	0.17	0.21	0.45	0.52
		rDock (dock)	0.17	0.21	0.38	0.45
		RF-Score-VS v2	0.07	0.07	0.21	0.28
**3D: Open Babel**	**Autodock Vina**	AnnapuRNA DL (2013)	0.00	0.00	0.27	0.27
		AnnapuRNA DL (2016)	0.00	0.00	0.24	0.24
		AnnapuRNA kNN (2013)	0.00	0.00	0.27	0.27
		AnnapuRNA kNN (2016)	0.00	0.00	0.27	0.27
		LigandRNA (2013)	0.00	0.00	0.18	0.21
		LigandRNA (updated)	0.00	0.00	0.21	0.21
		minimum RMSD	0.00	0.00	0.33	0.33
		random selection	0.00	0.00	0.18	0.22
		rDock (dock_solv)	0.00	0.00	0.21	0.27
		rDock (dock)	0.00	0.00	0.18	0.24
		RF-Score-VS v2	0.00	0.00	0.15	0.18
	**iDock**	AnnapuRNA DL (2013)	0.00	0.00	0.18	0.27
		AnnapuRNA DL (2016)	0.00	0.00	0.21	0.24
		AnnapuRNA kNN (2013)	0.00	0.00	0.21	0.27
		AnnapuRNA kNN (2016)	0.00	0.00	0.18	0.27
		LigandRNA (2013)	0.00	0.00	0.21	0.24
		LigandRNA (updated)	0.00	0.00	0.21	0.24
		minimum RMSD	0.00	0.00	0.48	0.48
		random selection	0.00	0.00	0.18	0.22
		rDock (dock_solv)	0.00	0.00	0.21	0.24
		rDock (dock)	0.00	0.00	0.21	0.24
		RF-Score-VS v2	0.00	0.00	0.12	0.12
	**rDock (dock_solv)**	AnnapuRNA DL (2013)	0.21	0.24	0.45	0.52
		AnnapuRNA DL (2016)	0.24	0.31	0.48	0.59
		AnnapuRNA kNN (2013)	0.28	0.28	0.52	0.59
		AnnapuRNA kNN (2016)	0.24	0.24	0.45	0.59
		LigandRNA (2013)	0.24	0.24	0.34	0.41
		LigandRNA (updated)	0.24	0.24	0.34	0.41
		minimum RMSD	0.41	0.41	0.86	0.86
		random selection	0.12	0.16	0.38	0.46
		rDock (dock_solv)	0.14	0.17	0.48	0.52
		rDock (dock)	0.21	0.24	0.41	0.48
		RF-Score-VS v2	0.03	0.10	0.21	0.41
	**rDock (dock)**	AnnapuRNA DL (2013)	0.21	0.21	0.45	0.48
		AnnapuRNA DL (2016)	0.24	0.28	0.45	0.55
		AnnapuRNA kNN (2013)	0.21	0.24	0.41	0.52
		AnnapuRNA kNN (2016)	0.21	0.21	0.45	0.52
		LigandRNA (2013)	0.14	0.17	0.38	0.45
		LigandRNA (updated)	0.14	0.14	0.38	0.45
		minimum RMSD	0.38	0.38	0.76	0.76

		random selection	0.11	0.15	0.35	0.42
		rDock (dock_solv)	0.10	0.17	0.34	0.48
		rDock (dock)	0.14	0.17	0.34	0.45
		RF-Score-VS v2	0.00	0.03	0.21	0.34
**Native Conformation**	**Autodock Vina**	AnnapuRNA DL (2013)	0.00	0.00	0.27	0.27
		AnnapuRNA DL (2016)	0.00	0.00	0.27	0.27
		AnnapuRNA kNN (2013)	0.00	0.00	0.27	0.30
		AnnapuRNA kNN (2016)	0.00	0.00	0.24	0.30
		LigandRNA (2013)	0.00	0.00	0.24	0.27
		LigandRNA (updated)	0.00	0.00	0.24	0.27
		minimum RMSD	0.00	0.00	0.36	0.36
		random selection	0.00	0.00	0.21	0.26
		rDock (dock_solv)	0.00	0.00	0.27	0.30
		rDock (dock)	0.00	0.00	0.30	0.30
		RF-Score-VS v2	0.00	0.00	0.30	0.30
	**iDock**	AnnapuRNA DL (2013)	0.06	0.06	0.27	0.30
		AnnapuRNA DL (2016)	0.06	0.06	0.24	0.30
		AnnapuRNA kNN (2013)	0.06	0.06	0.21	0.24
		AnnapuRNA kNN (2016)	0.06	0.06	0.24	0.33
		LigandRNA (2013)	0.06	0.06	0.24	0.27
		LigandRNA (updated)	0.06	0.06	0.24	0.27
		minimum RMSD	0.06	0.06	0.45	0.45
		random selection	0.00	0.00	0.20	0.25
		rDock (dock_solv)	0.06	0.06	0.27	0.30
		rDock (dock)	0.06	0.06	0.33	0.33
		RF-Score-VS v2	0.03	0.03	0.21	0.30
	**rDock (dock_solv)**	AnnapuRNA DL (2013)	0.24	0.24	0.59	0.69
		AnnapuRNA DL (2016)	0.24	0.28	0.55	0.72
		AnnapuRNA kNN (2013)	0.28	0.28	0.62	0.66
		AnnapuRNA kNN (2016)	0.21	0.28	0.59	0.66
		LigandRNA (2013)	0.10	0.10	0.59	0.59
		LigandRNA (updated)	0.10	0.10	0.55	0.59
		minimum RMSD	0.45	0.45	0.90	0.90
		random selection	0.10	0.14	0.35	0.44
		rDock (dock_solv)	0.17	0.17	0.41	0.45
		rDock (dock)	0.17	0.21	0.34	0.34
		RF-Score-VS v2	0.07	0.10	0.28	0.34
	**rDock (dock)**	AnnapuRNA DL (2013)	**0.14**	**0.14**	**0.59**	**0.59**
		AnnapuRNA DL (2016)	**0.14**	**0.17**	**0.48**	**0.62**
		AnnapuRNA kNN (2013)	**0.14**	**0.17**	**0.52**	**0.66**
		AnnapuRNA kNN (2016)	**0.14**	**0.17**	**0.55**	**0.62**
		LigandRNA (2013)	**0.03**	**0.10**	**0.48**	**0.55**
		LigandRNA (updated)	**0.03**	**0.10**	**0.45**	**0.52**

		minimum RMSD	**0.34**	**0.34**	**0.86**	**0.86**
		random selection	**0.12**	**0.15**	**0.34**	**0.41**
		rDock (dock_solv)	**0.10**	**0.14**	**0.31**	**0.38**
		rDock (dock)	**0.14**	**0.21**	**0.34**	**0.38**
		RF-Score-VS v2	**0.00**	**0.03**	**0.28**	**0.34**
