# Supplementary material for: AnnapuRNA: A scoring function for predicting RNA-small molecule binding poses
Source: PLoS Comput Biol. 2021 Feb 1;17(2):e1008309. doi: 10.1371/journal.pcbi.1008309 (PMC7877745; doi:10.1371/journal.pcbi.1008309)
Supplement: S14 Table — Docking was performed using a native conformation of a ligand as an input. If for a given structure a program was not able to complete the docking successfully, the row was grayed out. If for a given structure a docking program was not able to find a solution with RMSD ≤ 10 Å, the row was marked red. The bottom rows summarizes the performance for all data presented (A), only for a set of structures, for which all programs completed docking (B), and only for a set of structures, for which RMSD values of poses found by all programs were below or equal to 10 Å (C). (PDF) [file pcbi.1008309.s031.pdf]

PDB	AutodockVina	iDock	rdock-dock	rdock-dock_solv
**1AJU**	3.2	2.6	1.2	1.2
**1AM0**	3.8	3.5	1.2	1.2
**1BYJ**	4.6	4.2	1.2	0.9
**1EHT**	3.5	1.9	0.5	0.5
**1EI2**	5.3	5.4	4.5	4.6
**1FMN**	2.4	2.2	1.5	1.3
**1FYP**	5.0	5.3	1.1	1.2
**1J7T**	13.3	10.8	5.1	3.8
**1KOC**	3.1	3.0	1.2	1.4
**1KOD**	2.3	2.1	1.5	1.9
**1MWL**	11.3	11.5	4.8	5.0
**1NBK**	4.6	4.0	1.8	1.4
**1NEM**	5.6	5.6	2.3	1.9
**1PBR**	5.1	4.8	3.1	1.4
**1Q8N**	6.1	5.0	0.4	0.4
**1TOB**	5.3	5.3	2.6	2.6
**1UTS**	7.2	4.4	2.1	2.3
**1UUD**	3.4	4.0	3.0	2.6
**1UUI**	5.6	4.4	3.7	4.8
**1XPF**	3.7	3.2	3.8	4.0
**2BE0**	14.2	12.7	no solutions found	no solutions found
**2BEE**	10.8	10.9	no solutions found	no solutions found
**2ET8**	4.8	4.2	4.4	2.7
**2F4U**	6.2	6.0	5.0	5.6
**2FCZ**	8.9	7.3	no solutions found	no solutions found
**2GDI**	32.3	31.1	1.6	2.3
**2O3X**	4.3	4.1	4.9	4.3
**2OE5**	4.1	4.2	5.4	2.6
**2PWT**	19.1	19.6	no solutions found	no solutions found
**2TOB**	5.6	5.6	1.8	1.4
**3D2X**	12.1	10.8	2.5	1.8
**3GX2**	3.8	3.7	1.9	0.8
**3SUX**	2.6	0.9	0.7	0.8
**A.** All data (33 structures):				
**Average**	**7.1**	**6.5**	**2.6**	**2.3**
**Median**	**5.1**	**4.4**	**2.1**	**1.9**
**B.** Docking completed for all four docking programs (29 structures):				
**Average**	**6.2**	**5.6**	**2.6**	**2.3**
**Median**	**4.8**	**4.2**	**2.1**	**1.9**
**C.** Docking completed for all four docking programs and all RMSD values < 10 Å (25 structures):				
**Average**	**4.4**	**4.0**	**2.4**	**2.2**
**Median**	**4.6**	**4.2**	**1.9**	**1.4**
