# Supplementary material for: AnnapuRNA: A scoring function for predicting RNA-small molecule binding poses
Source: PLoS Comput Biol. 2021 Feb 1;17(2):e1008309. doi: 10.1371/journal.pcbi.1008309 (PMC7877745; doi:10.1371/journal.pcbi.1008309)
Supplement: S15 Table — Additional column ([RMSD]) shows the lowest RMSD poses obtained during docking. Docking was performed with the native conformation of a ligand as an input. (PDF) [file pcbi.1008309.s032.pdf]

**RMSD of the best poses**	Scoring function, S(3)											
Docking program	AnnapuRNA:DL (2013)	AnnapuRNA:DL (2016)	AnnapuRNA:kNN (2013)	AnnapuRNA:kNN (2016)	internal SF	Ligand RNA (basic)	Ligand RNA (updated)	rDock (dock)	rDock (dock_solv)	RF-Score-VS v2	[RMSD]	**Average**
AutodockVina	8.58	8.53	8.58	8.59	8.84	8.75	8.74	7.99	8.03	9.06	7.58	**8.48**
iDock	8.44	8.39	8.67	8.54	8.88	8.55	8.55	7.75	8.08	9.01	6.75	**8.33**
rdock-dock	4.58	4.82	4.86	5.00	5.60	5.51	5.49	5.60	5.80	6.94	2.90	**5.19**
rdock-dock_solv	4.37	4.75	4.36	4.51	5.08	4.71	4.86	5.76	5.08	6.88	2.58	**4.81**
