# Supplementary material for: AnnapuRNA: A scoring function for predicting RNA-small molecule binding poses
Source: PLoS Comput Biol. 2021 Feb 1;17(2):e1008309. doi: 10.1371/journal.pcbi.1008309 (PMC7877745; doi:10.1371/journal.pcbi.1008309)
Supplement: S16 Table — SR(X,C) indicates if a given docking was successful (i.e., in top X scoring poses there was at least one pose with RMSD ≤ C Å). The first column represents the performance obtained when the random poses are selected (the negative control). The last column represents the performance obtained when poses are ranked by the RMSD to the reference structure (positive control). Docking was performed with the native conformation of a ligand as an input. (PDF) [file pcbi.1008309.s033.pdf]

		Scoring function				
	Docking program	**random selection**	**rDock (dock_solv)**	**LigandRNA (updated)**	**AnnapuRNA DL (2013)**	**minimum RMSD**
**SR(3,2)**	**Autodock Vina**	0.00	0.00	0.00	0.00	0.00
	**iDock**	0.00	0.07	0.07	0.07	0.07
	**rDock (dock)**	0.12	0.10	0.03	0.14	0.34
	rDock (dock_solv)	0.10	0.17	0.10	0.24	0.45
**SR(3,5)**	**Autodock Vina**	0.24	0.31	0.28	0.31	0.41
	**iDock**	0.23	0.31	0.28	0.31	0.52
	**rDock (dock)**	0.34	0.31	0.45	0.59	0.86
	rDock (dock_solv)	0.35	0.41	0.55	0.59	0.90
**SR(5,2)**	**Autodock Vina**	0.00	0.00	0.00	0.00	0.00
	**iDock**	0.00	0.07	0.07	0.07	0.07
	**rDock (dock)**	0.15	0.14	0.10	0.14	0.34
	rDock (dock_solv)	0.14	0.17	0.10	0.24	0.45
**SR(5,5)**	**Autodock Vina**	0.29	0.34	0.31	0.31	0.41
	**iDock**	0.29	0.34	0.31	0.34	0.52
	**rDock (dock)**	0.41	0.38	0.52	0.59	0.86
	rDock (dock_solv)	0.44	0.45	0.59	0.69	0.90
