# Supplementary material for: AnnapuRNA: A scoring function for predicting RNA-small molecule binding poses
Source: PLoS Comput Biol. 2021 Feb 1;17(2):e1008309. doi: 10.1371/journal.pcbi.1008309 (PMC7877745; doi:10.1371/journal.pcbi.1008309)
Supplement: S17 Table — SR(X,C) indicates if a given docking was successful (i.e., in top X scoring poses there was at least one pose with RMSD ≤ C Å). The first column represents the performance obtained when the random poses are selected (the negative control). The last column represents the performance obtained when poses are ranked by the RMSD to the reference structure (positive control). Docking was performed using rDock with the dock desolvation potential. (PDF) [file pcbi.1008309.s034.pdf]

		Scoring function				
	**Conformer generation method**	**random selection**	**rDock (dock_solv)**	**LigandRNA (updated)**	**AnnapuRNA DL (2013)**	**minimum RMSD**
**SR(3,2)**	**3D: Balloon**	0.11	0.17	0.14	0.21	0.45
	**3D: Open Babel**	0.11	0.10	0.14	0.21	0.38
	**Native Conformation**	0.12	0.10	0.03	0.14	0.34
**SR(3,5)**	**3D: Balloon**	0.34	0.45	0.34	0.45	0.79
	**3D: Open Babel**	0.35	0.34	0.38	0.45	0.76
	**Native Conformation**	0.34	0.31	0.45	0.59	0.86
**SR(5,2)**	**3D: Balloon**	0.15	0.21	0.14	0.21	0.45
	**3D: Open Babel**	0.15	0.17	0.14	0.21	0.38
	**Native Conformation**	0.15	0.14	0.10	0.14	0.34
**SR(5,5)**	**3D: Balloon**	0.41	0.52	0.41	0.45	0.79
	**3D: Open Babel**	0.42	0.48	0.45	0.48	0.76
	Native Conformation	0.41	0.38	0.52	0.59	0.86
