# Supplementary material for: AnnapuRNA: A scoring function for predicting RNA-small molecule binding poses
Source: PLoS Comput Biol. 2021 Feb 1;17(2):e1008309. doi: 10.1371/journal.pcbi.1008309 (PMC7877745; doi:10.1371/journal.pcbi.1008309)
Supplement: S18 Table — Additional column ([RMSD]) shows the lowest RMSD poses obtained during the docking. (PDF) [file pcbi.1008309.s035.pdf]

**RMSD of the best poses**		Scoring function											
conformation	Docking program	AnnapuRNA:DL (2013)	AnnapuRNA:DL (2016)	AnnapuRNA:kNN (2013)	AnnapuRNA:kNN (2016)	internal SF	LigandRNA (basic)	LigandRNA (updated)	rDock (dock)	rDock (dock_solv)	RF-Score-VS v2	[RMSD]	**Average**
3D: Open Babel	AutodockVina	9.45	9.50	9.63	9.51	9.81	9.75	9.55	9.46	9.45	10.14	7.55	**9.43**
	iDock	9.42	9.45	9.37	9.25	9.63	9.85	9.85	9.20	9.18	10.32	6.98	**9.32**
	rdock-dock	5.66	6.29	6.36	6.49	7.10	7.10	7.05	7.10	7.01	8.57	3.12	**6.53**
	rdock-dock_solv	6.12	5.60	6.18	6.23	6.20	6.33	6.33	7.14	6.20	8.30	2.74	**6.13**
3D: Balloon	AutodockVina	9.52	9.56	9.45	9.55	10.25	10.15	10.22	9.32	9.87	10.20	7.64	**9.61**
	iDock	10.00	9.88	9.65	9.68	10.14	10.16	10.32	9.64	9.50	9.91	6.97	**9.62**
	rdock-dock	5.78	6.13	6.29	6.46	6.18	7.33	7.24	6.18	6.22	8.31	2.86	**6.27**
	rdock-dock_solv	5.44	6.23	5.31	5.58	7.33	6.51	6.51	7.34	7.33	7.92	2.72	**6.20**
Native Conformation	AutodockVina	9.32	9.10	9.54	9.27	9.57	9.37	9.33	8.97	8.85	10.04	7.58	**9.18**
	iDock	9.92	9.50	9.63	9.41	9.38	9.95	10.03	8.70	9.01	10.19	6.75	**9.32**
	rdock-dock	5.39	6.68	6.06	5.76	6.81	6.67	6.37	6.81	6.92	8.60	2.90	**6.27**
	rdock-dock_solv	5.36	5.61	5.08	5.33	7.36	6.03	6.09	7.63	7.36	8.38	2.58	**6.07**

**S(3)**		Scoring function											
conformation	Docking program	AnnapuRNA:DL (2013)	AnnapuRNA:DL (2016)	AnnapuRNA:kNN (2013)	AnnapuRNA:kNN (2016)	internal SF	LigandRNA (basic)	LigandRNA (updated)	rDock (dock)	rDock (dock_solv)	RF-Score-VS v2	[RMSD]	**Average**
3D: Open Babel	AutodockVina	8.53	8.82	8.72	8.63	9.16	8.86	8.82	8.71	8.69	9.42	7.55	**8.72**
	iDock	8.68	8.78	8.86	8.73	8.84	9.14	9.06	8.50	8.50	8.96	6.98	**8.64**
	rdock-dock	4.78	4.91	4.98	4.91	5.54	5.64	5.68	5.54	5.65	7.27	3.12	**5.27**
	rdock-dock_solv	4.92	4.77	4.68	4.89	5.18	5.24	5.26	5.24	5.18	7.13	2.74	**5.02**
3D: Balloon	AutodockVina	9.08	8.89	8.91	8.86	9.17	9.10	9.09	8.48	8.53	9.00	7.64	**8.80**
	iDock	8.45	8.94	8.44	8.76	9.35	9.30	9.28	8.57	8.55	9.03	6.97	**8.69**
	rdock-dock	4.62	4.83	4.64	4.69	5.24	5.60	5.60	5.24	4.84	7.23	2.86	**5.04**
	rdock-dock_solv	4.97	4.87	4.37	5.00	5.10	5.26	5.27	5.62	5.10	6.60	2.72	**4.99**
Native Conformation	AutodockVina	8.58	8.53	8.58	8.59	8.84	8.75	8.74	7.99	8.03	9.06	7.58	**8.48**
	iDock	8.44	8.39	8.67	8.54	8.88	8.55	8.55	7.75	8.08	9.01	6.75	**8.33**
	rdock-dock	4.58	4.82	4.86	5.00	5.60	5.51	5.49	5.60	5.80	6.94	2.90	**5.19**
	rdock-dock_solv	4.37	4.75	4.36	4.51	5.08	4.71	4.86	5.76	5.08	6.88	2.58	**4.81**

**S(5)**		Scoring function											
conformation	Docking program	AnnapuRNA:DL (2013)	AnnapuRNA:DL (2016)	AnnapuRNA:kNN (2013)	AnnapuRNA:kNN (2016)	internal SF	LigandRNA (basic)	LigandRNA (updated)	rDock (dock)	rDock (dock_solv)	RF-Score-VS v2	[RMSD]	**Average**
3D: Open Babel	AutodockVina	8.29	8.45	8.41	8.49	8.93	8.48	8.52	8.41	8.33	8.75	7.55	**8.42**
	iDock	8.42	8.28	8.38	8.43	8.28	8.48	8.48	7.96	8.12	8.67	6.98	**8.23**
	rdock-dock	4.52	4.24	4.31	4.52	4.87	4.96	5.04	4.87	4.93	6.30	3.12	**4.70**
	rdock-dock_solv	4.45	4.22	4.08	4.19	4.92	4.94	4.87	4.66	4.92	5.74	2.74	**4.52**
3D: Balloon	AutodockVina	8.59	8.26	8.64	8.56	8.59	8.77	8.78	8.26	8.35	8.82	7.64	**8.48**
	iDock	8.19	8.63	8.22	8.19	8.92	9.04	9.09	8.03	8.25	8.29	6.97	**8.35**
	rdock-dock	4.38	4.39	4.27	4.18	4.81	4.61	5.01	4.81	4.43	6.33	2.86	**4.55**
	rdock-dock_solv	4.06	4.35	4.11	4.14	4.63	4.68	4.79	5.00	4.63	6.06	2.72	**4.47**
Native Conformation	AutodockVina	8.25	8.36	8.28	8.22	8.61	8.47	8.48	7.86	7.84	8.60	7.58	**8.23**
	iDock	8.11	8.16	8.48	8.21	8.42	8.23	8.19	7.56	7.76	8.45	6.75	**8.03**
	rdock-dock	4.37	4.15	4.14	4.35	5.23	4.92	5.00	5.23	5.12	6.27	2.90	**4.70**
	rdock-dock_solv	3.96	3.69	4.09	4.05	4.59	4.46	4.47	5.19	4.59	6.14	2.58	**4.35**
