# Supplementary material for: AnnapuRNA: A scoring function for predicting RNA-small molecule binding poses
Source: PLoS Comput Biol. 2021 Feb 1;17(2):e1008309. doi: 10.1371/journal.pcbi.1008309 (PMC7877745; doi:10.1371/journal.pcbi.1008309)
Supplement: S19 Table — Docking was performed using rDock with the dock desolvation potential with the native conformation of a ligand as an input. (PDF) [file pcbi.1008309.s036.pdf]

AnnapuRNA	clustering	Best pose	S(3)	S(5)
DL (2013)	no clustering	5.393	4.583	4.369
	clustering	5.393	4.441	4.038
	clustering+averaging	5.331	4.407	3.993
	clustering+averaging+localopt	5.390	4.455	4.028
DL (2016)	no clustering	6.683	4.824	4.148
	clustering	6.683	4.645	3.972
	clustering+averaging	6.662	4.597	3.917
	clustering+averaging+localopt	6.679	4.628	3.952
kNN (2013)	no clustering	6.059	4.855	4.138
	clustering	6.059	4.452	3.959
	clustering+averaging	6.038	4.434	3.945
	clustering+averaging+localopt	6.069	4.459	3.966
kNN (2016)	no clustering	5.759	5.003	4.348
	clustering	5.759	4.593	3.986
	clustering+averaging	5.755	4.586	4.007
	clustering+averaging+localopt	5.790	4.628	4.038
All AnnapuRNA	no clustering	5.973	4.816	4.251
variants	clustering	5.973	4.533	3.989
(average)	clustering+averaging	5.947	4.506	3.966
	clustering+averaging+localopt	5.982	4.542	3.996
