# Supplementary material for: AnnapuRNA: A scoring function for predicting RNA-small molecule binding poses
Source: PLoS Comput Biol. 2021 Feb 1;17(2):e1008309. doi: 10.1371/journal.pcbi.1008309 (PMC7877745; doi:10.1371/journal.pcbi.1008309)
Supplement: S20 Table — (PDF) [file pcbi.1008309.s037.pdf]

		processing time, seconds	
conformation	method	mean	median
3D-babel	AnnapuRNA DL (2013)	111.86	107
	AnnapuRNA kNN (2013)	91.28	91
	LigandRNA (2013)	573.59	404
3D-balloon	AnnapuRNA DL (2013)	112.48	109
	AnnapuRNA kNN (2013)	91.66	92
	LigandRNA (2013)	510.93	412
Native conformation	AnnapuRNA DL (2013)	112.79	110
	AnnapuRNA kNN (2013)	91.38	93
	LigandRNA (2013)	564.55	320
**Average**	AnnapuRNA DL (2013)	112.38	109
**for all methods**	AnnapuRNA kNN (2013)	91.44	92
	LigandRNA (2013)	549.69	378
