# Supplementary material for: AnnapuRNA: A scoring function for predicting RNA-small molecule binding poses
Source: PLoS Comput Biol. 2021 Feb 1;17(2):e1008309. doi: 10.1371/journal.pcbi.1008309 (PMC7877745; doi:10.1371/journal.pcbi.1008309)
Supplement: S21 Table — Additional rows represent the internal scoring function of the docking program (Internal SF). All docking was performed with native conformation of a ligand as an input. (PDF) [file pcbi.1008309.s038.pdf]

docking_program	scoringFunction	Spearman's rank correlation coefficient		Pearson correlation coefficient		Kendall rank correlation coefficient	
		mean	median	mean	median	mean	median
Autodock Vina	AnnapuRNA DL (2013)	0.107	0.077	0.107	0.079	0.079	0.080
	AnnapuRNA DL (2016)	0.086	0.065	0.091	0.039	0.061	0.053
	AnnapuRNA kNN (2013)	0.086	0.029	0.084	0.077	0.059	0.059
	AnnapuRNA kNN (2016)	0.109	0.070	0.098	0.094	0.080	0.080
	Internal SF	0.068	0.039	0.081	0.049	0.049	0.029
	LigandRNA (2013)	0.168	0.198	0.166	0.299	0.128	0.149
	LigandRNA (updated)	0.174	0.167	0.168	0.301	0.131	0.128
	RF-Score-VS v2	0.075	0.077	0.064	0.060	0.054	0.043
	rDock (dock)	0.318	0.257	0.343	0.344	0.252	0.172
	rDock (dock_solv)	0.321	0.274	0.355	0.360	0.259	0.207
iDock	AnnapuRNA DL (2013)	0.130	0.071	0.078	0.035	0.097	0.054
	AnnapuRNA DL (2016)	0.120	0.058	0.076	0.046	0.089	0.034
	AnnapuRNA kNN (2013)	0.124	0.082	0.083	0.050	0.093	0.059
	AnnapuRNA kNN (2016)	0.127	0.090	0.083	0.052	0.095	0.061
	Internal SF	0.097	0.076	0.086	0.111	0.070	0.057
	LigandRNA (2013)	0.207	0.230	0.217	0.220	0.154	0.154
	LigandRNA (updated)	0.209	0.228	0.218	0.227	0.156	0.154
	RF-Score-VS v2	0.064	0.097	0.079	0.141	0.048	0.071
	rDock (dock)	0.335	0.265	0.317	0.290	0.258	0.192
	rDock (dock_solv)	0.344	0.265	0.314	0.223	0.264	0.187
rDock (dock)	AnnapuRNA DL (2013)	0.339	0.313	0.372	0.365	0.250	0.209
	AnnapuRNA DL (2016)	0.345	0.380	0.372	0.372	0.253	0.260
	AnnapuRNA kNN (2013)	0.333	0.350	0.368	0.355	0.244	0.243
	AnnapuRNA kNN (2016)	0.336	0.371	0.367	0.362	0.246	0.243
	Internal SF	0.116	0.087	0.107	0.031	0.080	0.065
	LigandRNA (2013)	0.244	0.287	0.279	0.312	0.181	0.207
	LigandRNA (updated)	0.244	0.280	0.279	0.314	0.181	0.207
	RF-Score-VS v2	-0.029	-0.088	-0.012	-0.080	-0.022	-0.056

	rDock (dock)	0.117	0.088	0.107	0.031	0.081	0.065
	rDock (dock_solv)	0.102	0.103	0.099	0.057	0.071	0.072
rDock (dock_solv)	AnnapuRNA DL (2013)	0.321	0.378	0.379	0.405	0.230	0.266
	AnnapuRNA DL (2016)	0.326	0.366	0.378	0.388	0.241	0.251
	AnnapuRNA kNN (2013)	0.311	0.367	0.367	0.390	0.227	0.259
	AnnapuRNA kNN (2016)	0.311	0.357	0.365	0.397	0.226	0.221
	Internal SF	0.101	0.048	0.126	0.100	0.071	0.032
	LigandRNA (2013)	0.268	0.249	0.302	0.303	0.196	0.170
	LigandRNA (updated)	0.269	0.246	0.302	0.296	0.196	0.169
	RF-Score-VS v2	0.047	0.021	0.029	0.010	0.032	0.029
	rDock (dock)	0.068	-0.005	0.068	0.017	0.048	-0.002
	rDock (dock_solv)	0.102	0.048	0.123	0.100	0.071	0.032
