# Supplementary material for: AnnapuRNA: A scoring function for predicting RNA-small molecule binding poses
Source: PLoS Comput Biol. 2021 Feb 1;17(2):e1008309. doi: 10.1371/journal.pcbi.1008309 (PMC7877745; doi:10.1371/journal.pcbi.1008309)
Supplement: S1 Text — Optimization of parameters of the scoring function; post-processing of docking poses; additional information on docking algorithms. (PDF) [file pcbi.1008309.s039.pdf]

Optimization of parameters of the scoring function

In the course of optimization of parameters of AnnapuRNA, to assess the influence of the tested parameters on the performance of the scoring function, we considered the previously used metric *S*(3), which reports the lowest RMSD to the reference ligand among three top-scoring poses. To avoid overfitting of the mathematical models, we used a cross-validation scheme with five splits of data according to the ligand structural class. Firstly, ligands were clustered into five groups, using RDKit fingerprint Tanimoto similarity and *k*-Medoids clustering method, representing five chemical classes of compounds: 1) amino acids and carboxylic acids, 2) heterocycles and polycyclic compounds, 3) polysugars, 4) amines, 5) alcohols and polyols (see Fig S8 for the ligand similarity matrix). Each variant of AnnapuRNA scoring function was trained using data from four clusters and tested on the data coming from the remaining cluster. This training and testing procedure was repeated for all five combinations of clusters and the scoring performance was averaged. At each cross-validation split data were class-balanced, i.e, both classes were represented by an equal number of cases. All parameter optimizations were performed on the 2013 training dataset.

Machine learning methods to build mathematical interactions models.
To derive the scoring models from the collected multidimensional interactions statistics, we applied Machine Learning methods. This approach was previously successfully applied to many problems in bioinformatics, including modeling of protein-ligand interactions [1][2][3][4][5] and drug discovery (for a recent review, see: [6]. To find the best initial modeling approach we tested five machine learning algorithms (Deep Learning - multi-layer feedforward artificial neural network - DL, Gaussian Naïve Bayes - GNB, *k* Nearest Neighbors - kNN, Random Forests - RF, and Support Vector Machines with RBF kernel - SVM). For each machine learning method, for each contact group, we used a grid search algorithm to automatically scan the parameter space of individual algorithms and select the best combination of them (see Table S5 for parameters details). Next, for the optimized set of parameters, individual models were built and used in the scoring function.

In parallel, we tested the influence of the interaction distance cut-off value, below which we consider RNA pseudoatoms and ligand pharmacophores to be in contact. We analyzed four cut-off values 6, 8, 10, and 12 Å, where the maximal value is two times larger than the cut-off used earlier by Philips et al. (See: Table S6). We observed that the performance of the scoring functions, for all classifiers, is the best for the 10 Å cut-off distance, with the average S(3) = 4.89. The slightly worse performance was observed for 8 Å and 12 Å cut-offs, with S(3) equal to 5.12 and 5.26 respectively. As for the classifiers, at 10 Å cut-off distance, the best cross-validation results were obtained for kNN classifier, with S(3) = 4.68. For further parameters optimizations, we decided to use 10 Å interaction distance cutoff with two best performing classifiers: kNN and DL.

We compared using balanced data (i.e., with a reduced number of observations, so both classes are represented by an equal number of cases) for building models with a case when non-balanced data is used. For the DL method, the average *S*(3) value from cross-validation dropped from 4.95 to 5.11, so we decided to include data balancing in our pipeline.

Data preprocessing
We tested the influence of various ways of preprocessing input data, distance, and angles, on the performance of AnnapuRNA scoring function variants. We performed a cross-validation experiment using training data with normalized distance values binned at various steps, together

with transformed values angles. For angle values transformation, we applied binning or cosine transformation and then binning. We observed that there is no evident influence of the discretization of the training data on the performance of a derived scoring function (Table S7). For functions developed using the DL method we observed a slight increase in performance for smaller values of bins (0.01 - 0.02 for distances and 0.005 - 0.010 for angles), while for the kNN method we observed the opposite trend. We concluded that discretization did not uniformly improve the accuracy of predictions and hence we decided not to use it in our data preprocessing pipeline.

Distance dependance of interaction probabilities

In the interaction models tested above, we assumed that the contributions from all pairs of pseudoatoms within the interaction range (set at the value of 10 Å) to the final score are equally important (see: equation 2). We evaluated the performance of the scoring functions changes if a distant-dependent weights are applied to the component probability values, calculated for each of the interactions. This transformation expresses the higher contribution of the short-range interactions and lower for the more distant ones. For this purpose we introduced to equation 2 an additional distant-dependent weight factor *w(d)* (see: equation 4).

$$E_{RNA-Ligand} = -1 \sum_{interactions} w(d) \cdot p(interaction) \quad (4)$$

We tested three different transforming functions: multiplicative inverse (equation 5), Lennard-Jones-like transformation (equation 6) and linear transformation (equation 7)

$$w(d) = \frac{1}{d} \quad (5) \qquad w(d) = -1 \cdot \left[\left(\frac{0.3}{d}\right)^{12} - 2 \cdot \left(\frac{0.3}{d}\right)^{6}\right] \quad (6) \qquad w(d) = -1 \cdot d + 1 \quad (7)$$

After cross-validation experiment we found that the weighting function, counterintuitively, has a negative effect on the performance of both scoring functions (See: Table S8). We interpret it that in our interaction models all contacts, regardless of the distance, have a similar contribution to the final score, or that other factors may influence this contribution (such as the microenvironment in the binding pocket). Although the weight factor *w(d)* is omitted in the scoring program by default (i.e., *w(d)* = 1), the user may still turn it on with a command line argument.

Input data noise removal

We examined two methods of data preprocessing aiming at removing class label noise: Tomek's links [7] and Edited Nearest Neighbors (ENN, [8]) implemented in imbalanced-learn python package [9]. This experiment showed that application of both: Tomek's links and ENN methods to the input data improved the final performance of the scoring function in comparison to no data preprocessing, with ENN giving slightly better results, and thus ENN was included into the data preprocessing pipeline (see: Table S9).

Ligand term weight

We designed the final score of the complex to be a sum of terms of RNA-Ligand interactions and Ligand internal energy (equation 1). For the latter, we used Generalized Amber Force Field (GAFF), which has a set of parameters for most small drug-like molecules. During optimization, we also tested the Merck Molecular Force Field (MMFF94, [10]), but for a testing set consisting of the

diversified set of experimentally determined ligand structures deposited in the PDB database this method failed to calculate energy values for a substantial number of ligands. We also introduced a weighting factor w, which reflects the contribution of the Ligand's term to the final complex's score (equation 3). We tested the influence of the value of this parameter on the performance of scoring functions in a cross-validation experiment (see: Table S10). We noticed that for both scoring functions, there is no clear influence of w parameter on the performance expressed as *S*(1) and *S*(3). Both of these values fluctuate with an increasing value of *w*. Finally, we decided to set a default value for this parameter to 0.1, which was also selected in the Pareto ranking, as a reasonable balance between *S*(1) and *S*(3) performance. The relatively small value of w is also limiting the influence of a ligand term on the final score of a complex. The w parameter can be changed by the user in a command-line option and thus adjusted to the nature of the input data.

Post-processing of docking poses

In the current version of AnnapuRNA, we implemented three optional steps for the post-processing of poses from docking or rescoring, namely clustering, centroid calculation, and local optimization.

The first step is the clustering of poses based on the RMSD distance matrix. This operation serves to define distinct binding groups of ligand poses and it has been shown useful for processing many types of docking data [11,12]. We implemented three clustering algorithms that take the RMSD distance matrix as an input, namely `AutoDock-like' method (as implemented in the AutoDock/AutoDock Vina), `SimRNA-like' method (as implemented in ROSETTA/SimRNA programs), and Affinity Propagation method. All methods start from a RMSD distance matrix; two of them are based on a distance cutoff, the third one is based on the Affinity Propagation algorithm; see: materials and methods for details). The second step of post-processing, aimed to be combined with clustering, is the calculation of the cluster centroid, i.e. averaging x, y and z coordinates of the corresponding atoms for all poses in a given cluster), later referred to as the "averaging". As has been shown earlier, cluster centroids maye be more similar to the reference structure in terms of a better score (RMSD, TM-score), but usually are structurally distorted and should be refined [11,13]. In AnnapuRNA it is the third postprocessing step, which involves a local geometry optimization of the averaged poses (later referred to as the "localopt").

The average improvement of performance while these post-processing methods are employed is clearly visible for *S*(3) parameters. For example, in comparison to the performance of unprocessed results (with the average *S*(3) for all methods equal 4.816 Å), both - the clustering and structure averaging improved the performance (with the average *S*(3) for all methods equal 4.533 Å and 4.506 Å, respectively; data for for `AutoDock-like' clustering, for details, see Table S19). Additional geometry optimization of the ligand resulted in slightly lower performance in comparison to the averaged structures (the average *S*(3) for all methods equal to 4.542 Å) which is still better than for the unprocessed data. On the other hand, while considering *S*(1) metrics, on average these post-processing methods did not spectacularly improve the performance of AnnapuRNA scoring functions. For example, the average RMSD of the best identified pose, for all AnnapuRNA functions without postprocessing was 5.973 Å, while for clustering and averaging structure was only slightly better (5.947 Å), and when paired with a local optimization of the geometry - slightly worse (5.982 Å). For this clustering method the ligand with the best score is the member of the main cluster, thus the best pose identified by the method without and with clustering is the same.

This means that by employing optional post-processing steps one may obtain a physically reliable model of RNA-ligand complex which is closer to the native one than the initial structure from docking (clustering and clustering+averaging+localopt) or even better model with lower

RMSD to the reference, which may not be physically reliable (clustering+averaging). Sample results of poses selected during docking and postprocessing are shown in the Figure S15**.**

Additional information

A summary of a ligand conformation sampling methods and initial ligand positioning used by docking programs.

rDock

Conformational sampling. rDock utilizes a combination of stochastic and deterministic search techniques to generate low energy ligand poses. The standard docking protocol to generate a single ligand pose uses three stages of Genetic Algorithm search (GA), followed by low temperature Monte Carlo and Simplex minimization stages. The GA stages are interdependent and are designed to be used sequentially. Only samples exocyclic dihedral angles, a correct input geometry is required for bonds, angles and rings. In the case of flexible rings, a variety of low-energy conformers should be generated prior to docking.

Initial ligand position and conformation. The ligand centre of mass is constrained to lie on a randomly selected grid point within the defined docking volume and the ligand orientation and all dihedral angles
are picked randomly.

iDock

Conformational sampling. iDock makes use of Monte Carlo algorithm for global optimization and Broyden-Fletcher-Goldfarb-Shanno (BFGS) Quasi-Newton method for local optimization. A succession of steps consisting of a mutation and a BFGS local optimization are taken, with each step being accepted according to the Metropolis criterion. These steps are repeated over N iterations, where N correlates to the complexity of the ligand regarding number of non-hydrogen atoms and number of torsions.

Initial ligand position and conformation. Start from a random initial ligand conformation.

AutoDock Vina

Conformational sampling. AutoDock Vina has implemented an Iterated Local Search global optimizer, in which a succession of steps consisting of a mutation and a local optimization are taken, with each step being accepted according to the Metropolis criterion. It also makes use of a BFGS method for the local optimization. It is a function of the position and orientation of the ligand, as well as the values of the torsions for the active rotatable bonds in the ligand.

Initial ligand position and conformation. Start from a random conformation of the ligand, including its position, orientation, and torsions.

References

1. Ain QU, Aleksandrova A, Roessler FD, Ballester PJ. Machine-learning scoring functions to improve structure-based binding affinity prediction and virtual screening. Wiley Interdiscip Rev Comput Mol Sci. 2015;5: 405–424.

2. Ballester PJ. Machine Learning for Molecular Modelling in Drug Design. Biomolecules. 2019. p. 216. doi:10.3390/biom9060216

3. Colwell LJ. Statistical and machine learning approaches to predicting protein–ligand interactions. Current Opinion in Structural Biology. 2018. pp. 123–128. doi:10.1016/j.sbi.2018.01.006

4. Khamis MA, Gomaa W. Comparative assessment of machine-learning scoring functions on PDBbind 2013. Engineering Applications of Artificial Intelligence. 2015. pp. 136–151. doi:10.1016/j.engappai.2015.06.021

5. Wójcikowski M, Ballester PJ, Siedlecki P. Performance of machine-learning scoring functions in structure-based virtual screening. Sci Rep. 2017;7: 46710.

6. Yang X, Wang Y, Byrne R, Schneider G, Yang S. Concepts of Artificial Intelligence for Computer-Assisted Drug Discovery. Chem Rev. 2019;119: 10520–10594.

7. Two Modifications of CNN. IEEE Transactions on Systems, Man, and Cybernetics. 1976. pp. 769–772. doi:10.1109/tsmc.1976.4309452

8. Wilson DL. Asymptotic Properties of Nearest Neighbor Rules Using Edited Data. IEEE Transactions on Systems, Man, and Cybernetics. 1972. pp. 408–421. doi:10.1109/tsmc.1972.4309137

9. Lemaître G, Nogueira F, Aridas CK. Imbalanced-learn: A Python Toolbox to Tackle the Curse of Imbalanced Datasets in Machine Learning. J Mach Learn Res. 2017;18.1: 559–563.

10. Halgren TA. Merck molecular force field. I. Basis, form, scope, parameterization, and performance of MMFF94. Journal of Computational Chemistry. 1996. pp. 490–519. doi:3.0.co;2-p">10.1002/(sici)1096-987x(199604)17:5/6<490::aid-jcc1>3.0.co;2-p

11. Lorenzen S, Zhang Y. Identification of near-native structures by clustering protein docking conformations. Proteins. 2007;68: 187–194.

12. Bottegoni G, Cavalli A, Recanatini M. A Comparative Study on the Application of Hierarchical−Agglomerative Clustering Approaches to Organize Outputs of Reiterated Docking Runs. Journal of Chemical Information and Modeling. 2006. pp. 852–862. doi:10.1021/ci050141q

13. Zhang Y. I-TASSER server for protein 3D structure prediction. BMC Bioinformatics. 2008;9:
